# Supplementary material for: Reversible modulation of circadian time with chronophotopharmacology
Source: Nat Commun. 2021 May 26;12:3164. doi: 10.1038/s41467-021-23301-x (PMC8155176; doi:10.1038/s41467-021-23301-x)
Supplement: Supplementary file 1 — Supplementary Information [file 41467_2021_23301_MOESM1_ESM.pdf]

Supplementary Information

Reversible Modulation of Circadian Time with Chronophotopharmacology

Dušan Kolarski[1], Carla Miró-Vinyals[1,‡], Akiko Sugiyama[2,‡], Ashutosh Srivastava[2], Daisuke Ono[3], Yoshiko Nagai[2], Mui Iida[2,4], Kenichiro Itami[2,5], Florence Tama[2,6,7], Wiktor Szymanski[1,8]*, Tsuyoshi Hirota[2,4]*, Ben L. Feringa[1,]*

[1] Centre for Systems Chemistry, Stratingh Institute for Chemistry, University of Groningen, Nijenborgh 4, 9747 AG, Groningen, The Netherlands

[2] Institute of Transformative Bio-Molecules (WPI-ITbM), Nagoya University, Chikusa, Nagoya 464-8601, Japan

[3] Department of Neuroscience II, Research Institute of Environmental Medicine, Nagoya University, Chikusa, Nagoya, 464-8601, Japan

[4] Division of Biological Science, Graduate School of Science, Nagoya University, Chikusa, Nagoya 464-8601, Japan.

[5] Department of Chemistry, Graduate School of Science, Nagoya University, Chikusa, Nagoya 464-8601, Japan.

[6] Department of Physics, Graduate School of Science, Nagoya University, Chikusa, Nagoya 464-8601, Japan

[7] Computational Structural Biology Unit, RIKEN-Center for Computational Science, Kobe, Hyogo 650-0047, Japan

[8] University Medical Center Groningen, Department of Radiology, Medical Imaging Center, University of Groningen, Hanzeplein 1, 9713 GZ Groningen, The Netherlands

Contents

Chemical synthesis 4

Photostationary state (PSS) distribution determination 18

Compound **3** 18

Compound **4** 19

Compound **5** 20

Compound **6** 21

Compound **7** 22

Compound **8** 23

Compound **9** 24

LCMS traces of stability studies 25

Compound **3** 25

Compound **4** 26

Compound **5** 27

Compound **6** 28

Compound **7** 29

Compound **8** 30

Compound **9** 31

Half-life measurements 32

Compound **3** 32

Compound **4** 33

Compound **5** 34

Compound **6** 35

Compound **7** 36

Compound **8** 37

Half-life comparison of **6**, **7** and **8** 38

Compound **9** 39

Solubility test 40

In vitro kinase assay 41

Reagents 41

Data analysis 41

Evaluation of the circadian period photo-modulation 43

UV-vis absorption spectra and fatigue studies 48

Luciferin 48

Compound **3** 48

Compound **4** 49

Compound **5** 49

Compound **6** 50

Compound **7** 50

Compound **8** 51

Compound **9** 51

Molecular docking 52

Molecular docking analysis of the photoinduced differences in binding 52

NMR and HRMS spectra 57

Supplementary References 89

Chemical synthesis

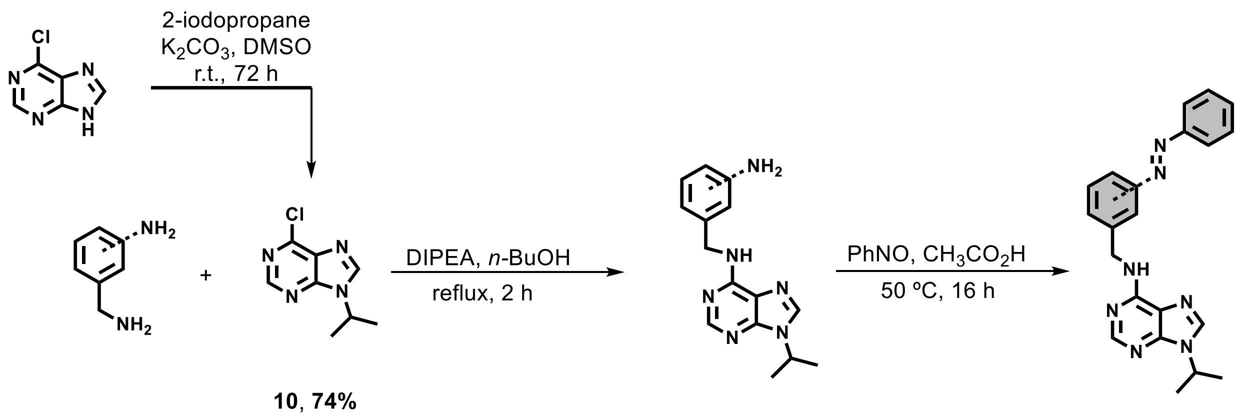

10, **74%**

11: *o*-NH_2, **50%**
12: *m*-NH_2, **82%**
13: *p*-NH_2, **61%**

3: *p*-Azo, **75%**
4: *m*-Azo, **62%**
5: *o*-Azo, **83%**

6-chloro-9-isopropyl-9H-purine (**10**)

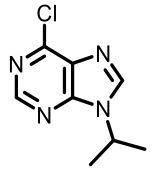

6-chloro-9H-purine (12.0 g, 77.6 mmol, 5.00 equiv) was added together with 2-iodopropanone (1.6 ml, 16 mmol, 1.0 equiv) to DMSO (200 mL) and fine K_2CO_3 powder (21.0 g, 15.6 mmol, 1.00 equiv) was added. The reaction was stirred for three days at room temperature. Subsequently, half of the solvent was evaporated and the precipitate was filtered out and washed with ethyl acetate. The filtrate was evaporated and the product was crashed out by adding cold water to the DMSO solution. The product was obtained as a light-yellow solid (74%, 11.3 g, 57.4 mmol).

^1H NMR (400 MHz, $CDCl_3$) δ 8.74 (s, 1H), 8.18 (s, 1H), 4.94 (hept, J = 6.8 Hz, 1H), 1.67 (d, J = 6.8 Hz, 6H) ppm. As in literature.[1]

N-(2-aminobenzyl)-9-isopropyl-9H-purin-6-amine (**11**)

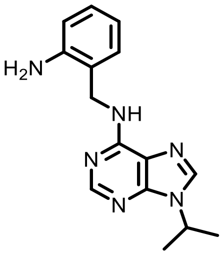

To a round-bottomed flask compound **10** (1.3 g, 6.7 mmol, 1.0 equiv), 2-(aminomethyl)aniline (1.6 g, 13 mmol, 2.0 equiv), and DIPEA (5.8 ml, 34 mmol, 5.0 equiv) were stirred under reflux in *n*-BuOH (130 ml) for 3 h. The solvent was evaporated and the

crude was submitted to a column chromatography (SiO_2; DCM:MeOH 95:5). The final product was obtained as a light-yellow solid in 50% yield (0.95 g, 3.4 mmol).

^{1}H NMR (400 MHz, $CDCl_3$) δ 8.41 (s, 1H), 7.68 (s, 1H), 7.19 – 7.04 (m, 2H), 6.76 – 6.62 (m, 2H), 6.37 (d, J = 6.3 Hz, 1H), 4.87 – 4.80 (m, 1H), 4.46 (s, 2H), 1.58 (d, J = 6.8 Hz, 6H) ppm. As in literature.[2]

N-(3-aminobenzyl)-9-isopropyl-9H-purin-6-amine (**12**)

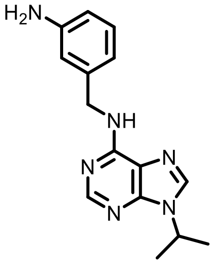

To a round-bottomed flask compound **10** (1.3 g, 6.8 mmol, 1.0 equiv) was added together with 3-(aminomethyl)aniline (1.0 g, 8.2 mmol, 1.2 equiv) and *N*,*N*-diisopropylethylamine (DIPEA) (5.9 ml, 34 mmol, 5.0 equiv) in *n*-BuOH (70 mL). The mixture was stirred under reflux for 2 h. *n*-BuOH was evaporated and the crude product was submitted to a column chromatography (SiO_2; DCM:MeOH 95:5). The final product was obtained as a light-yellow solid in 82% yield (1.6 g, 5.6 mmol).

^{1}H-NMR (400 MHz, $CDCl_3$) δ 8.42 (s, 1H), 7.79 (s, 1H), 7.12 (t, J = 7.8 Hz, 1H), 6.77 (d, J = 7.6 Hz, 1H), 6.71 (s, 1H), 6.62 – 6.58 (m, 1H), 6.03 (s, 1H), 4.83 (td, J = 13.2, 6.3 Hz, 3H), 3.65 (s, 2H), 1.61 (d, J = 6.8 Hz, 6H) ppm. ^{13}C NMR (101 MHz, $CDCl_3$) δ 154.7, 152.9, 146.7, 139.7, 137.3, 129.6, 120.1, 117.8, 114.2, 46.9, 41.0, 22.7 ppm. Two carbon signals are missing. FTIR $\tilde{\nu}$ 3455, 3341, 2909, 1612, 646 cm^{-1}. HRMS (ESI$^+$) calc. for $C_{15}H_{18}N_6$ $[M+H]^+$: 283.1665, found 283.1664. m.p. = 124-127 °C.

N-(4-aminobenzyl)-9-isopropyl-9H-purin-6-amine (**13**)

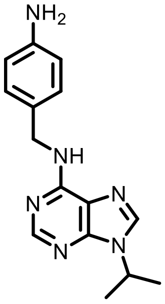

To a round-bottomed flask, compound **1** (1.3 g, 6.8 mmol, 1.0 equiv) was added together with 4-(aminomethyl)aniline (1.0 g, 8.2 mmol, 1.2 equiv) and *N*,*N*-diisopropylethylamine (DIPEA) (5.9 ml, 34 mmol, 5.0 equiv) in *n*-BuOH (70 mL). The mixture was stirred under reflux for 2 h. *n*-BuOH was evaporated and the residue was submitted to a column chromatography (SiO_2; DCM:MeOH 95:5). The final product was obtained as a light-yellow solid in 61% yield (1.1 g, 4.0 mmol).

^{1}H NMR (400 MHz, $CDCl_3$) δ 8.37 (s, 1H), 7.68 (s, 1H), 7.13 (d, *J* = 8.3 Hz, 1H), 6.60 (d, *J* = 8.4 Hz, 1H), 4.80 (hept, *J* = 6.8 Hz, 1H), 4.73 (s, 2H), 3.66 (s, 3H), 1.55 (d, *J* = 6.8 Hz, 6H) ppm. ^{13}C NMR (101 MHz, $CDCl_3$) δ 145.8, 137.4, 129.1, 128.8, 128.2, 119.2, 115.3, 115.2, 113.0, 48.0, 47.0, 22.7 ppm. FTIR $\tilde{\nu}$ 3322, 3216, 2974, 2930, 1609, 1516, 1325, 1226, 1064, 795, 645 cm^{-1}. HRMS (ESI^+) calc. for $C_{15}H_{18}N_6$ $[M+H]^+$: 283.1665, found 283.1660. m.p. = 127-129 °C.

(*E*)-9-isopropyl-*N*-(4-(phenyldiazenyl)benzyl)-9H-purin-6-amine (**3**)

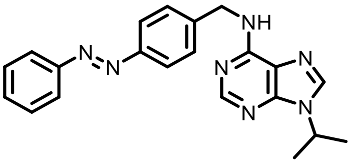

Compound **13** (0.78 g, 2.8 mmol, 1.0 equiv) was dissolved in acetic acid (15 ml), and nitrosobenzene (0.42 g, 3.9 mmol, 1.5 equiv) was added. The reaction mixture was stirred at 50 °C for 24 h. Acetic acid was evaporated under reduced pressure, the residue was dissolved in EtOAc and the organic layer was washed with saturated aq. $NaHCO_3$ solution, water and brine. The organic layer was dried over $MgSO_4$, evaporated and the product was purified by column chromatography (SiO_2; DCM/MeOH 95:5 → 9:1) to yield **3** as an orange solid (75%, 0.8 g, 2.1 mmol).

^{1}H-NMR (400 MHz, $CDCl_3$) *δ* 8.43 (s, 1H), 7.94 – 7.87 (m, 4H), 7.78 (s, 1H), 7.57 – 7.42 (m, 5H), 6.30 (s, 1H), 4.99 (s, 2H), 4.85 (hept, J = 6.8 Hz, 1H), 1.61 (d, J = 6.8 Hz, 6H) ppm. ^{13}C NMR (101 MHz, $CDCl_3$) *δ* 152.8, 152.8, 152.6, 152.0, 141.8, 137.5, 131.0, 129.1, 128.3, 123.2, 122.8, 47.0, 44.0, 22.7 ppm. Two carbon signals are missing. FTIR $\tilde{\nu}$ 3266, 3217, 3291, 3146, 2911, 1622, 688 cm^{-1}. HRMS (ESI^+) calc. for $C_{21}H_{21}N_7$ $[M+H]^+$: 372.1931, found 372.1929. m.p. = 131-132 °C.

(*E*)-9-isopropyl-*N*-(3-(phenyldiazenyl)benzyl)-9H-purin-6-amine (**4**)

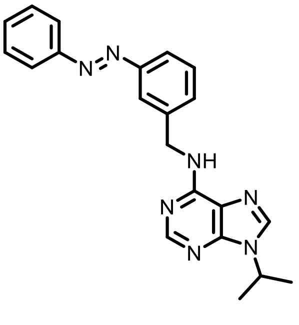

Compound **12** (0.6 g, 2.1 mmol, 1.0 equiv) was dissolved in acetic acid (12 ml), and nitrosobenzene (0.3 g, 3.2 mmol, 1.5 equiv) was added. The reaction mixture was stirred at 50 °C for 24 h. Acetic acid was evaporated under reduced pressure, the crude mixture was dissolved in EtOAc and the organic layer was washed with saturated aq. $NaHCO_3$ solution, water and brine. The organic layer was dried over $MgSO_4$, evaporated and the product was purified by column chromatography (SiO_2; DCM/MeOH 95:5 → 9:1) to yield **4** as an orange solid (62%, 0.5 g, 1.3 mmol).

^{1}H-NMR (400 MHz, DMSO-*d₆*) *δ* 8.44 (s, 1H), 7.95 – 7.86 (m, 3H), 7.83 – 7.74 (m, 2H), 7.51 – 7.42 (m, 2H), 7.05 – 6.96 (m, 2H), 6.24 (s, 1H), 4.99 (s, 2H), 4.91 – 4.77 (m, 1H), 3.89 (s, 3H),

1.60 (d, J = 6.8 Hz, 6H). ^{13}C NMR (101 MHz, $CDCl_3$) δ 154.6, 152. 9, 152.6, 140.0, 137.5, 131.0, 130.2, 129.4, 129.1, 122.8, 122.0, 47.0, 22.7. Two carbon signals are missing. FTIR $\tilde{\nu}$ 3260, 3135, 3052, 2976, 1614, 690 cm^{-1}. HRMS (ESI^+) calc. for $C_{21}H_{21}N_7$ $[M+H]^+$: 372.1931, found 372.1933. m.p. = 112-114 °C.

(*E*)-9-isopropyl-*N*-(2-(phenyldiazenyl)benzyl)-9H-purin-6-amine (**5**)

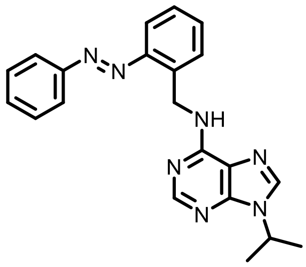

Compound **11** (0.3 g, 1.1 mmol, 1.0 equiv) was dissolved in acetic acid (7 ml), and nitrosobenzene (0.2 g, 1.7 mmol, 1.5 equiv) was added. The reaction mixture was stirred at 50 °C for 24 h. Acetic acid was evaporated under reduced pressure, the crude mixture was dissolved in EtOAc and the organic layer was washed with saturated aq. $NaHCO_3$ solution, water and brine. The organic layer was dried over $MgSO_4$, evaporated and the product was purified by column chromatography (SiO_2; DCM/MeOH 95:5 → 9:1) to yield **5** as an orange solid (83%, 0.4 g, 0.9 mmol).

^1H NMR (400 MHz, $CDCl_3$) δ 8.43 (s, 1H), 7.95 – 7.87 (m, 2H), 7.76 – 7.67 (m, 2H), 7.63 (dd, J = 7.6, 1.6 Hz, 1H), 7.54 – 7.44 (m, 3H), 7.44 – 7.32 (m, 2H), 6.59 (s, 1H), 5.43 (s, 2H), 4.80 (hept, J = 6.8 Hz, 1H), 1.56 (d, J = 6.8 Hz, 6H) ppm. ^{13}C NMR (101 MHz, $CDCl_3$) δ 154.7, 152.8, 152.8, 150.2, 137.6, 137.2, 131.3, 131.2, 129.8, 129.1, 128.3, 123.1, 116.0, 46.9, 40.6, 22.7 ppm. Two carbon signals are missing. FTIR $\tilde{\nu}$ 3219, 3130, 3051, 2978, 1618, 770, 684 cm^{-1}. HRMS (ESI^+) calc. for $C_{21}H_{21}N_7$ $[M+H]^+$: 372.1931, found 372.1925. m.p. = 114-117 °C.

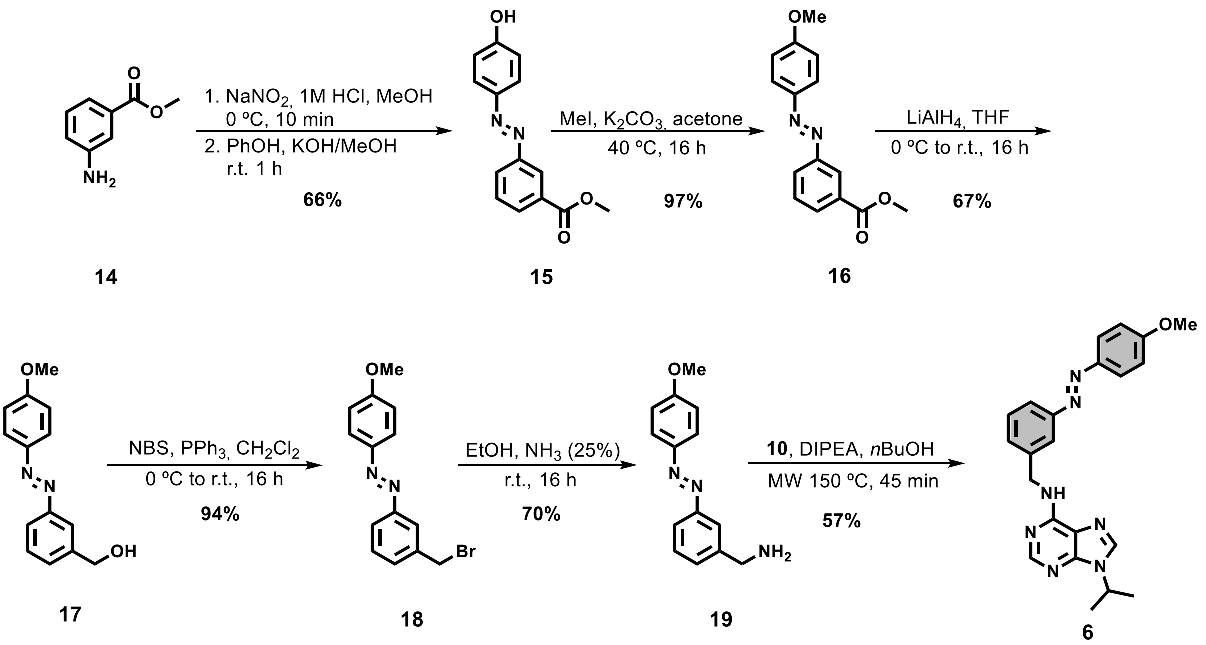

Methyl (*E*)-3-((4-hydroxyphenyl)diazenyl)benzoate (**15**)

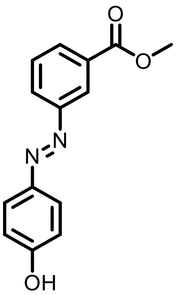

Methyl 3-aminobenzoate (**14**, 2.57 g, 17.5 mmol, 1.20 equiv) was dissolved in 1 M aq. solution of HCl and cooled down in an ice bath. Subsequently, $NaNO_2$ (1.60 g, 22.0 mmol, 1.50 equiv) was added and the reaction mixture was stirred for 10 min at 0 °C. Methanol (25 ml) was added followed by the drop-wise addition of phenol (1.93 g, 14.6 mmol, 1.00 equiv) and KOH (2.14 g, 38.0 mmol, 2.60 equiv) solution in methanol (20 ml). The mixture was left stirring for 1 h at room temperature. Work-up was performed by adding 1 M aq. solution of HCl (50 ml) and EtOAc (50 ml). The aqueous layer was extracted with EtOAc (3 x 50 ml), the organic layers were dried and concentrated under vacuum. The residue was triturated by addition of diethyl ether and the precipitate was washed with pentane. The product was obtained as an off-orange solid (66%, 2.47 g, 9.60 mmol).

^{1}H NMR (400 MHz, $CDCl_3$) δ 8.52 (t, J = 1.9 Hz, 1H), 8.11 (dt, J = 7.7, 1.4 Hz, 1H), 8.08 – 8.04 (m, 1H), 7.93 – 7.88 (m, 2H), 7.58 (t, J = 7.8 Hz, 1H), 7.00 – 6.95 (m, 2H), 3.97 (d, J = 0.7 Hz, 3H) ppm. ^{13}C-NMR (101 MHz, DMSO-d_6) δ 166.2, 161.9, 152.6, 145.6, 131.3, 131.1, 130.5, 127.8, 125.7, 122.0, 116.5, 52.9 ppm. FTIR $\tilde{\nu}$ 3310 (br), 3297, 3060, 2965, 1701, 1594 cm^{-1}. HRMS (ESI$^+$) calc. for $C_{14}H_{12}N_2O_3$ $[M+H]^+$: 257.0921, found 257.0919. m.p. = 155-157 °C.

Methyl (*E*)-3-((4-methoxyphenyl)diazenyl)benzoate (**16**)

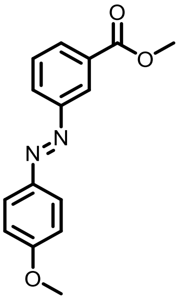

In a round-bottomed flask, compound **15** (3.0 g, 12 mmol, 1.0 equiv) was dissolved in acetone (90 ml) and subsequently methyl iodide (2.5 ml, 41 mmol, 3.5 equiv) and potassium carbonate (16 g, 0.12 mol, 10 equiv) were added. The reaction mixture was stirred for 16 h at 40 °C. After completion of the reaction, diethyl ether and water were added. The organic layer was separated, washed with brine and dried over $MgSO_4$. The product was obtained as an orange solid after removal of the solvent (97%, 3.1 g, 12 mmol).

^{1}H NMR (400 MHz, $CDCl_3$) δ 8.56 – 8.49 (m, 1H), 8.11 (ddd, J = 7.7, 1.7, 1.2 Hz, 1H), 8.06 (ddd, J = 8.0, 2.0, 1.2 Hz, 1H), 7.99 – 7.92 (m, 2H), 7.61 – 7.55 (m, 1H), 7.06 – 6.99 (m, 2H), 3.97 (s, 3H), 3.91 (s, 3H) ppm. ^{13}C NMR (101 MHz, $CDCl_3$) δ 166.7, 162.4, 152.8, 146.9, 131.2, 131.0, 129.1, 126.7, 125.0, 123.7, 114.3, 55.6, 52.3 ppm. FTIR $\tilde{\nu}$ 3076, 2941, 2841, 1717, 1252 cm^{-1}. HRMS (ESI^+) calc. for $C_{15}H_{14}N_2O_3$ $[M+H]^+$: 271.1021, found 271.1077. m.p. = 87-90 °C.

(*E*)-(3-((4-methoxyphenyl)diazenyl)phenyl)methanol (**17**)

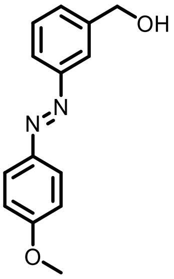

Compound **16** (2.0 g, 7.4 mmol, 1.0 equiv) was dissolved in dry THF under nitrogen in a flame-dried flask. The reaction mixture was cooled down with an ice bath, followed by addition of $LiAlH_4$ solution in THF (1 M, 7.4 ml, 7.4 mmol, 1.0 equiv), which turned the solution immediately green. The reaction mixture was left to slowly warm up to room temperature and left stirred for 16 h. The reaction was quenched by adding methanol (20 ml), EtOAc (200 ml) and sodium tartrate (20 g in 400 ml of water) and the mixture stirred for 1 h. Next, the aqueous layer was extracted with ethyl acetate (3 x 80 ml), the combined organic layers were washed with water, dried with brine and $MgSO_4$ and concentrated under vacuum. The crude product was purified using column chromatography (SiO_2; Pentane/EtOAc → 2:1) and precipitated with pentane. An orange solid was obtained in 66% yield (1.40 g, 5.77 mmol).

^{1}H NMR (400 MHz, $CDCl_3$) δ 7.96 – 7.90 (m, 2H), 7.87 (tq, J = 1.4, 0.6 Hz, 1H), 7.83 – 7.79 (m, 1H), 7.53 – 7.47 (m, 1H), 7.45 (dddd, J = 7.5, 2.0, 1.4, 0.6 Hz, 1H), 7.07 – 6.97 (m, 2H), 4.80 (s,

2H), 3.89 (s, 3H), 1.86 (s, 1H) ppm. ^{13}C NMR (101 MHz, $CDCl_3$) δ 162.1, 153.0, 147.0, 142.0, 129.3, 128.7, 124.8, 122.4, 120.3, 114.2, 65.0, 55.6 ppm. FTIR $\tilde{\nu}$ 3204 (br), 3088, 1595, 1255 cm^{-1}. HRMS (ESI$^+$) calc. for $C_{14}H_{14}N_2O_2$ [M+H]$^+$: 243.1128, found 243.1126. m.p. = 55-56 °C.

(*E*)-1-(3-(bromomethyl)phenyl)-2-(4-methoxyphenyl)diazene (**18**)

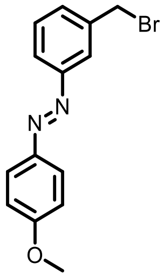

Compound **17** (1.0 g, 4.1 mmol, 1.0 equiv) was dissolved in DCM (50 ml) and the reaction mixture was cooled with an ice-bath. NBS (1.1 g, 6.2 mmol, 1.5 equiv) and PPh_3 (1.5 g, 5.7 mmol, 1.4 equiv) were added at 0 °C and the reaction mixture was allowed to warm up to room temperature. The mixture was stirred for 16 h, followed by removal of solvent under reduced pressure. The crude product was purified by column chromatography (SiO_2; Pentane/EtOAc → 2:1), yielding compound **18** as a dark-red solid (96%, 1.2 g, 4.0 mmol).

^{1}H NMR (400 MHz, $CDCl_3$) δ 7.96 – 7.91 (m, 2H), 7.90 (ddd, J = 2.4, 1.6, 0.9 Hz, 1H), 7.82 (ddd, J = 6.0, 3.2, 2.0 Hz, 1H), 7.50 – 7.46 (m, 2H), 7.05 – 6.99 (m, 2H), 4.58 (d, J = 0.4 Hz, 2H), 3.90 (s, 3H) ppm. ^{13}C NMR (101 MHz, $CDCl_3$) δ 162.1, 153.0, 147.0, 142.0, 129.3, 128.7, 124.8, 122.4, 120.3, 114.2, 65.0, 55.6 ppm. FTIR $\tilde{\nu}$ 3091, 2929, 2836, 1500, 687 cm^{-1}. HRMS (ESI$^+$) calc. for $C_{14}H_{13}BrN_2O$ [M+H]$^+$: 305.0284, found 305.0288. m.p. = 60-62 °C.

(*E*)-(3-((4-methoxyphenyl)diazenyl)phenyl)methanamine (**19**)

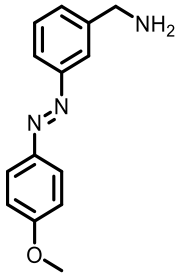

Compound **19** was obtained by reacting compound **18** (0.2 g, 0.7 mmol, 1.0 equiv) in ethanol (12 ml) with an excess of ammonia (25% in water, 4.0 ml) at room temperature for 16 h. The reaction mixture was quenched with water (10 ml) and extracted with ethyl acetate two times (2x20 ml). Then the organic layer was washed with brine, dried with $MgSO_4$ and concentrated under vacuum. Column chromatography (SiO_2; DCM/MeOH → 98:2) was perfumed to obtain the pure product as an orange solid (70%, 0.11 g, 0.46 mmol).

^{1}H NMR (400 MHz, DMSO-d_6) δ 7.94 – 7.85 (m, 2H), 7.84 – 7.80 (m, 1H), 7.68 (dt, J = 6.9, 2.2 Hz, 1H), 7.51 – 7.45 (m, 2H), 7.15 – 7.09 (m, 2H), 3.85 (s, 3H), 3.83 (s, 2H). ^{13}C NMR (101 MHz, DMSO-d_6) δ 162.4, 152.5, 146.7, 138.2, 130.1, 129.5, 124.9, 121.2, 121.0, 115.1, 56.1,

45.6 ppm. FTIR ṽ 3366 (br), 3302, 2923, 2838, 1599, 1249 cm^{-1}. HRMS (ESI^+) calc. for $C_{14}H_{15}N_3O$ $[M+H]^+$: 242.1288, found 242.1287. m.p. = 112-115 °C.

(*E*)-9-isopropyl-*N*-(3-((4-methoxyphenyl)diazenyl)benzyl)-9H-purin-6-amine (**6**)

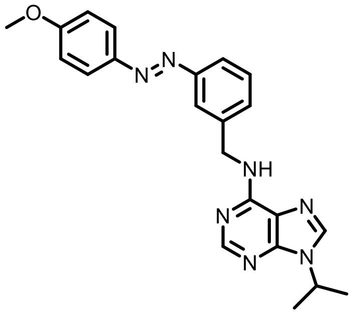

In a microwave vessel, compound **10** (75 mg, 0.38 mmol, 1.0 equiv) was added together with benzylamine **19** (0.11 g, 0.46 mmol, 1.2 equiv) and DIPEA (0.33 ml, 1.9 mmol, 5.0 equiv) in *n*-BuOH (4 ml). The reaction mixture was stirred under microwave irradiation at 150 °C for 45 min. After the reaction was completed (followed by TLC), *n*-BuOH was evaporated and the crude product was directly submitted to column chromatography (SiO_2; DCM/MeOH → 95:5). A mixture of **6** and **10** was obtained. The product was further purified by recrystallization from DCM:Pentane (-20 °C), obtaining an orange solid (57%, 45 mg, 0.11 mmol).

^1H NMR (400 MHz, $CDCl_3$) δ 8.44 (s, 1H), 7.95 – 7.86 (m, 3H), 7.83 – 7.74 (m, 2H), 7.51 – 7.42 (m, 2H), 7.05 – 6.96 (m, 2H), 6.24 (s, 1H), 4.99 (s, 2H), 4.91 – 4.77 (m, 1H), 3.89 (s, 3H), 1.60 (d, J = 6.8 Hz, 6H) ppm. ^{13}C NMR (101 MHz, $CDCl_3$) δ 162.1, 154.7, 153.1, 152.8, 147.0, 139.8, 137.4, 129.6, 129.3, 124.8, 121.7, 121.7, 120.2, 114.2, 77.3, 77.0, 76.7, 55.6, 46.9, 22.7 ppm. Two carbon signals are missing. FTIR ṽ 3260, 3056, 2972, 1621, 1140 cm^{-1}. HRMS (ESI^+) calc. for $C_{22}H_{23}N_7O$ $[M+H]^+$: 402.2037, found 402.2038. m.p. = 152-153 °C.

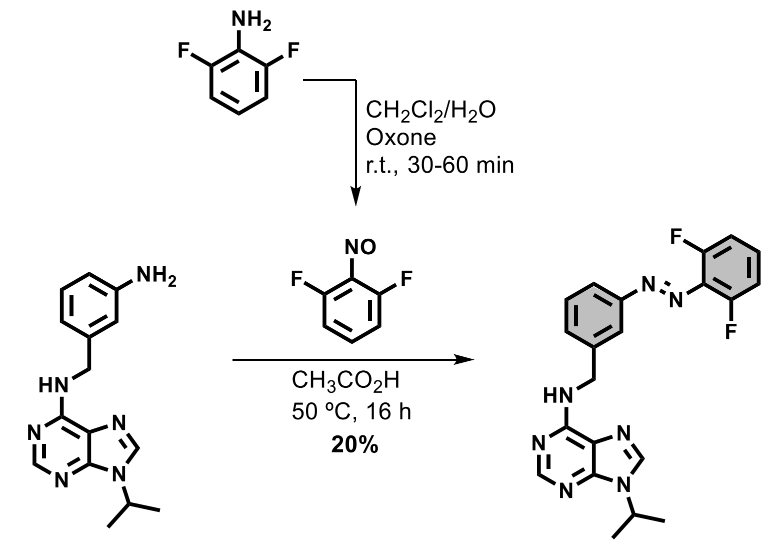

12 **7**

(E)-N-(3-((2,6-difluorophenyl)diazenyl)benzyl)-9-isopropyl-9H-purin-6-amine (**7**)

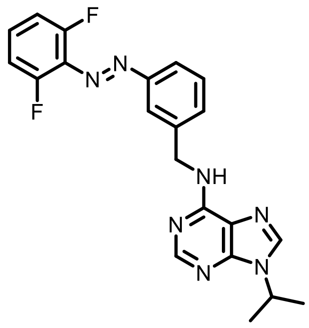

Compound **12** (50 mg, 0.17 mmol, 1.0 equiv) was dissolved in acetic acid (3.0 ml) and 1,3-difluoro-2-nitrosobenzene (38 mg, 0.26 mmol, 1.5 equiv) was added. The reaction mixture was gently heated at 50 °C during 16 h. Acetic acid was evaporated under reduced pressure, the crude was dissolved in ethyl acetate and the organic layer was washed with saturated aq. $NaHCO_3$ solution, water and brine. The organic layer was dried over $MgSO_4$ and the product was purified by column chromatography (SiO_2; DCM/MeOH → 95:5), followed by recrystallization (DCM:Pentane). Compound **7** was obtained in 20% yield as an orange solid (25 mg, 0.06 mmol).

^{1}H NMR (400 MHz, $CDCl_3$) δ 8.41 (s, 1H), 7.94 (d, J = 2.0 Hz, 1H), 7.82 (dt, J = 7.9, 1.5 Hz, 1H), 7.69 (s, 1H), 7.59 – 7.53 (m, 1H), 7.46 (t, J = 7.7 Hz, 1H), 7.30 (tt, J = 8.6, 5.9 Hz, 1H), 7.09 – 6.97 (m, 2H), 6.72 (t, J = 6.1 Hz, 1H), 5.01 (s, 2H), 4.81 (hept, J = 6.7 Hz, 1H), 1.56 (d, J = 6.8 Hz, 6H) ppm. ^{19}F NMR (376 MHz, $CDCl_3$) δ -121.52 – -121.65 (m) ppm. ^{13}C NMR (101 MHz, $CDCl_3$) δ 155.7 (dd, J = 259.1, 4.4 Hz), 154.7, 153.4, 152.8, 140.3, 137.4, 131.1, 130.3 (t, J = 10.3 Hz), 129.4, 122.2, 121.8, 120.1, 113.1 – 111. 8 (m), 46.9, 44.2, 22.7 ppm. Two carbon signals are missing. FTIR $\tilde{\nu}$ 3263, 2978, 1614, 727 cm^{-1}. HRMS (ESI^+) calc. for $C_{21}H_{19}N_7F_2$ $[M+H]^+$: 408.1743, found 408.1739. m.p. = 155-157 °C.

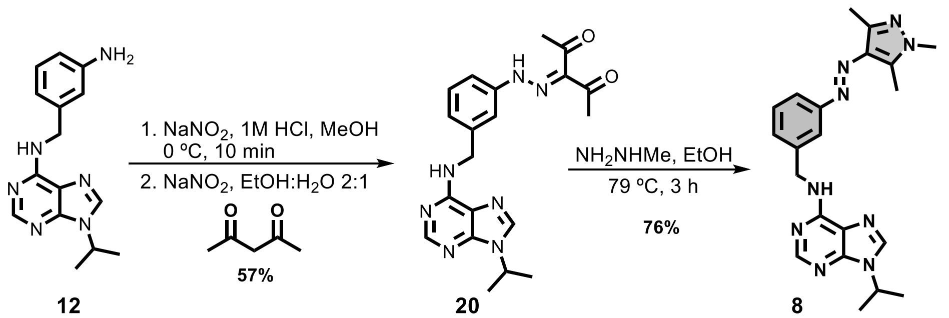

3-(2-(3-(((9-isopropyl-9*H*-purin-6-yl)amino)methyl)phenyl)hydrazineyl)pentane-2,4-dione (**20**)

$NaNO_2$ (89 mg, 1.3 mmol, 1.2 eq.) dissolved in a minimum amount of water was added dropwise to a solution of compound **3** (300 mg, 1.10 mmol, 1.00 eq.) in AcOH (1.5 mL) and 12 M aq. HCl (260 µL) at 0 °C. After stirring for 45 min, the resulting diazonium salt was transferred to a suspension of pentane-2,4-dione (140 µL, 1.4 mmol, 1.3 eq.) and NaOAc (260 mg, 3.2 mmol, 3.0 eq.) in EtOH (1.1 mL) and water (0.7 mL). The mixture was stirred for 1 h and the resulting yellow precipitate was collected *via* vacuum filtration. After washing with water, water/EtOH (1:1) and hexane the yielded solid was dried under vacuum affording the desired compound as a yellow solid (57%, 240 mg, 0.6 mmol). The crude mixture was used in the next step without further purification.

^1H NMR (400 MHz, $CDCl_3$) δ 8.40 (s, 1H), 7.85 (s, 1H), 7.46 (d, *J* = 1.9 Hz, 1H), 7.40 – 7.32 (m, 3H), 7.32 – 7.27 (m, 1H), 7.24 (d, *J* = 7.4 Hz, 1H), 4.98 – 4.81 (m, 3H), 2.58 (s, 3H), 2.40 (s, 3H), 1.61 (d, *J* = 6.8 Hz, 6H) ppm. ^{13}C NMR (101 MHz, $CDCl_3$) δ 197.4, 197.3, 154.4, 152.6, 140.0, 137.7, 134.3, 129.9, 129.1, 126.4, 125.8, 120.3, 116.7, 47.0, 40.9, 31.6, 26.7, 22.7 ppm.

(*E*)-9-isopropyl-*N*-(3-((1,3,5-trimethyl-1*H*-pyrazol-4-yl)diazenyl)benzyl)-9*H*-purin-6-amine (**8**)

Methylhydrazine (42 µL, 0.78 mmol, 1.0 eq.) was added to a solution of compound **20** (280 mg, 0.71 mmol, 1.0 eq.) in EtOH (10 mL) and heated under reflux for 3 h. Concentration under reduced pressure and flash column chromatography (SiO_2; DCM/MeOH → 98:2) yielded **8** as a dark yellow solid (76%, 219 mg, 0.54 mmol).

^1H NMR (400 MHz, $CDCl_3$) δ 8.42 (s, 1H), 7.79 (d, *J* = 1.9 Hz, 1H), 7.76 – 7.63 (m, 2H), 7.44 – 7.36 (m, 2H), 6.68 – 6.60 (m, 1H), 4.96 (s, 2H), 4.81 (hept, *J* = 6.7 Hz, 1H), 3.76 (s, 3H), 2.54 (s, 3H), 2.47 (s, 3H), 1.57 (d, *J* = 6.8 Hz, 7H) ppm. ^{13}C NMR (101 MHz, $CDCl_3$) δ 154.7, 153.9, 152.8, 142.5, 139.6, 138.8, 137.3, 135.1, 129.2, 128.5, 126.1, 121.1, 120.9, 120.0, 119.9, 46.9, 44.5, 36.0, 22.7, 13.8, 10.0 ppm. HRMS (ESI^+) calc. for $C_{21}H_{19}N_7F_2$ $[M+H]^+$: 404.2306, found 404.2309.

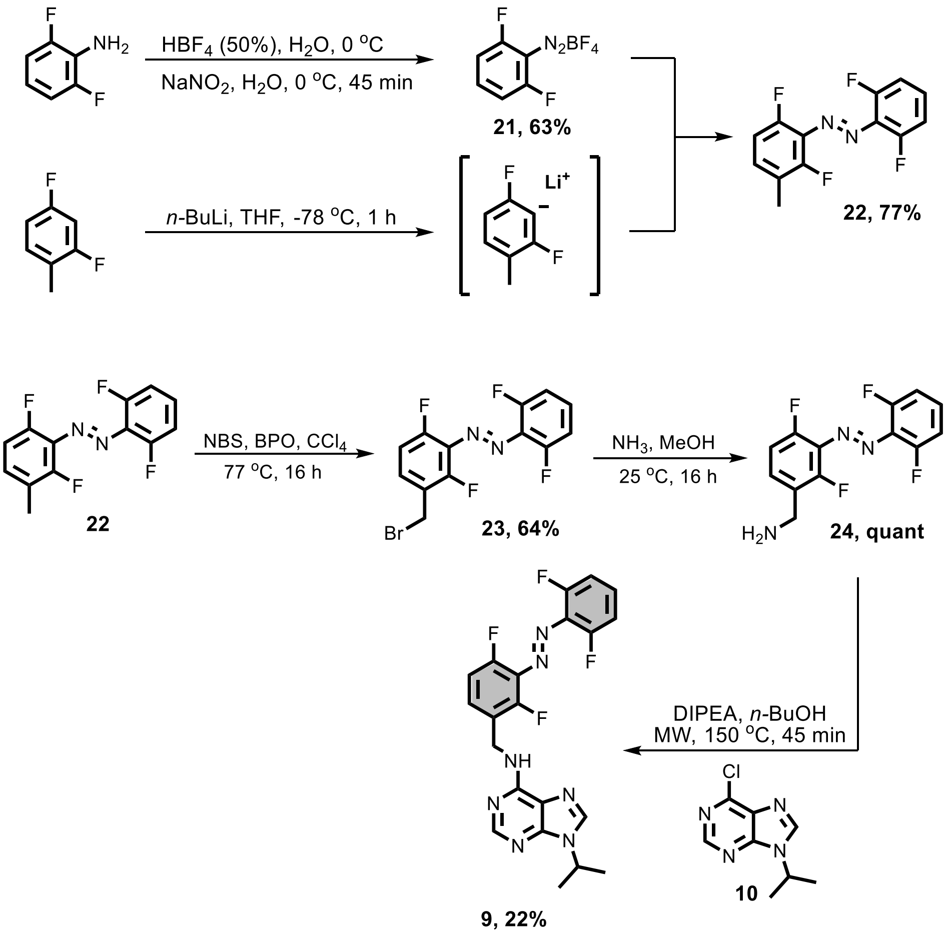

2,6-difluorobenzenediazonium (**21**)

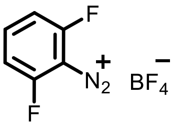

2,6-Difluoroaniline (2.0 g, 16 mmol, 1.0 equiv) and HBF_4 (50%, 5.2 ml, 42 mmol, 2.7 equiv) were dissolved in water (6 ml) and cooled down in an ice bath. Subsequently, $NaNO_2$ (1.1 g, 16 mmol, 1.0 equiv) in water (3 ml) was added dropwise to the reaction and the reaction mixture was stirred for 45 min at 0 °C. Afterwards, the reaction mixture was filtrated over a glass filter and the crystals were washed with cold diethyl ether and dried under vacuum (63%, 2.2 g, 9.7 mmol). The product was used immediately, but if needed it could be stored at room temperature in the dark under N_2 atmosphere to prevent degradation.

^{1}H NMR (400 MHz, DMSO-d_6) δ 8.58 – 8.45 (m, 1H), 7.99 – 7.86 (m, 2H). As in literature.[3]

(*E*)-1-(2,6-difluoro-3-methylphenyl)-2-(2,6-difluorophenyl)diazene (**22**)

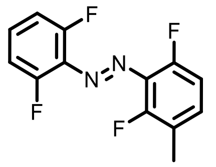

2,4-difluoro-1-methylbenzene (1.0 ml, 8.8 mmol, 1.0 equiv) was dissolved in dry THF (15 ml). The mixture was cooled down to -78 °C and slow addition of *n*-BuLi (1.6 M in hexane, 5.5 ml, 8.8 mmol, 1.0 equiv) was performed. The reaction mixture was stirred for 1 h at the same temperature and, subsequently, compound **21** (2.0 g, 8.8 mmol, 1.0 equiv) was added in one portion to the reaction mixture. Then, the solution was allowed to warm up to room temperature over 1.5 h, after which it was quenched by the addition of aq. $NaHCO_3$ solution. The aqueous phase was extracted with EtOAc, and the combined organic layers were washed with brine, dried over anhydrous $MgSO_4$ and concentrated under reduced pressure. Purification was performed using column chromatography (SiO_2, Pentane/EtOAc 98:2) obtaining **22** as a dark red solid (77%, 1.8 g, 6.7 mmol).

^{1}H NMR (400 MHz, DMSO-*d₆*) δ 7.66 – 7.57 (m, 1H), 7.51 (q, J = 7.8 Hz, 1H), 7.39 – 7.31 (m, 2H), 7.29 – 7.22 (m, 1H), 2.28 (s, 3H) ppm. ^{13}C NMR (101 MHz, DMSO-d_6) δ 156.3 (d, J = 4.1 Hz), 154.48 – 154.26 (m), 153.8 (d, J = 4.1 Hz), 151.8 (d, J = 3.7 Hz), 134.4 (dd, J = 10.0, 7.1 Hz), 133.5 (t, J = 10.6 Hz), 113.8 – 113.7 (m), 113.6 (d, J = 3.0 Hz), 112.9 (d, J = 4.0 Hz), 112.7 (d, J = 4.1 Hz), 14.1 (d, J = 3.8 Hz) ppm. HRMS (ESI$^+$) for $C_{13}H_8F_4N_2$: calculated 269.0696, measured 269.0697.

(*E*)-1-(3-(bromomethyl)-2,6-difluorophenyl)-2-(2,6-difluorophenyl)diazene (**23**)

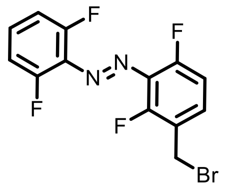

In a Schlenk flask under inert atmosphere, compound **22** (0.50 g, 1.9 mmol, 1.0 equiv) was dissolved in tetrachloromethane (15 ml). *N*-bromosuccinimide (0.4 g, 2.1 mmol, 1.1 equiv) was subsequently added, together with the radical initiator benzoyl peroxide (BPO, 10 mol%, 55 mg, 0.2 mmol). The reaction mixture was heated up to 85 °C and it was stirred for 16 h until completion was observed. The solvent was removed under reduced pressure and the crude mixture was redissolved in EtOAc. The organic phase was washed with aq. solution of $NaHCO_3$ and brine, dried over $MgSO_4$, filtered and evaporated under vacuum. The crude was purified by flash column chromatography (SiO_2, Pentane/EA 100:0 → 95:5) obtaining product **23** as a dark red solid (64%, 0.4 g, 1.2 mmol). Due to presence of inseparable starting material (**22**), the obtained mixture was used in the next step without complete characterisation.

^1H NMR (400 MHz, $CDCl_3$) δ 7.60 – 7.32 (m, 2H), 7.07 (td, *J* = 10.2, 9.6, 3.8 Hz, 3H), 4.55 (s, 2H) ppm. ^{13}C NMR (101 MHz, $CDCl_3$) δ 157.0 (d, *J* = 4.1 Hz), 156.6, 154.7, 132.5 (dd, *J* = 10.3, 5.1 Hz), 131.8 (t, *J* = 10.5 Hz), 112.9 (d, *J* = 4.4 Hz), 112.8, 112.8 – 112.7 (m), 112.7, 112.6 – 112.5 (m), 24.4 (d, *J* = 5.2 Hz) ppm.

(*E*)-(3-((2,6-difluorophenyl)diazenyl)-2,4-difluorophenyl)methanamine (**24**)

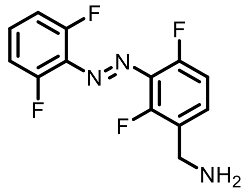

Compound **23** (440 mg, 1.26 mmol, 1.00 equiv) was added in an 7 N ammonia solution in methanol (6.0 ml) and the reaction mixture was additionally diluted with methanol (3.0 ml). The turbid solution was stirred at room temperature over 16 h. After this time, the solution became clear. The solvent was removed under reduced pressure and the crude product was used in the next step without further purification (quant, 0.35 g, 1.2 mmol).

^1H NMR (400 MHz, DMSO-d_6) *δ* 8.22 (s, 2H), 7.75 (td, *J* = 8.3, 5.8 Hz, 1H), 7.65 (tt, *J* = 8.5, 6.1 Hz, 1H), 7.48 (ddd, *J* = 10.4, 8.8, 1.5 Hz, 1H), 7.42 – 7.32 (m, 2H), 4.15 (s, 2H) ppm.

(*E*)-*N*-(3-((2,6-difluorophenyl)diazenyl)-2,4-difluorobenzyl)-9-isopropyl-9*H*-purin-6-amine (**9**)

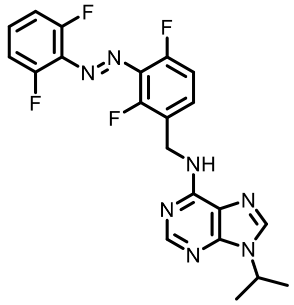

Compound **24** (500 mg, 1.77 mmol, 1.20 equiv), compound **10** (287 mg, 1.46 mmol, 1.00 equiv) and DIPEA (1.00 ml, 6.10 mmol, 5.00 equiv) were dissolved in *n*-BuOH (15 ml) in a microwave vessel. The mixture was stirred under microwave irradiation at 150 °C for 45 min. After the reaction was completed (followed by TLC), *n*-BuOH was evaporated and the crude product was directly submitted to column chromatography (SiO_2; DCM/MeOH 95:5). Subsequent recrystallization (EtOAc:Pentane) yielded **9** as a red solid (22%, 139 mg, 0.31 mmol).

^1H NMR (400 MHz, $CDCl_3$) δ 8.40 (s, 1H), 7.80 (s, 1H), 7.54 (td, *J* = 8.3, 5.8 Hz, 1H), 7.36 (tt, *J* = 8.2, 5.8 Hz, 1H), 7.11 – 7.00 (m, 2H), 6.98 (ddd, *J* = 10.3, 8.8, 1.7 Hz, 1H), 6.52 (s, 1H), 4.99 (s, 2H), 4.83 (hept, *J* = 6.8 Hz, 1H), 1.59 (d, *J* = 6.8 Hz, 7H) ppm. ^{13}C NMR (101 MHz, $CDCl_3$) δ 156.89 (d, *J* = 4.1 Hz), 155.98 (d, *J* = 4.0 Hz), 154.78 (d, *J* = 4.2 Hz), 154.48, 154.29 (d, *J* = 4.1 Hz), 153.38 (d, *J* = 4.0 Hz), 152.57, 152.18 (d, *J* = 4.2 Hz), 137.62, 131.78 – 131.56 (m), 131.44 (d, *J* = 10.3 Hz), 123.25 (dd, *J* = 14.0, 4.0 Hz), 120.16, 112.84 – 112.40 (m), 112.20 (dd, *J* = 20.2, 4.1 Hz), 47.03, 37.84, 22.66 ppm. ^{19}F NMR (376 MHz, $CDCl_3$) δ -121.16 (dd, *J* = 9.1, 5.8 Hz), -122.21 (dd, *J* = 10.3, 5.9 Hz), -126.13 (d, *J* = 8.0 Hz) ppm. FTIR $\tilde{\nu}$ 3272, 2979, 1612, 1473,

1224, 1020, 786, 648 cm^{-1}. HRMS (ESI^+) calc. for $C_{21}H_{17}N_7F_4$ $[M+H]^+$: 444.1554, found 444.1556. m.p. = 60-63 °C.

Photostationary state (PSS) distribution determination

Compound **3**

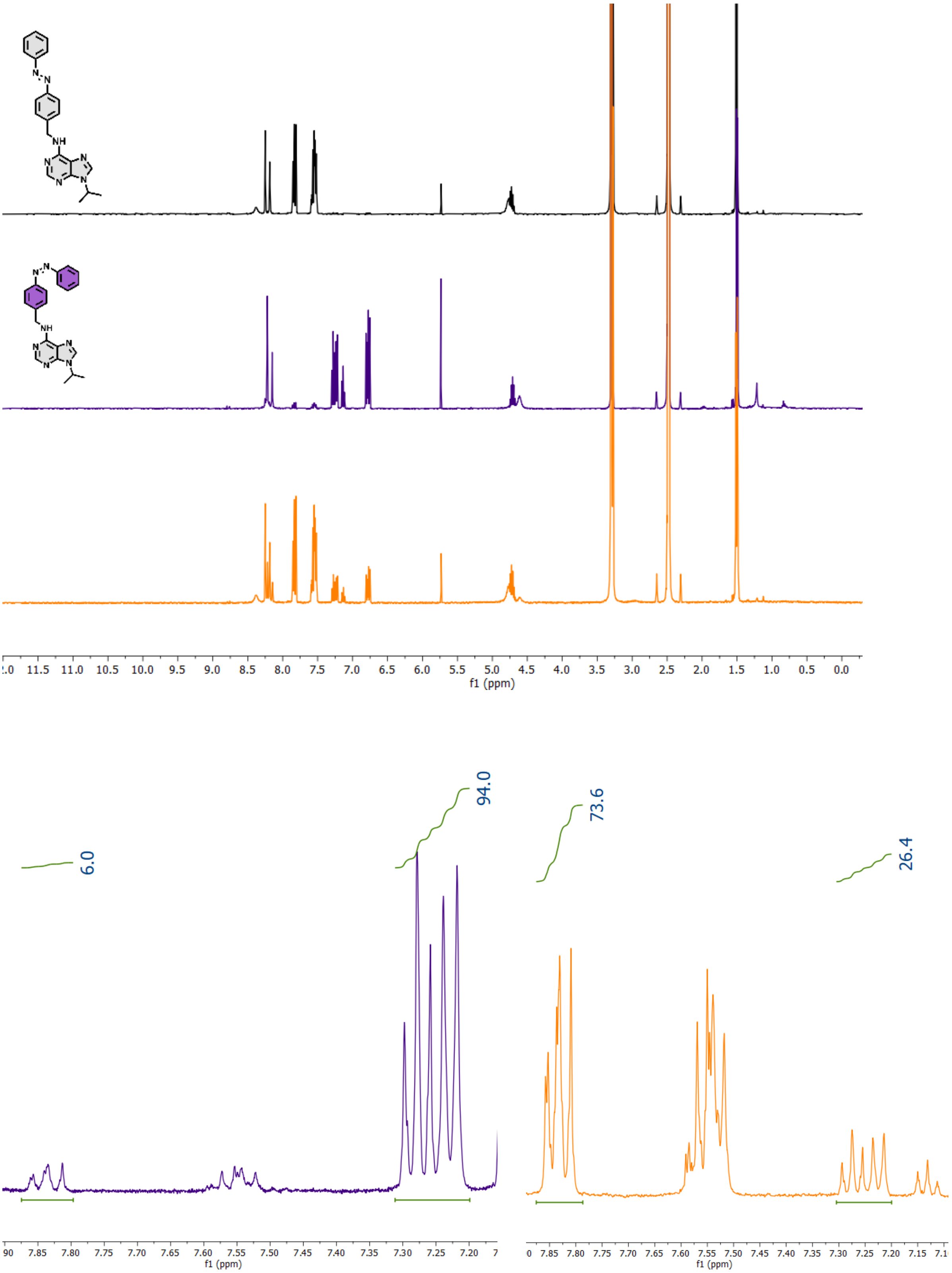

Supplementary Figure 1. PSS determination for compound **3**. NMR spectra before (black), after UV light (λ_{max} = 365 nm) (purple), and after white light (orange) irradiation of compound **3** until PSS was reached.

Compound **4**

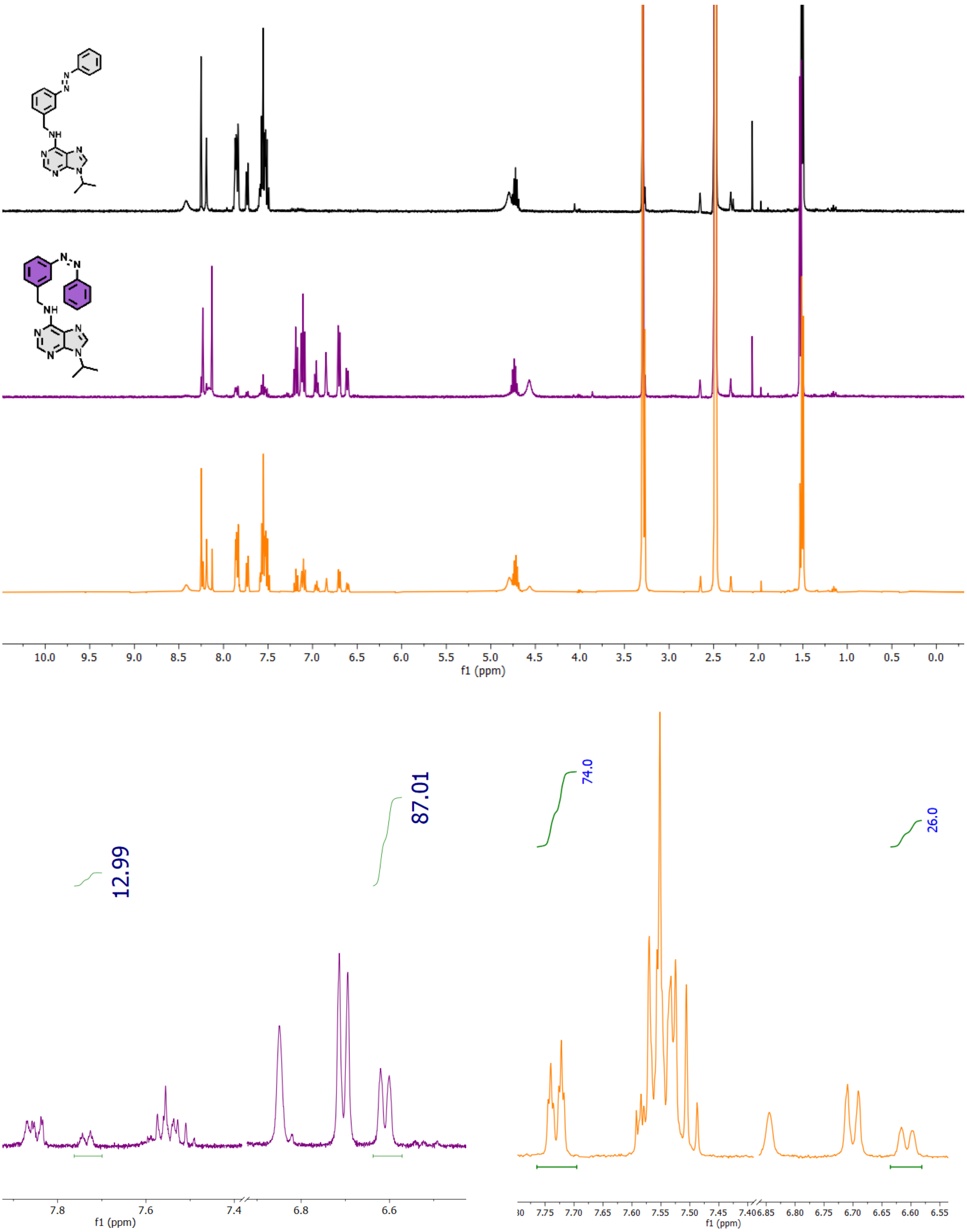

Supplementary Figure 2. PSS determination for compound **4**. NMR spectra before (black), after UV light (λ_{max} = 365 nm) (purple), and after white light (orange) irradiation of compound **4** until PSS was reached.

Compound **5**

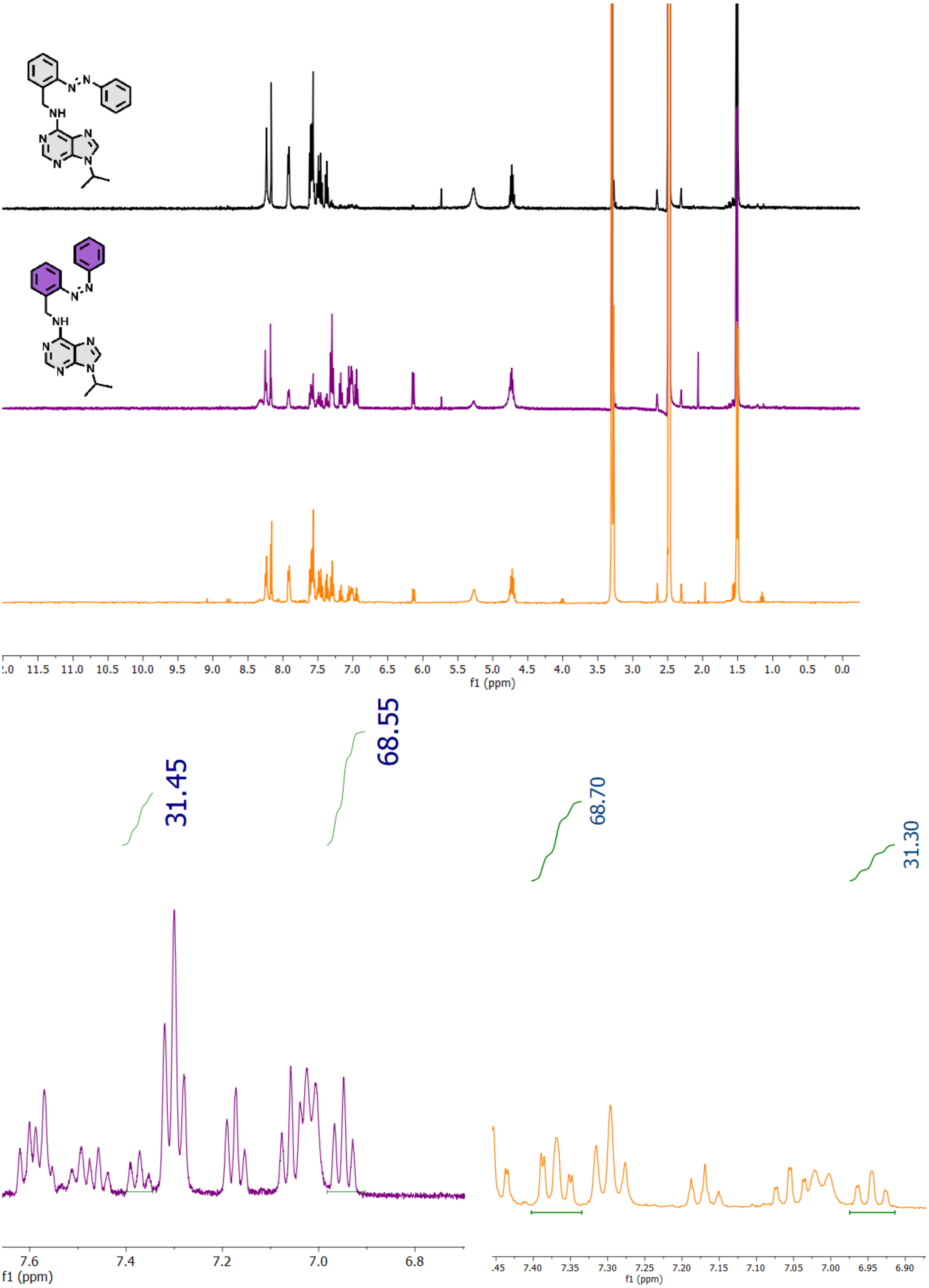

Supplementary Figure 3. PSS determination for compound **5**. NMR spectra before (black), after UV light (λ_{max} = 365 nm) (purple), and after white light (orange) irradiation of compound **5** until PSS was reached.

Compound **6**

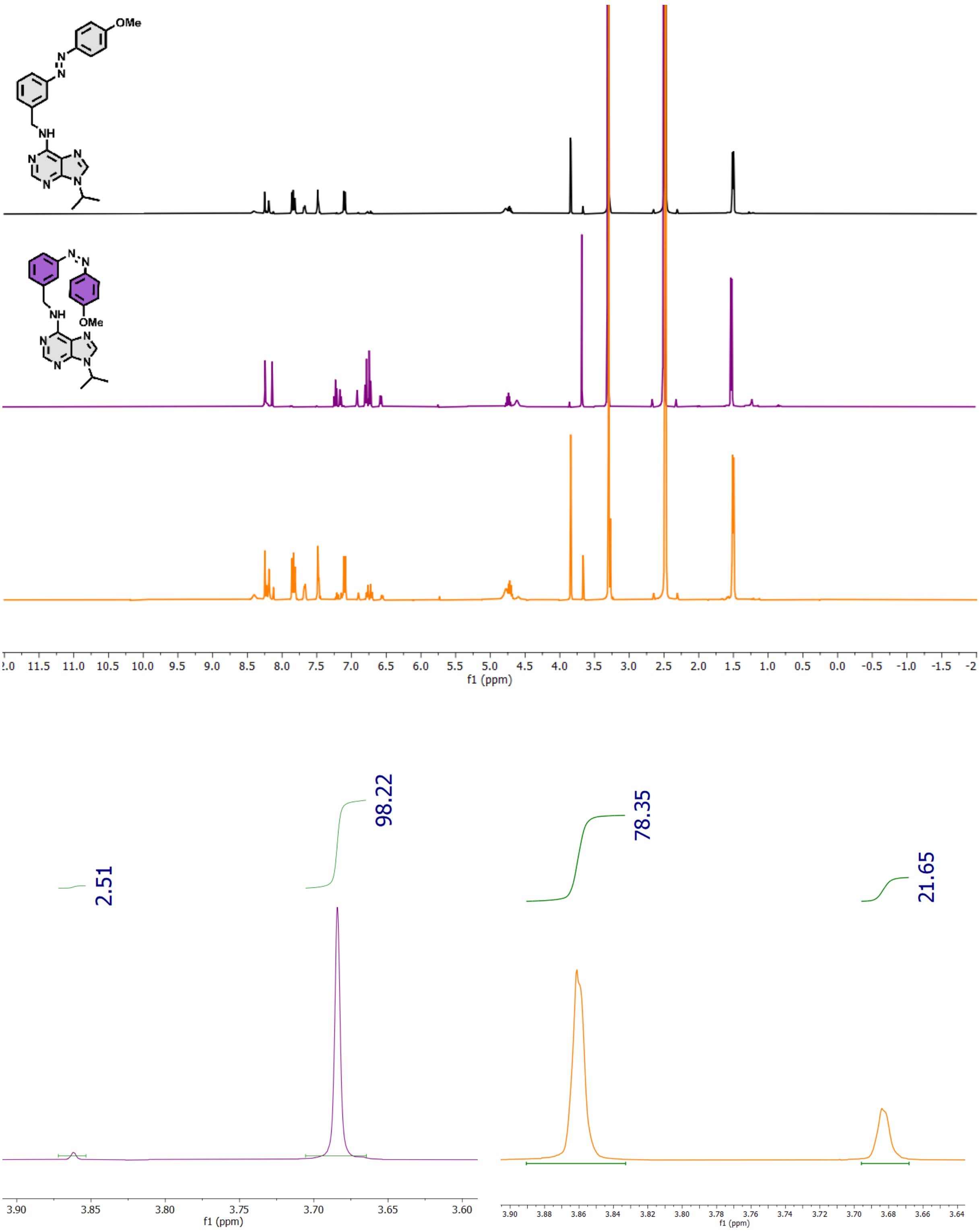

Supplementary Figure 4. PSS determination for compound **6**. NMR spectra before (black), after UV light (λ_{max} = 365 nm) (purple), and after white light (orange) irradiation of compound **6** until PSS was reached.

Compound **7**

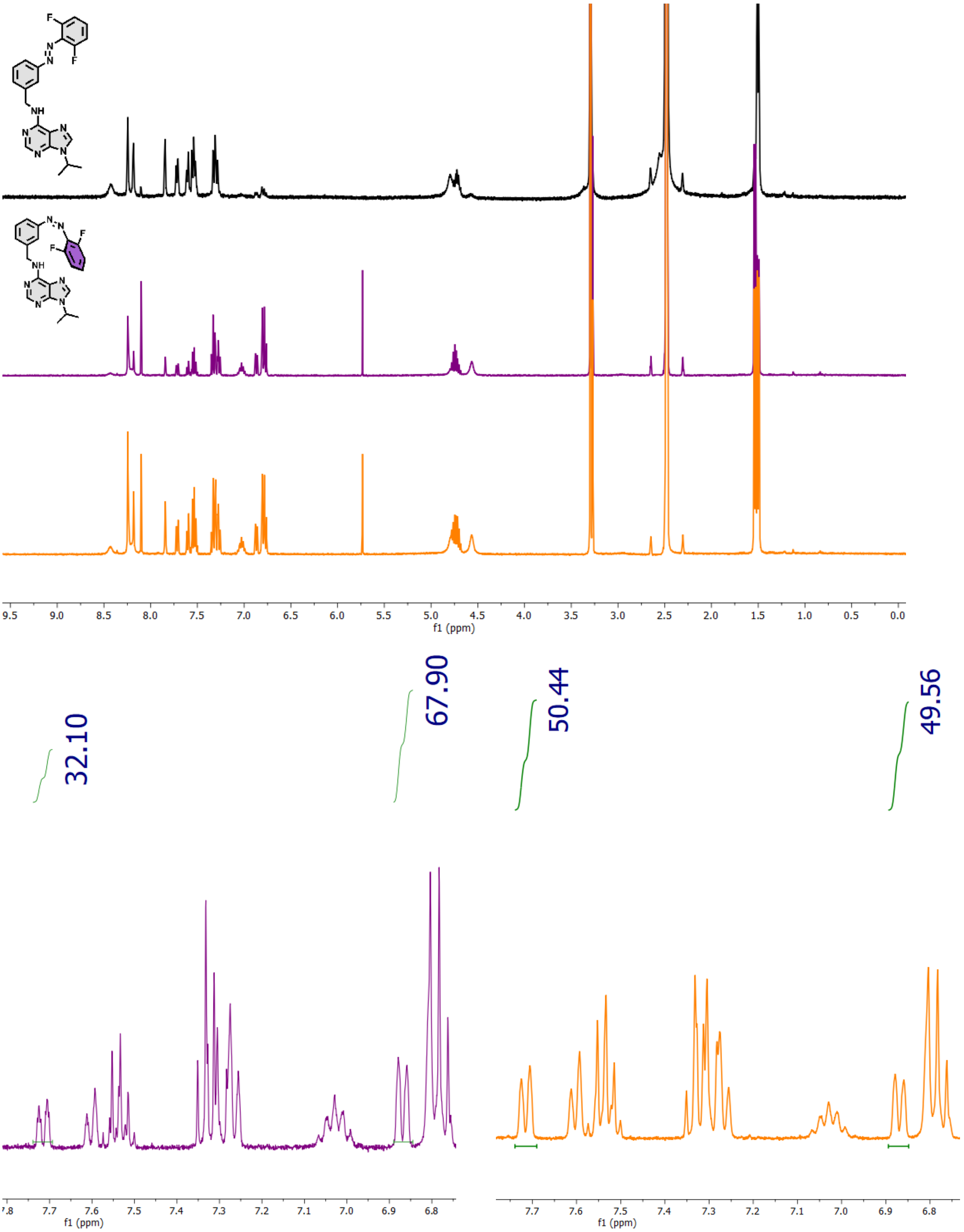

Supplementary Figure 5. PSS determination for compound **7**. NMR spectra before (black), after UV light (λ_{max} = 365 nm) (purple), and after white light (orange) irradiation of compound **7** until PSS was reached.

Compound **8**

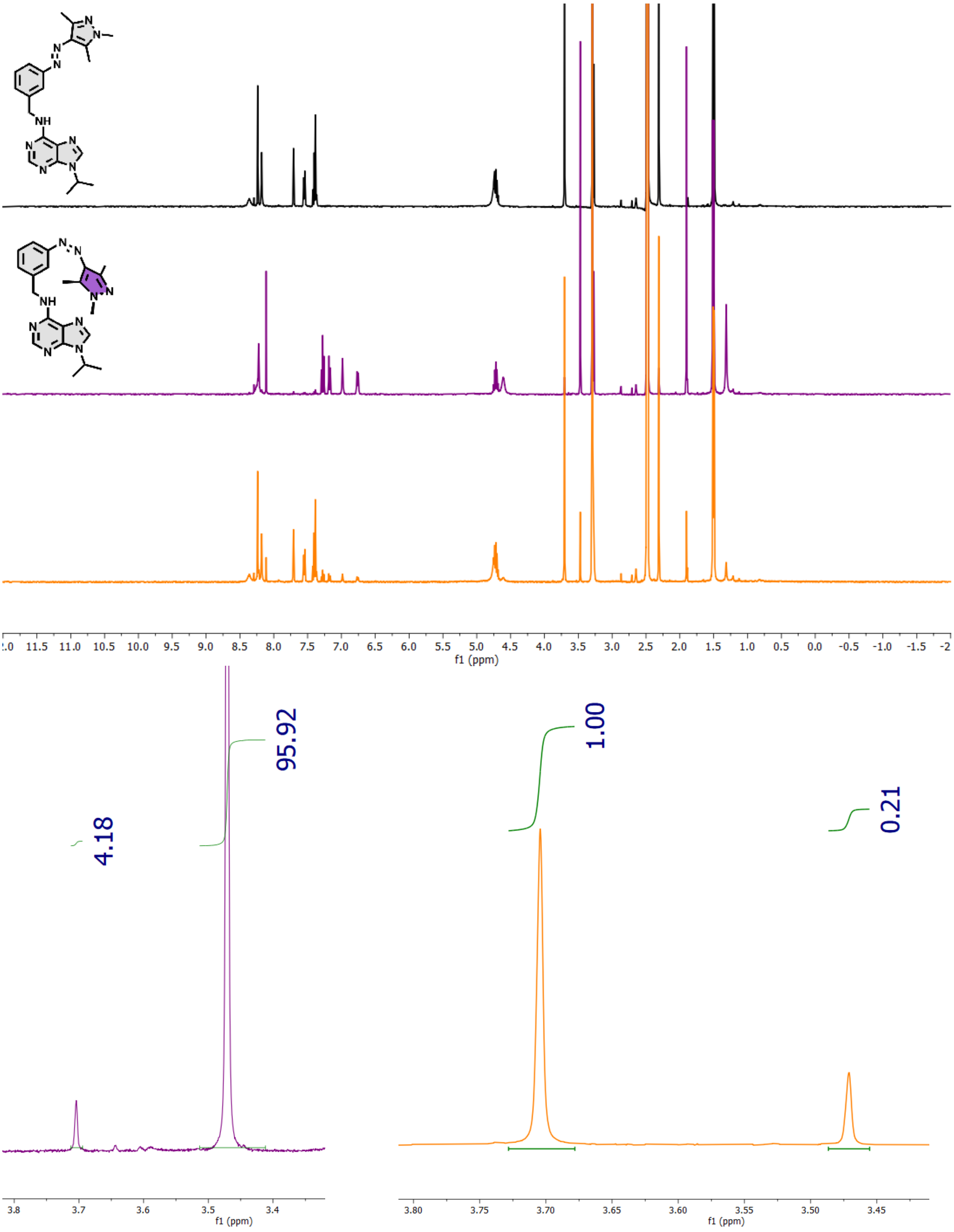

Supplementary Figure 6. PSS determination for compound **8**. NMR spectra before (black), after UV light (λ_{max} = 365 nm) (purple), and after white light (orange) irradiation of compound **8** until PSS was reached.

Compound **9**

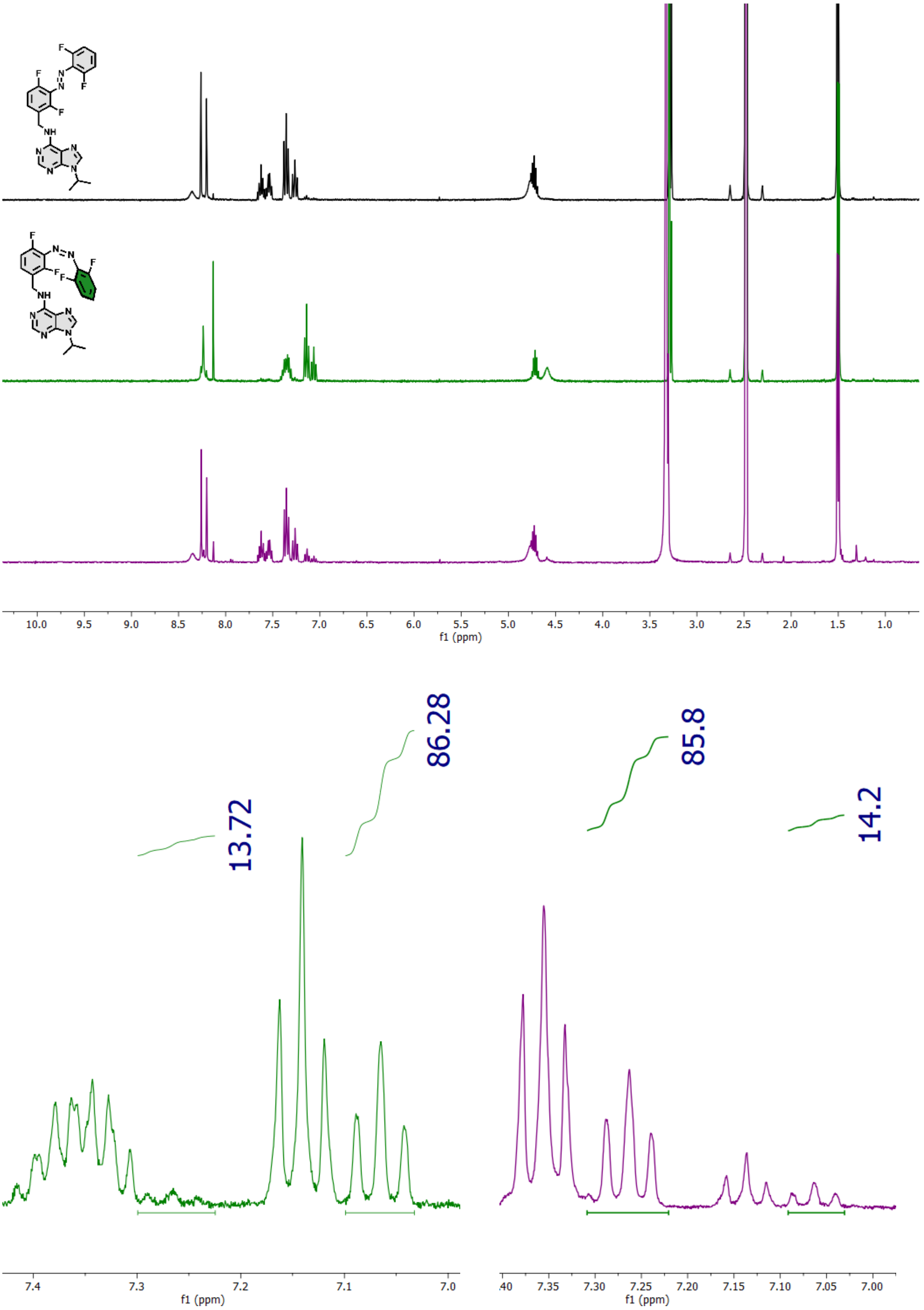

Supplementary Figure 7. PSS determination for compound **9**. NMR spectra before (black), after green light (λ_{max} = 530 nm) (green), and after violet light (λ_{max} = 400 nm) (purple) irradiation of compound **9** until PSS was reached.

LCMS traces of stability studies

Compound **3**

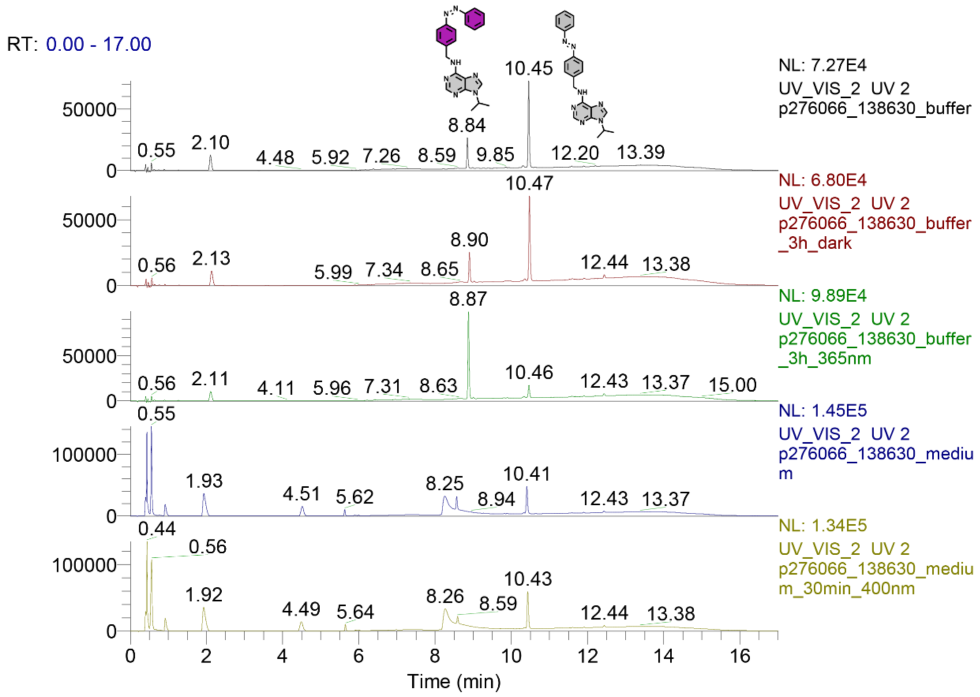

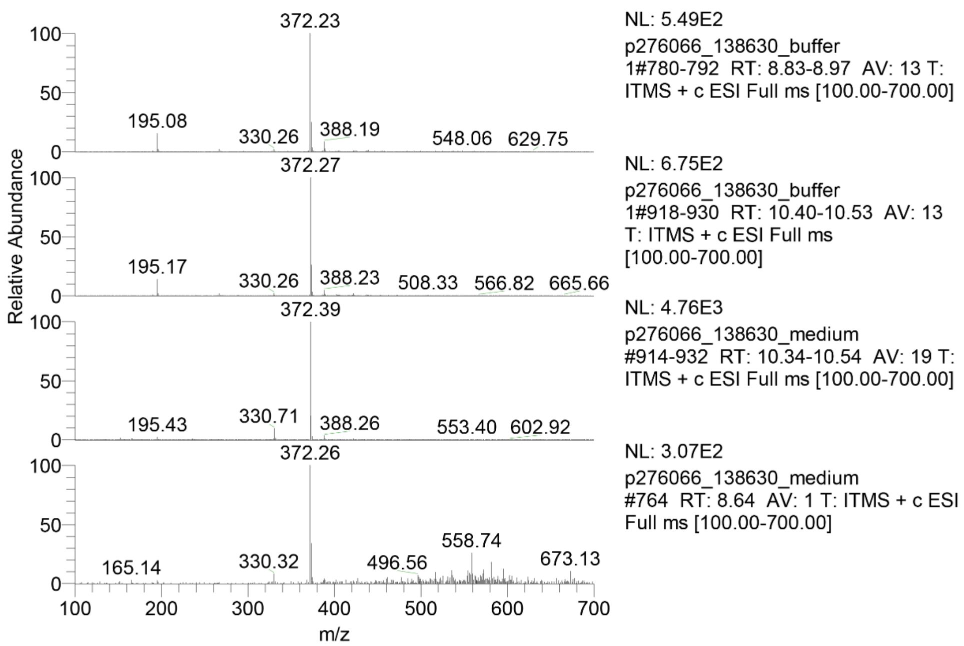

Supplementary Figure 8. Chemical stability of compound **3** under dark and irradiation conditions followed by LCMS. All the samples were prepared as 40 µM solution in buffer or cell culture medium. 'Buffer' and 'medium' samples were injected immediately upon dissolving compound **3**, 'buffer_3h_dark' was injected after 3 h of keeping the solution in dark, 'buffer_3h_365nm' after irradiation of the sample for 3 h with UV light in kinase assay buffer, and 'medium_30min_400nm' after 30 min of irradiation with 400 nm in cell culture medium. The *trans*-isomer was followed at the retention time of 10.41-10.47 min and the *cis*-isomer at the retention time of 8.84-8.90 min. Mass spectra of both isomers in different media are also shown. Products of reduction or oxidation were not observed.

Compound **4**

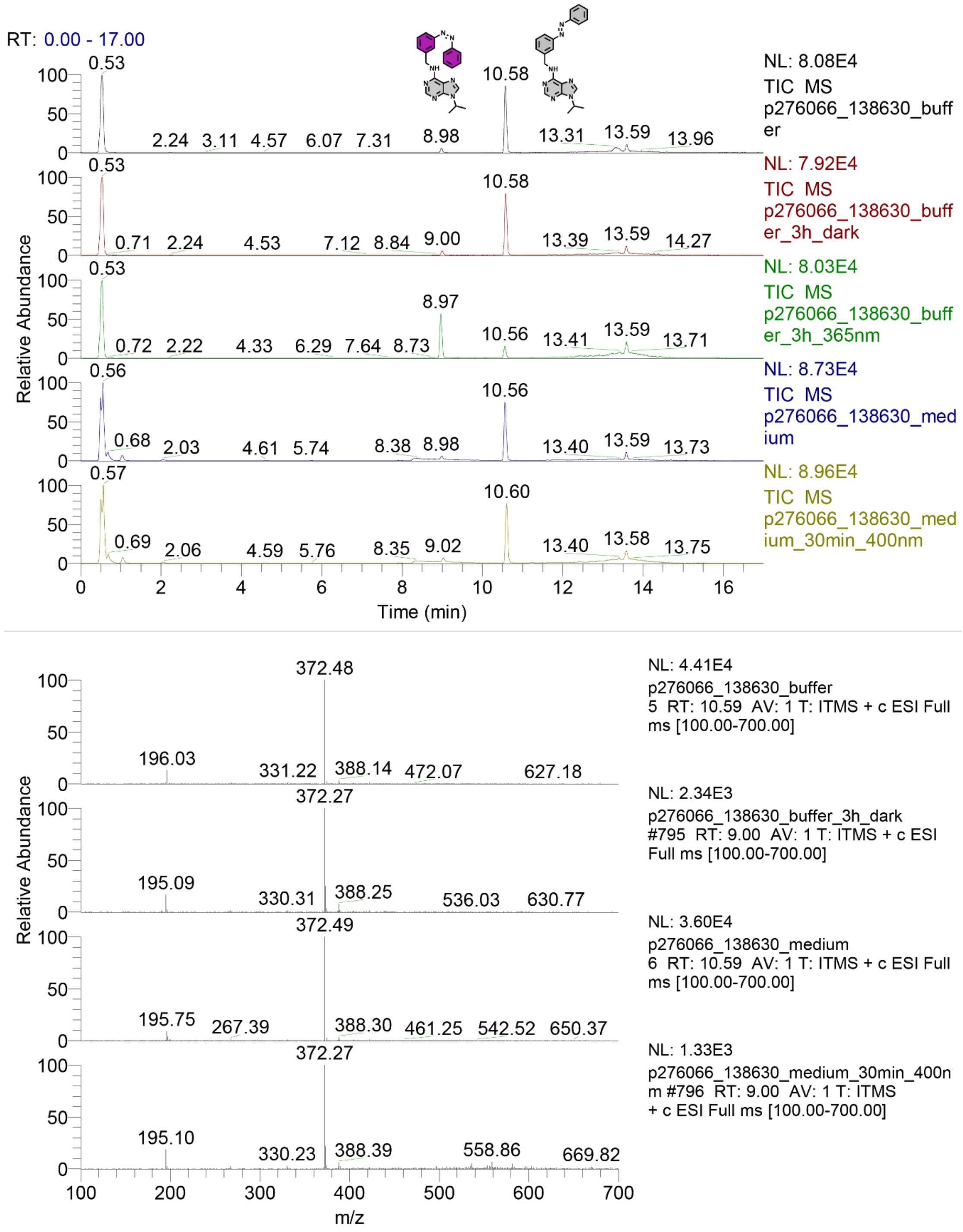

Supplementary Figure 9. Chemical stability of compound **4** under dark and irradiation conditions followed by LCMS. All the samples were prepared as 40 µM solution in kinase assay buffer or cell culture medium. 'Buffer' and 'medium' samples were injected immediately upon dissolving compound **4**, 'buffer_3h_dark' was injected after 3 h of keeping the solution in dark, 'buffer_3h_365nm' after irradiation of the sample for 3 h with UV light in kinase assay buffer, and 'medium_30min_400nm' after 30 min of irradiation with 400 nm in cell culture medium. The *trans*-isomer was followed at the retention time of 10.56-10.60 min and the *cis*-isomer at the retention time of 8.97-9.02 min. Mass spectra of both isomers in different media are also shown. Products of reduction or oxidation were not observed.

Compound **5**

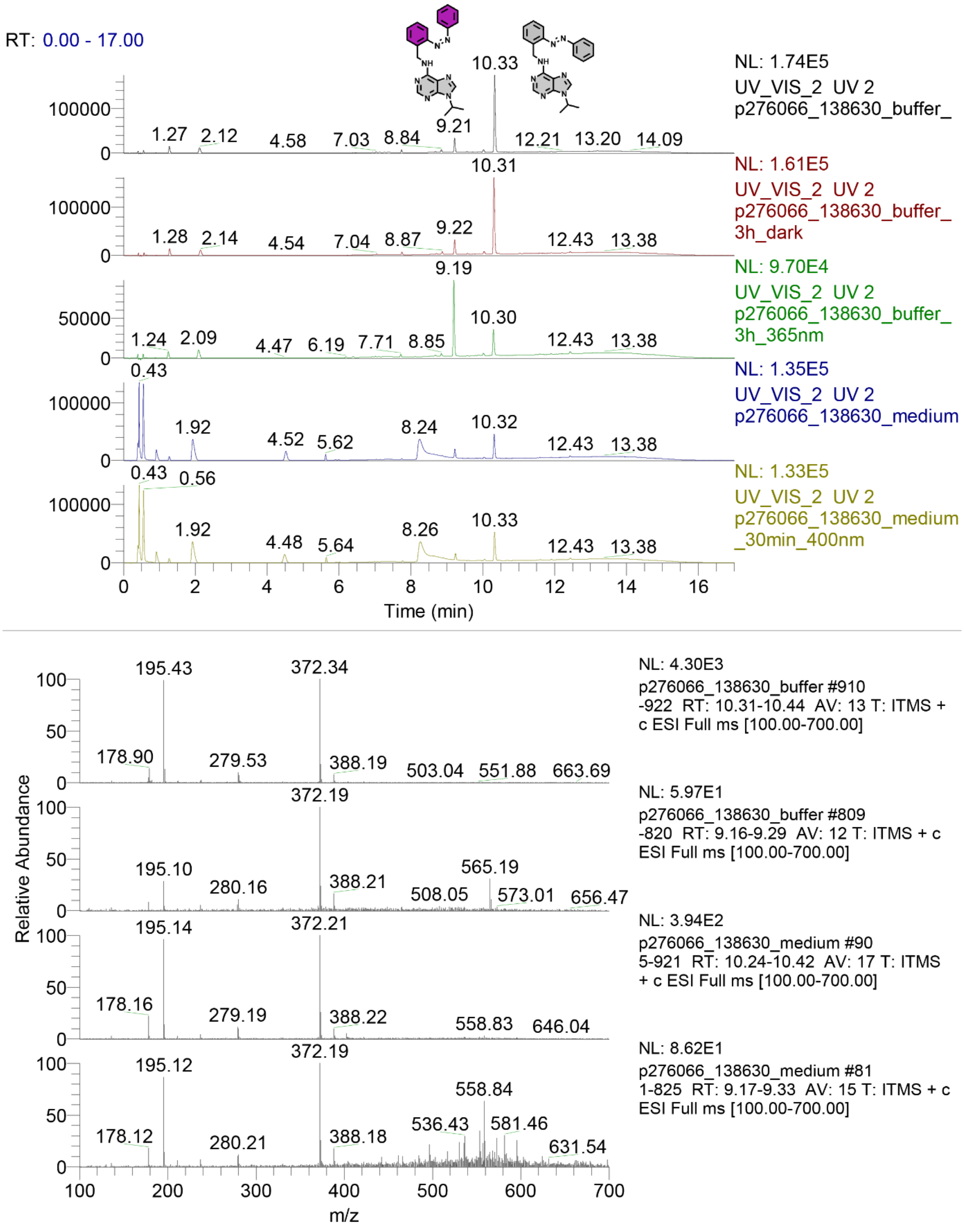

Supplementary Figure 10. Chemical stability of compound **5** under dark and irradiation conditions followed by LCMS. All the samples were prepared as 40 µM solution in kinase assay buffer or cell culture medium. 'Buffer' and 'medium' samples were injected immediately upon dissolving compound **5**, 'buffer_3h_dark' was injected after 3 h of keeping the solution in dark, 'buffer_3h_365nm' after irradiation of the sample for 3 h with UV light in kinase assay buffer, and 'medium_30min_400nm' after 30 min of irradiation with 400 nm in cell culture medium. The *trans*-isomer was followed at the retention time of 10.30-10.33 min and the *cis*-isomer at the retention time of 9.19-9.21 min. Mass spectra of both isomers in different media are also shown. Products of reduction or oxidation were not observed.

Compound **6**

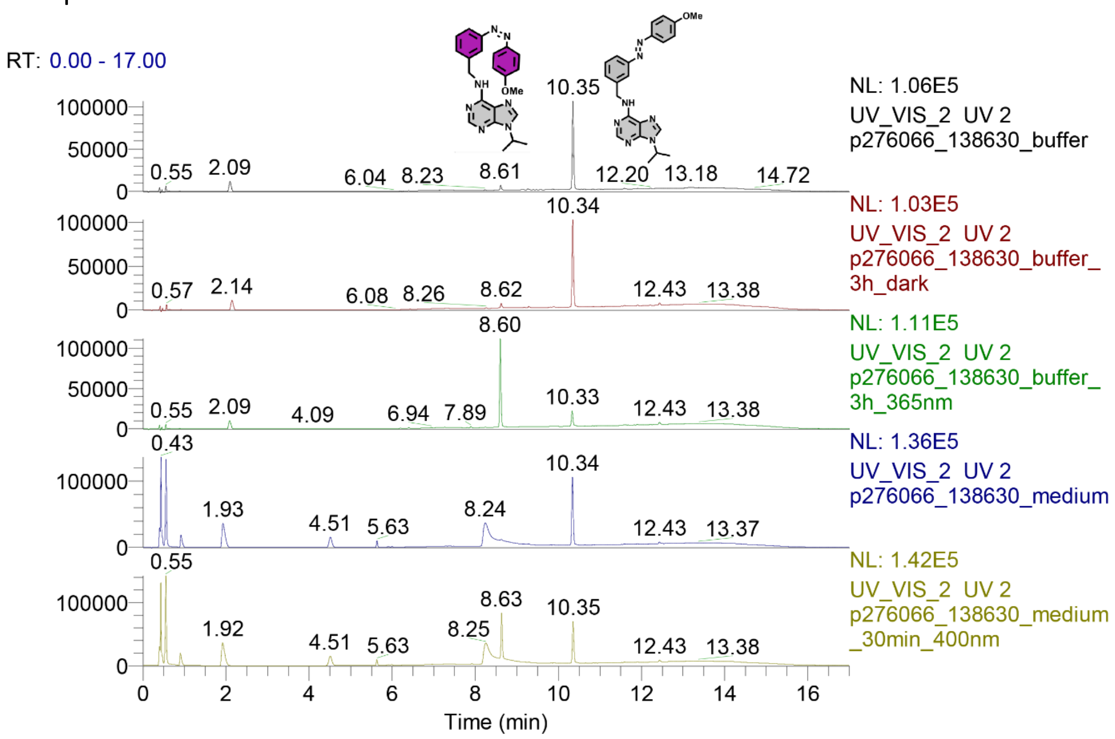

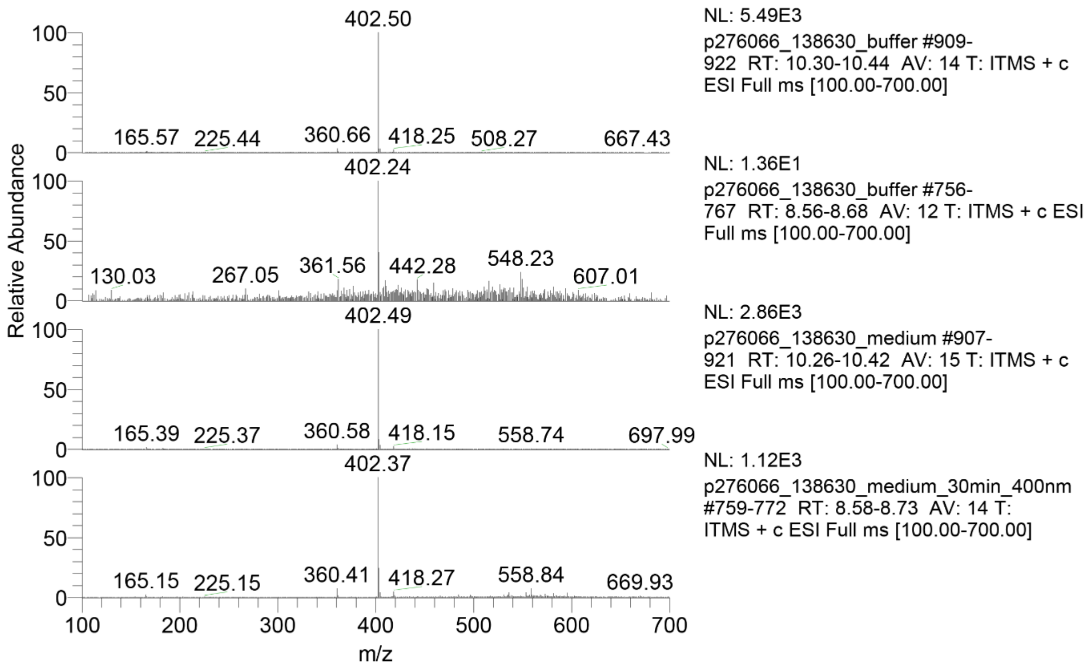

Supplementary Figure 11. Chemical stability of compound **6** under dark and irradiation conditions followed by LCMS. All the samples were prepared as 40 µM solution in kinase assay buffer or cell culture medium. 'Buffer' and 'medium' samples were injected immediately upon dissolving compound **6**, 'buffer_3h_dark' was injected after 3 h of keeping the solution in dark, 'buffer_3h_365nm' after irradiation of the sample for 3 h with UV light in kinase assay buffer, and 'medium_30min_400nm' after 30 min of irradiation with 400 nm in cell culture medium. The *trans*-isomer was followed at the retention time of 10.33-10.35 min and the *cis*-isomer at the retention time of 8.60-8.63 min. Mass spectra of both isomers in different media are also shown. Products of reduction or oxidation were not observed.

Compound **7**

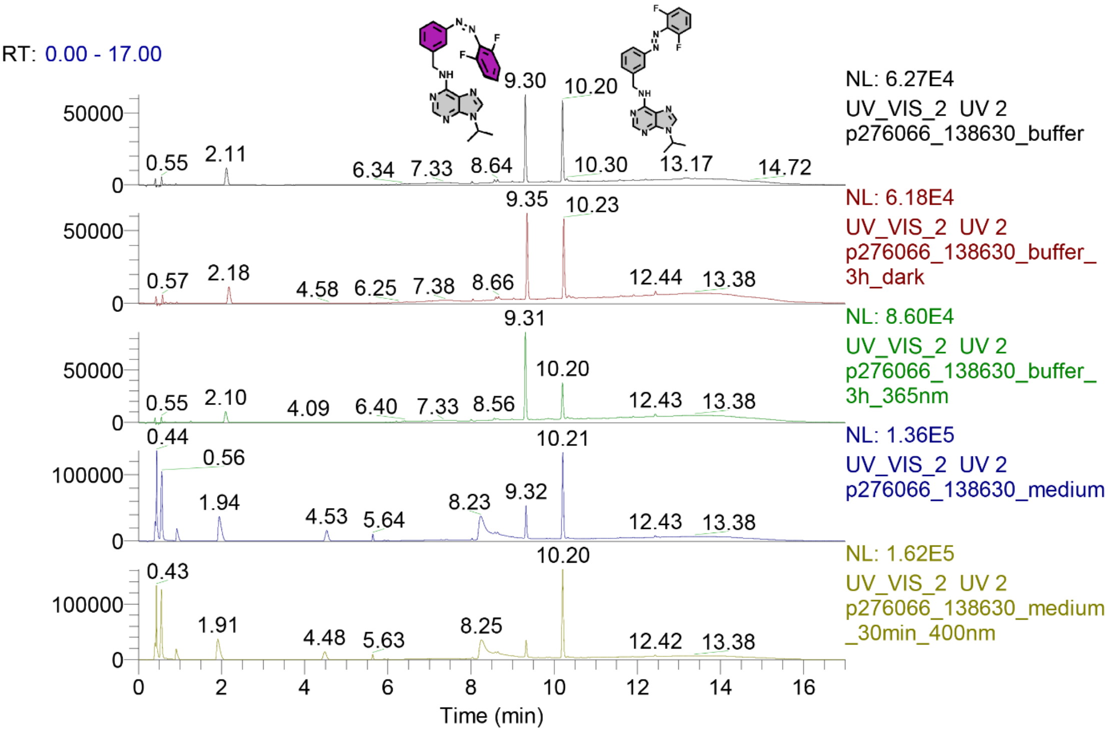

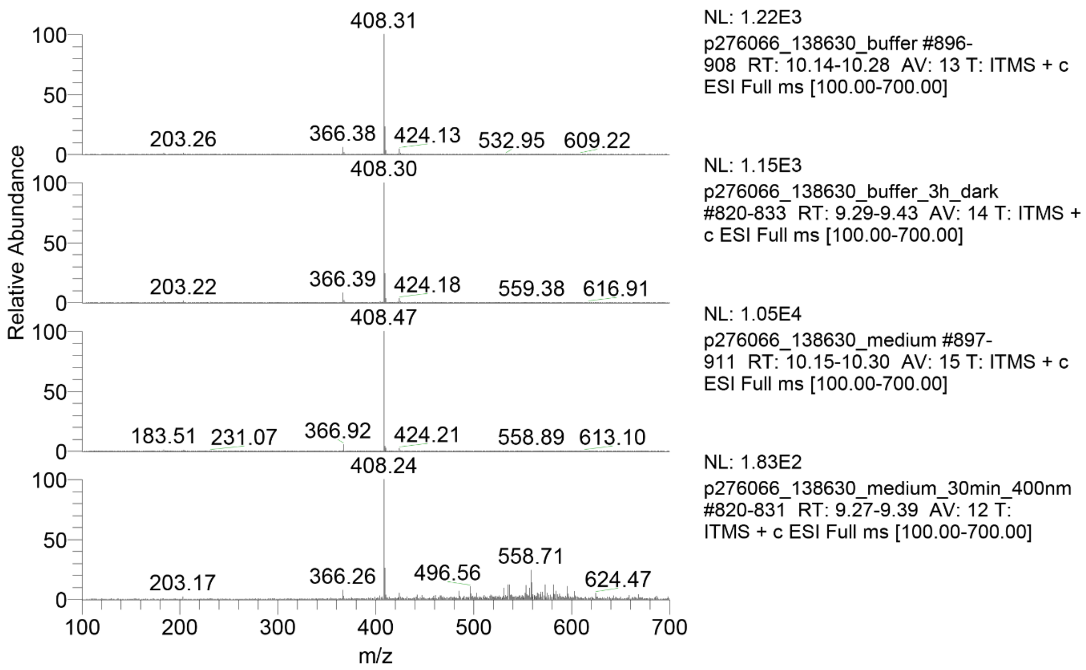

Supplementary Figure 12. Chemical stability of compound **7** under dark and irradiation conditions followed by LCMS. All the samples were prepared as 40 µM solution in kinase assay buffer or cell culture medium. 'Buffer' and 'medium' samples were injected immediately upon dissolving compound **7**, 'buffer_3h_dark' was injected after 3 h of keeping the solution in dark, 'buffer_3h_365nm' after irradiation of the sample for 3 h with UV light in kinase assay buffer, and 'medium_30min_400nm' after 30 min of irradiation with 400 nm in cell culture medium. The *trans*-isomer was followed at the retention time of 10.20-10.23 min and the *cis*-isomer at the retention time of 9.30-9.35 min. Mass spectra of both isomers in different media are also shown. Products of reduction or oxidation were not observed.

Compound **8**

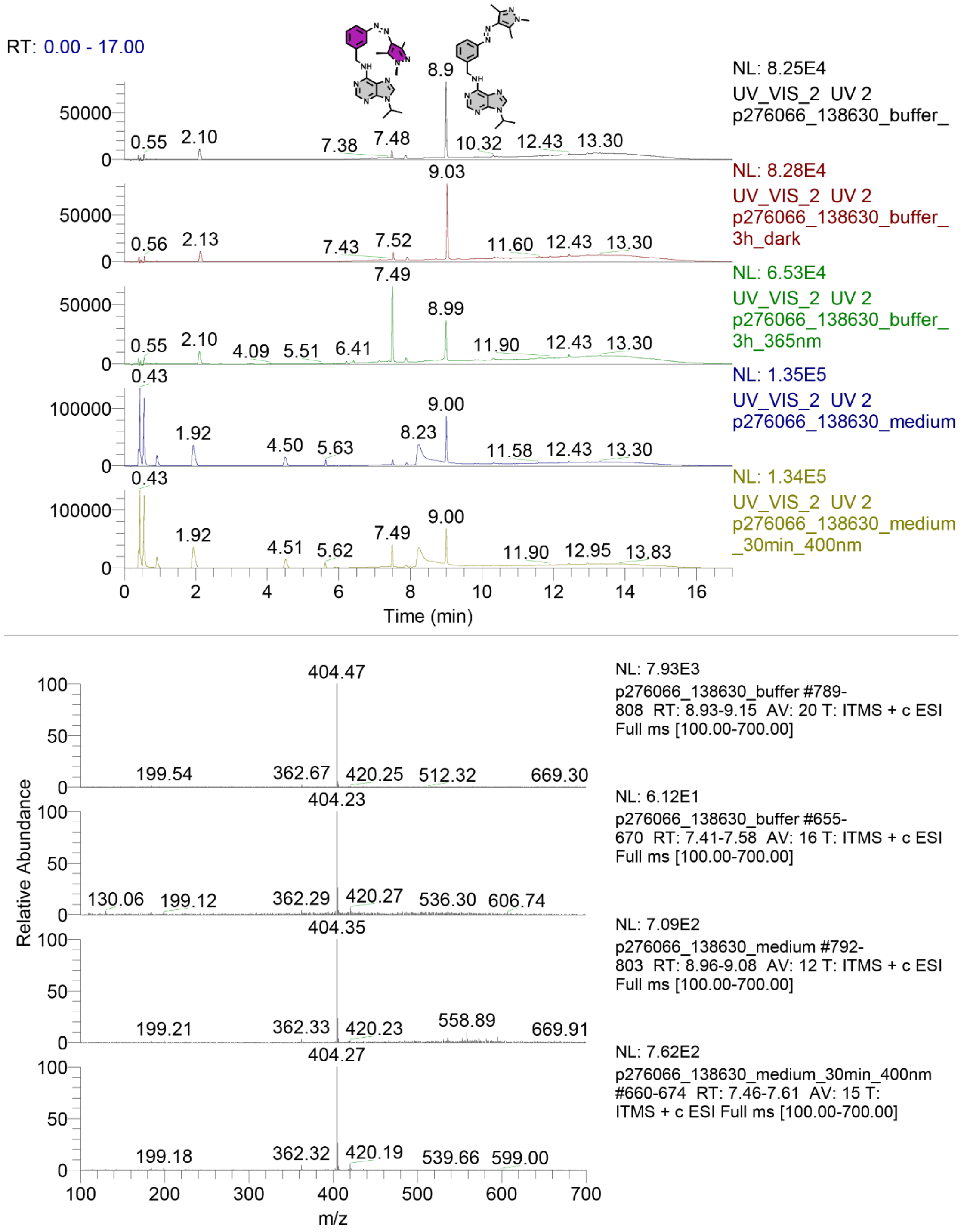

Supplementary Figure 13. Chemical stability of compound **8** under dark and irradiation conditions followed by LCMS. All the samples were prepared as 40 µM solution in kinase assay buffer or cell culture medium. 'Buffer' and 'medium' samples were injected immediately upon dissolving compound **8**, 'buffer_3h_dark' was injected after 3 h of keeping the solution in dark, 'buffer_3h_365nm' after irradiation of the sample for 3 h with UV light in kinase assay buffer, and 'medium_30min_400nm' after 30 min of irradiation with 400 nm in cell culture medium. The *trans*-isomer was followed at the retention time of 8.99-9.03 min and the *cis*-isomer at the retention time of 7.49-7.52 min. Mass spectra of both isomers in different media are also shown. Products of reduction or oxidation were not observed.

Compound **9**

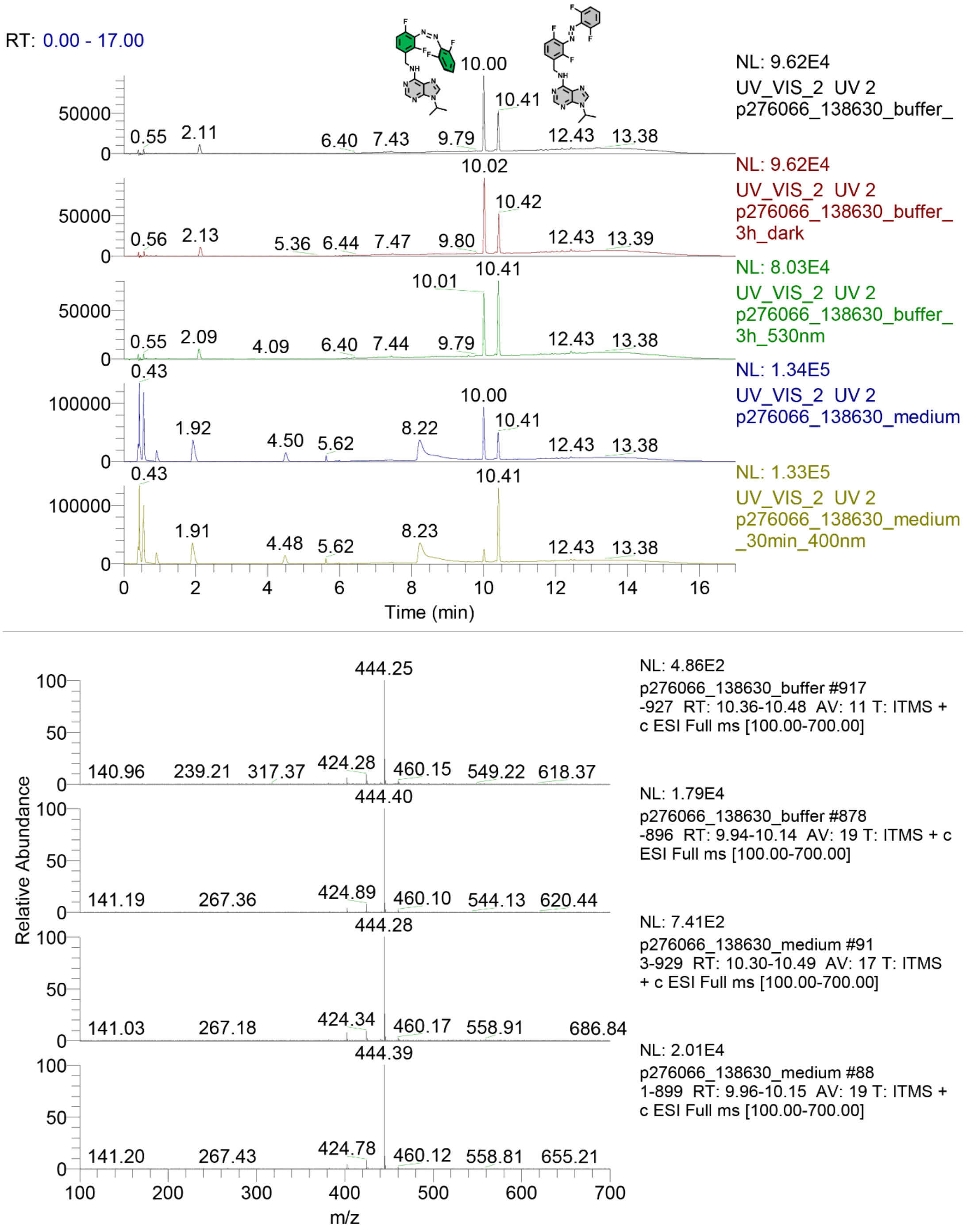

Supplementary Figure 14. Chemical stability of compound **9** under dark and irradiation conditions followed by LCMS. All the samples were prepared as 40 µM solution in kinase assay buffer or cell culture medium. 'Buffer' and 'medium' samples were injected immediately upon dissolving compound **9**, 'buffer_3h_dark' was injected after 3 h of keeping the solution in dark, 'buffer_3h_365nm' after irradiation of the sample for 3 h with UV light in kinase assay buffer, and 'medium_30min_400nm' after 30 min of irradiation with 400 nm in cell culture medium. The *trans*-isomer was followed at the retention time of 10.41-10.42 min and the *cis*-isomer at the retention time of 10.00-10.02 min. Mass spectra of both isomers in different media are also shown. Products of reduction or oxidation were not observed.

Half-life measurements

Compound **3**

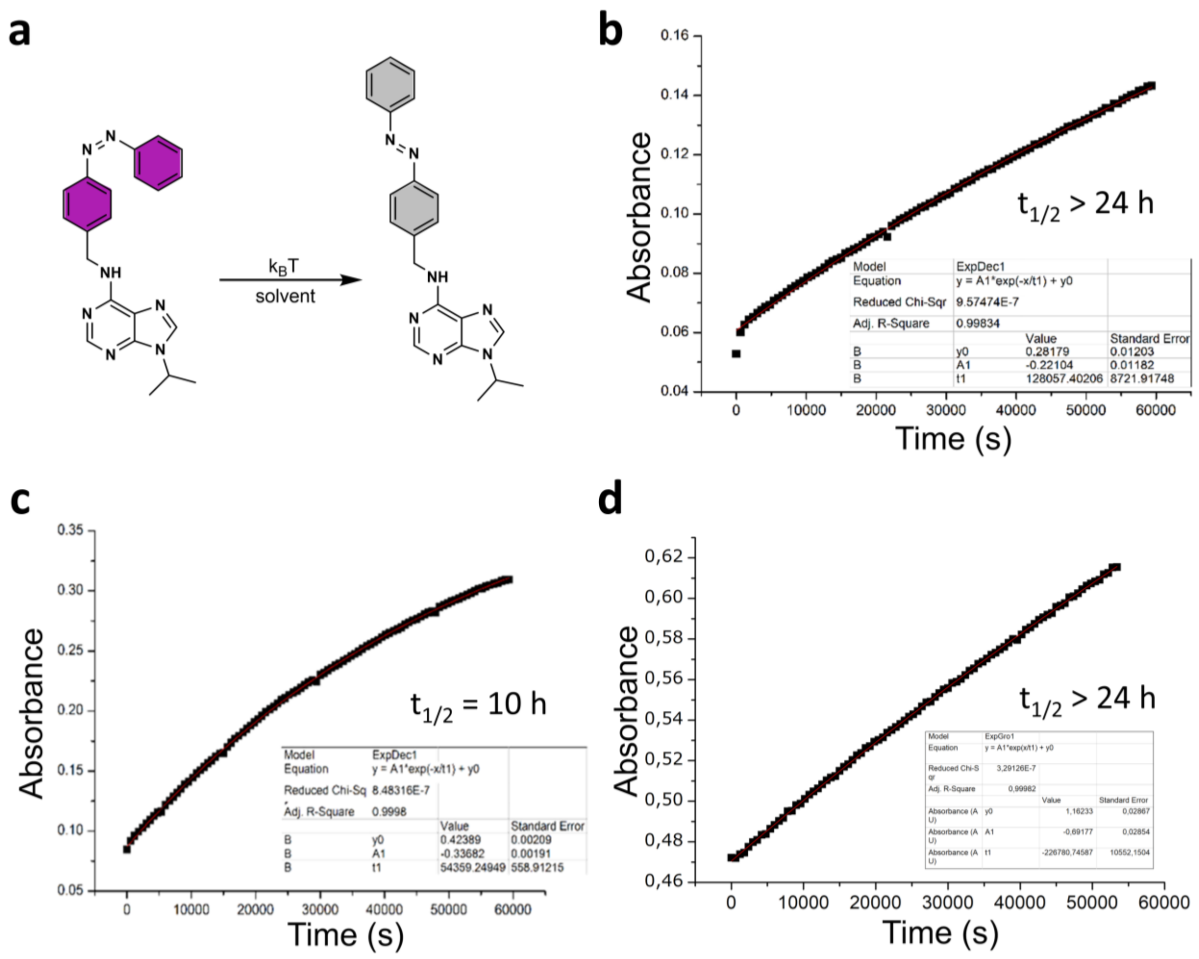

Supplementary Figure 15. Half-life determination for compound **3**. (**a**) Scheme of thermal back-isomerization of compound **3**. (**b**) Determination of half-life for **3** in DMSO (25 °C, ~20 µM), (**c**) kinase assay buffer (30 °C, ~20 µM) and (**d**) cell culture medium (35 °C, ~80 µM). Photostationary state was reached upon irradiation with λ = 365 nm. Red line presents the fitting with single exponential process.

Compound **4**

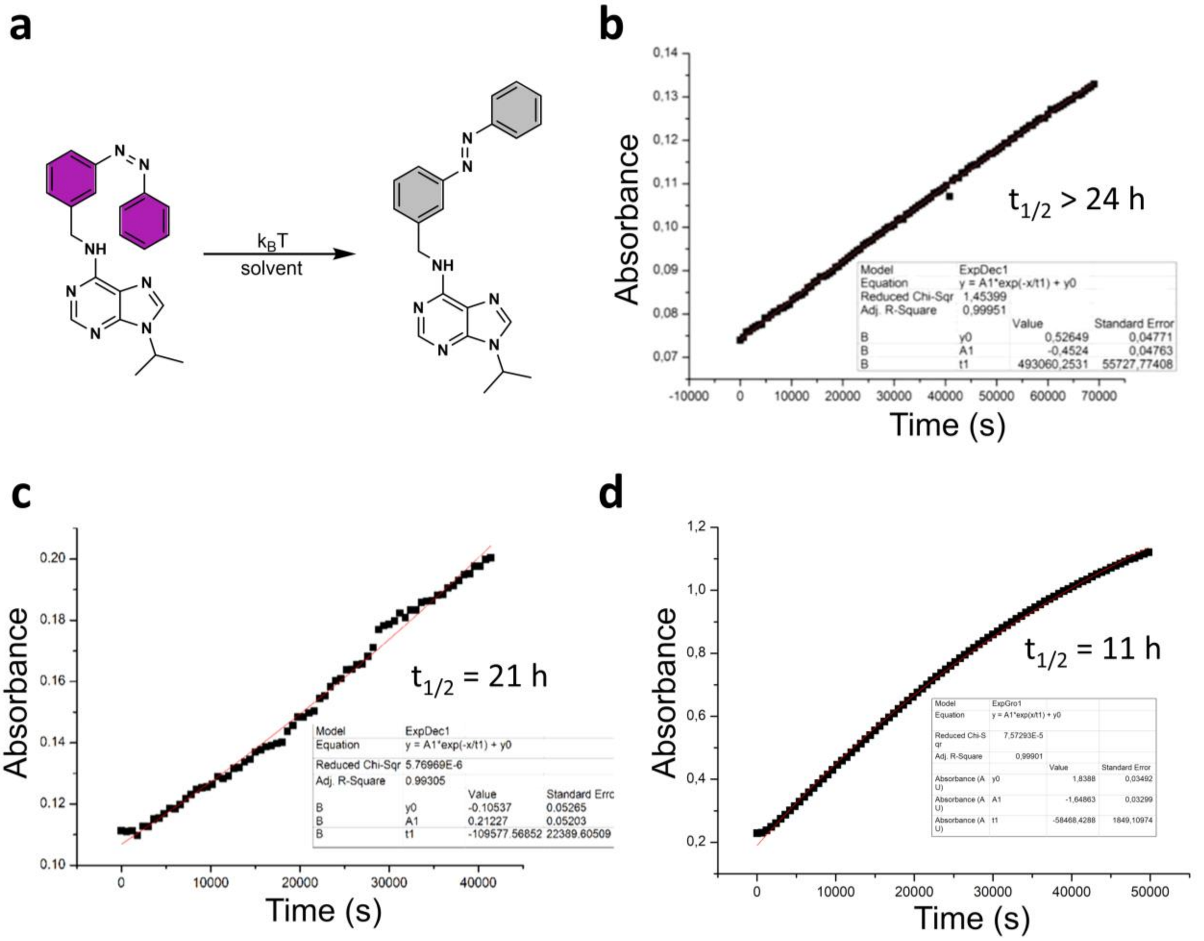

Supplementary Figure 16. Half-life determination for compound **4**. (**a**) Scheme of thermal back-isomerization of compound **4**. (**b**) Determination of half-life for **4** in DMSO (25 °C, ~20 µM), (**c**) kinase assay buffer (30 °C, ~20 µM) and (**d**) cell culture medium (35 °C, ~80 µM). Photostationary state was reached upon irradiation with λ = 365 nm. Red line presents the fitting with single exponential process.

Compound **5**

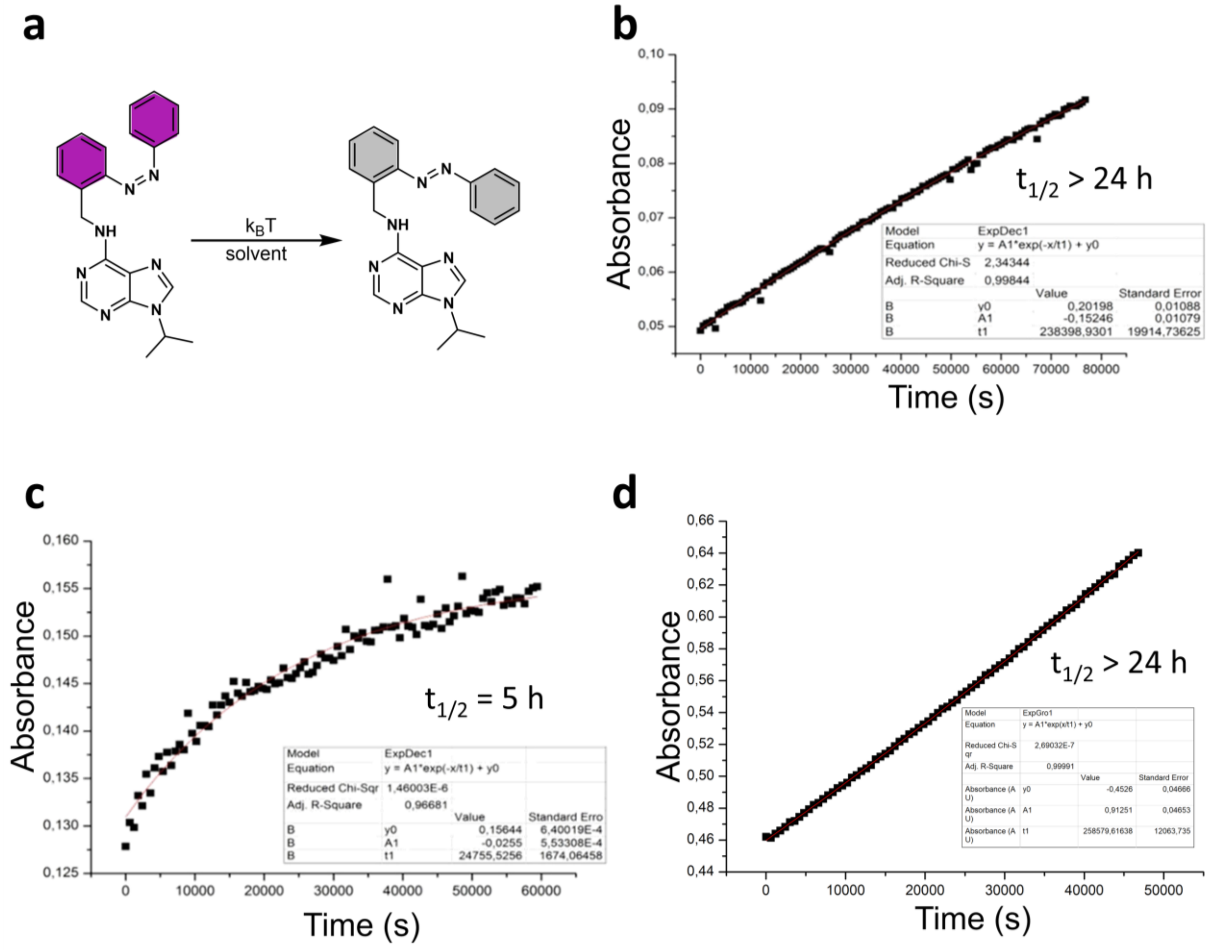

Supplementary Figure 17. Half-life determination for compound **5**. (**a**) Scheme of thermal back-isomerization of compound **5**. (**b**) Determination of half-life for **5** in DMSO (25 °C, ~20 µM), (**c**) kinase assay buffer (30 °C, ~20 µM) and (**d**) cell culture medium (35 °C, ~80 µM). Photostationary state was reached upon irradiation with λ = 365 nm. Red line presents the fitting with single exponential process.

Compound **6**

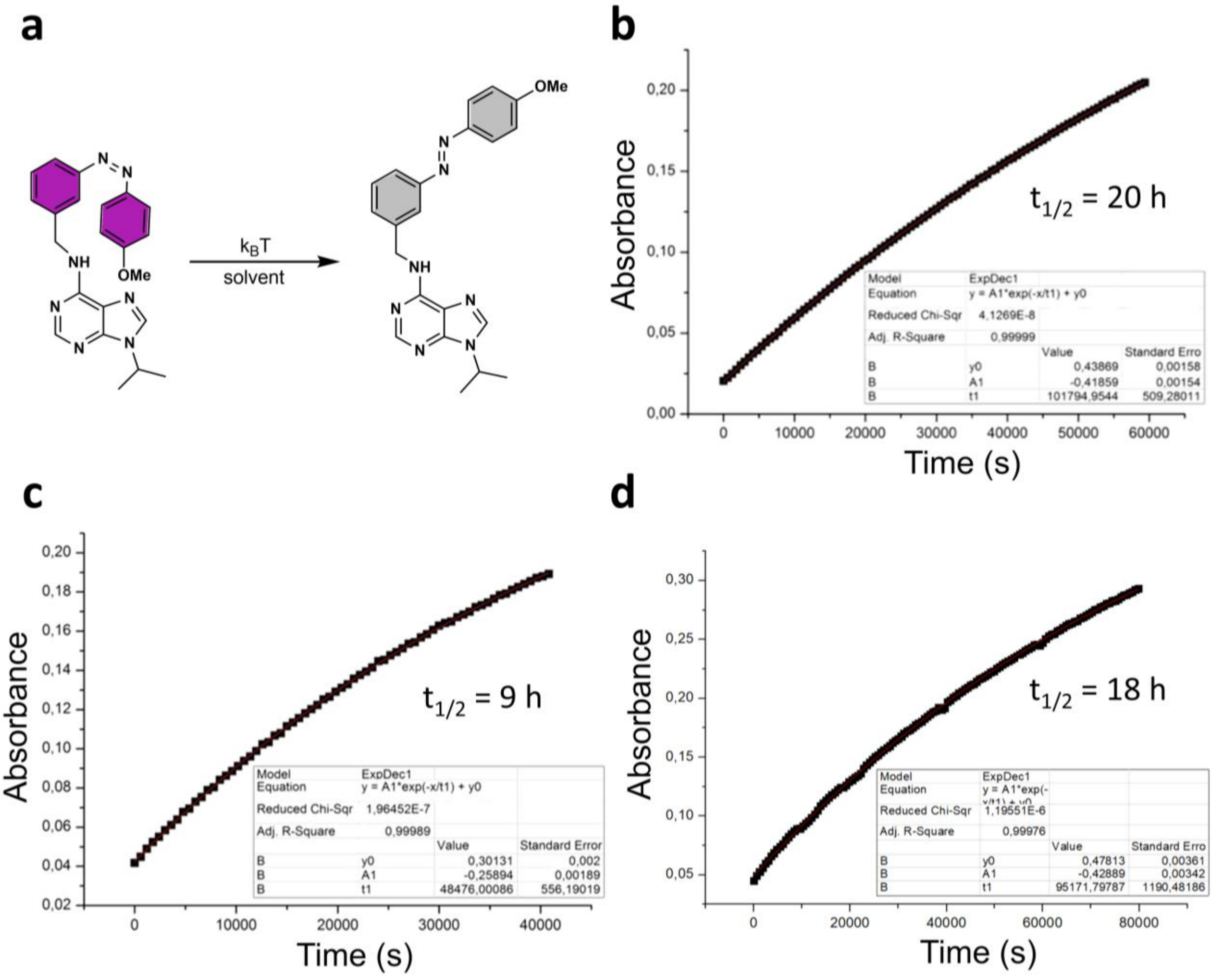

Supplementary Figure 18. Half-life determination for compound **6**. (**a**) Scheme of thermal back-isomerization of compound **6**. (**b**) Determination of half-life for **6** in DMSO (25 °C, ~20 µM), (**c**) kinase assay buffer (30 °C, ~20 µM) and (**d**) cell culture medium (35 °C, ~40 µM). Photostationary state was reached upon irradiation with λ = 365 nm. Red line presents the fitting with single exponential process.

Compound **7**

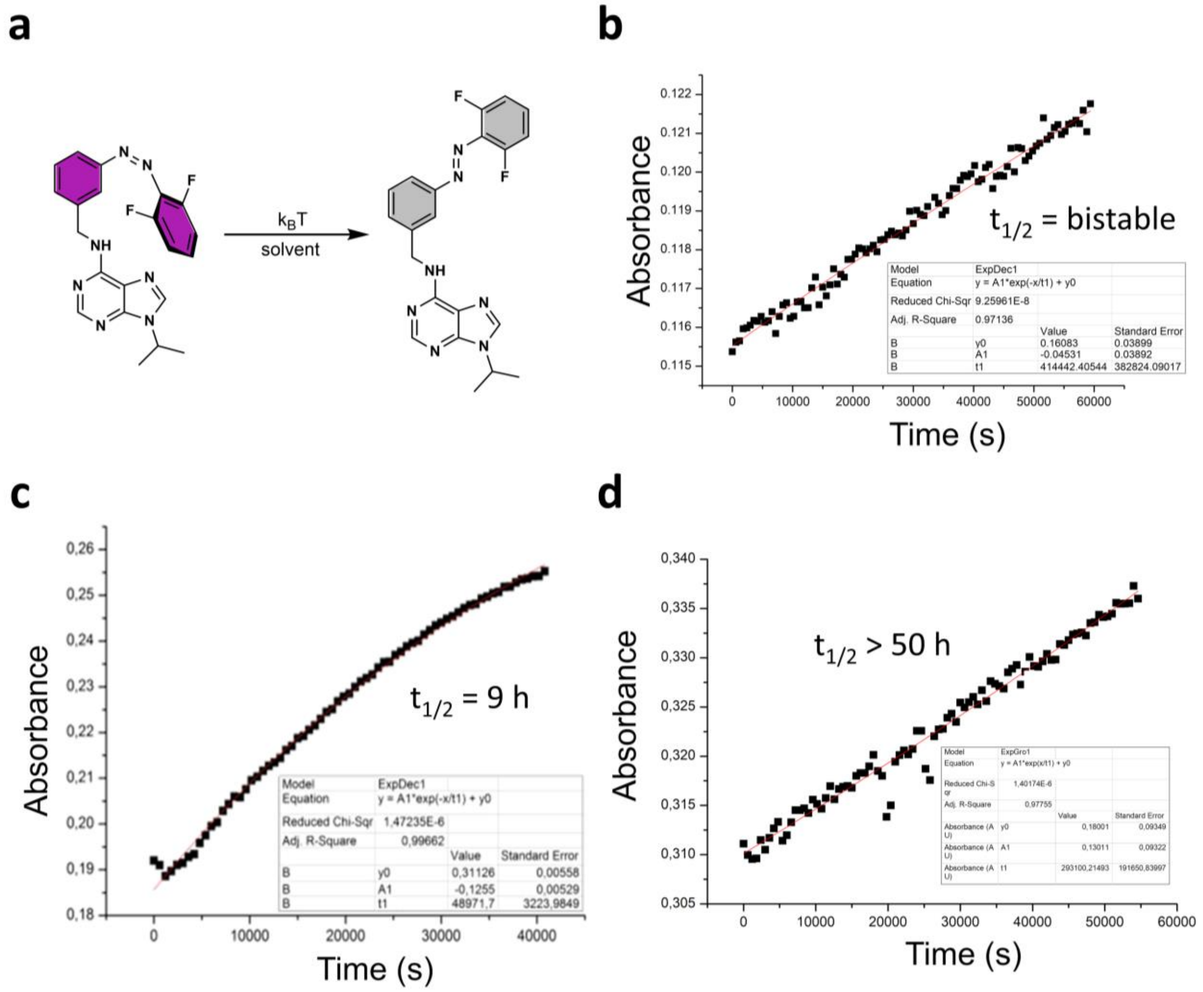

Supplementary Figure 19. Half-life determination for compound **7**. (**a**) Scheme of thermal back-isomerization of compound **7**. (**b**) Determination of half-life for **7** in DMSO (25 °C, ~20 µM), (**c**) kinase assay buffer (30 °C, ~20 µM) and (**d**) cell culture medium (35 °C, ~40 µM). Photostationary state was reached upon irradiation with λ = 365 nm. Red line presents the fitting with single exponential process.

Compound **8**

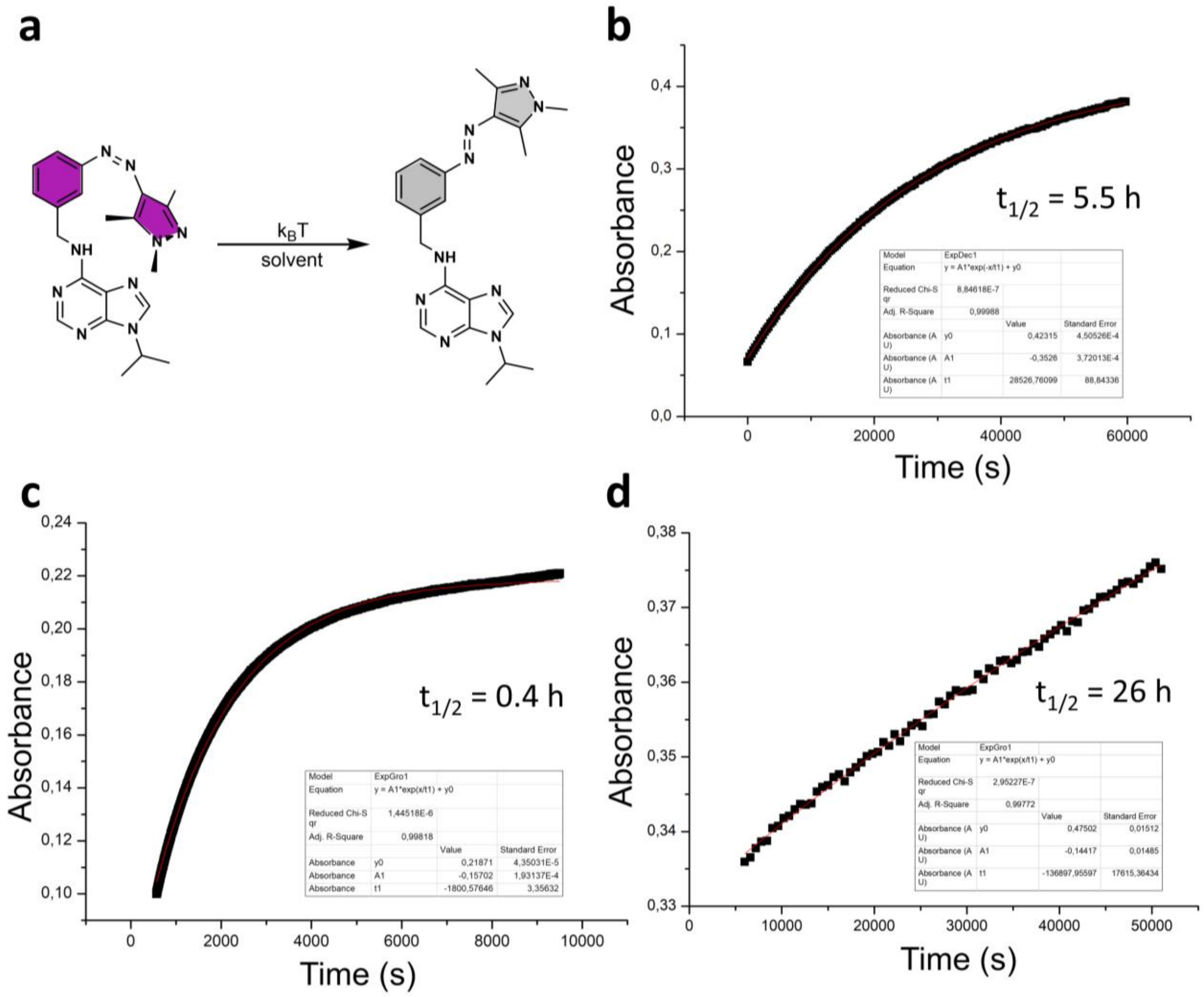

Supplementary Figure 20. Half-life determination for compound **8**. (**a**) Scheme of thermal back-isomerization of compound **8**. (**b**) Determination of half-life for **8** in DMSO (25 °C, ~20 µM), (**c**) kinase assay buffer (30 °C, ~20 µM) and (**d**) cell culture medium (35 °C, ~40 µM). Photostationary state was reached upon irradiation with λ = 365 nm. Red line presents the fitting with single exponential process.

Half-life comparison of **6**, **7** and **8**

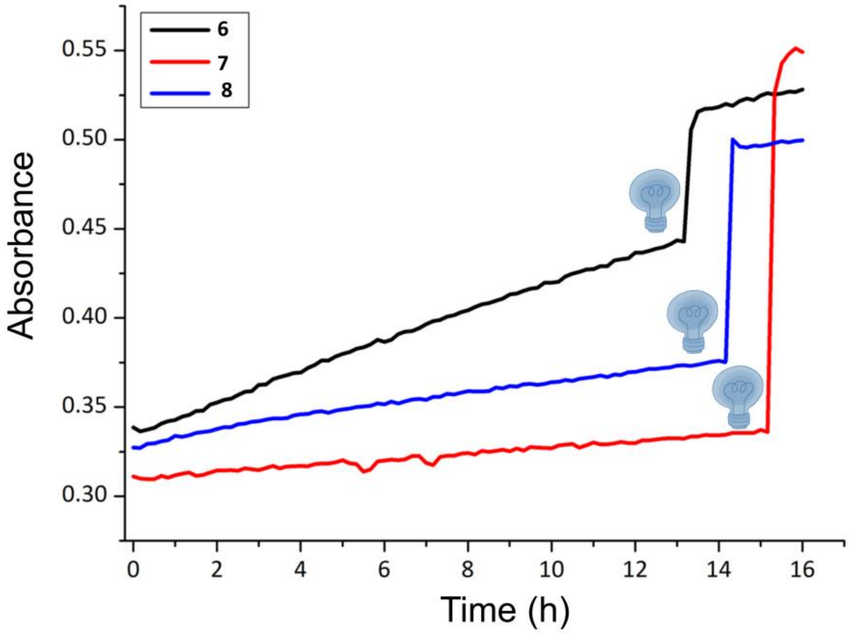

Supplementary Figure 21. Half-life comparison for compounds **6**, **7** and **8**. Thermal half-lives of modifiers **6** (black), **7** (red) and **8** (blue) in cell culture medium (35 °C, 40 µM). Upon reaching PSS distribution with UV light, thermal back-isomerization was followed in dark for 12-14 h. After this period blue light (blue bulb) was applied for 8 min to confirm presence of the remaining *cis*-isomer and estimate *cis*-to-*trans* ratio.

Compound **9**

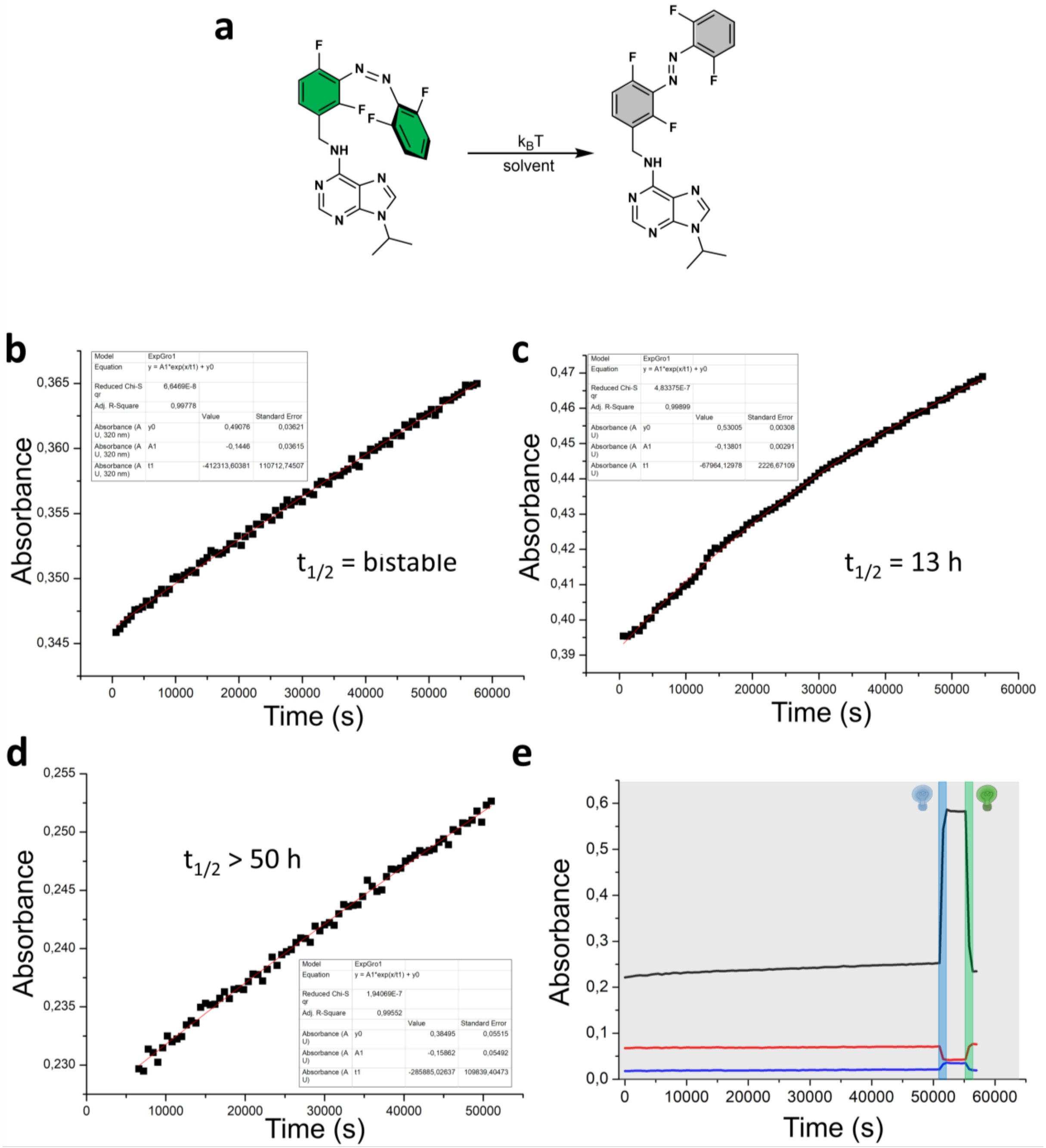

Supplementary Figure 22. Half-life determination for compound **9**. (**a**) Scheme of thermal back-isomerization of compound **9**. Determination of half-life for compound **9** in (**b**) DMSO (25 °C, ~20 µM), (**c**) kinase assay buffer (30 °C, ~20 µM) and (**d**) cell culture medium (35 °C, ~60 µM). (**e**) Upon reaching PSS distribution with green light (λ_{max} = 530 nm), thermal *cis*-to-*trans* isomerization was followed in cell culture medium (35 °C, 60 µM) in the dark for 14 h. After this period violet light (λ_{max} = 400 nm, blue rectangle) was applied for 8 min in order to confirm presence of the *cis*-isomer and estimate *cis*-to-*trans* ratio. After additional 1 h in the dark, *trans*-to-*cis* isomerization was again performed by irradiation with green light (λ_{max} = 530 nm, green rectangle).

Solubility test

Solubility studies were performed in kinase assay buffer and cell culture medium. Half of the solutions of the compounds in DMSO was thermally adapted (‘Dark’) and the other half was irradiated for 1 h with UV light (λ_{max} = 365 nm, ‘Light’) green light in case of **9** (λ_{max} = 530 nm, ‘Light’). Solubility is indicated in the **Supplementary** Table 1. Concentrations that showed precipitation were excluded from the dose-response curves in the enzymatic assay.

Supplementary Table 1. Solubility of the photoswitchable compounds in the highest concentrations that were used in the enzymatic assay.

		Kinase assay buffer						Cell culture medium					
		‘Dark’			‘Light’			‘Dark’			‘Light’		
Conc. (µM)		41	123	370	41	123	370	41	123	370	41	123	370
Compound	3	✔	✕	✕	✔	✔	✕	✔	✔	✕	✔	✔	✕
	4	✔	✕	✕	✔	✔	✕	✔	✔	✕	✔	✔	✕
	5	✔	✔	✔	✔	✔	✔	✔	✔	✔	✔	✔	✔
	6	✔	✕	✕	✔	✔	✕	✔	✔	✕	✔	✔	✔
	7	✔	✕	✕	✔	✕	✕	✔	✕	✕	✔	✔	✕
	8	✔	✔	✕	✔	✔	✕	✔	✔	✕	✔	✔	✔
	9	✔	✕	✕	✔	✕	✕	✔	✕	✕	✔	✔	✕

In vitro kinase assay

Reagents

The total volume for the reaction was 10.5 µl. The reaction mixture contained the following: for CKIδ, 4 ng/µl CKIδ (14-520, Eurofins), 50 µM peptide substrate RKKKAEpSVASLTSQCSYSS corresponding to human PER2 Lys659-Ser674, and CKI buffer (40 mM Tris, 10 mM $MgCl_2$, 0.5 mM DTT, 0.1 mg/ml BSA, pH7.4); for CKIα, 1 ng/µl CKIα (PV3850, Invitrogen), 50 µM CKI peptide substrate (60547-1, Anaspec), and CKI buffer.

Data analysis

The data obtained from the *in vitro* kinase assay was analyzed using GraphPad software, in which the luminescence outcome is normalized. Normalization is done using a luminescent output of the control that contained high concentration of longdaysin (370 µM) as 100% inhibition and DMSO for 0% inhibition. In all graphs, a dose-response fit was used to obtain the IC_{50} value.

Effect of longdaysin (**1**) on CKIα

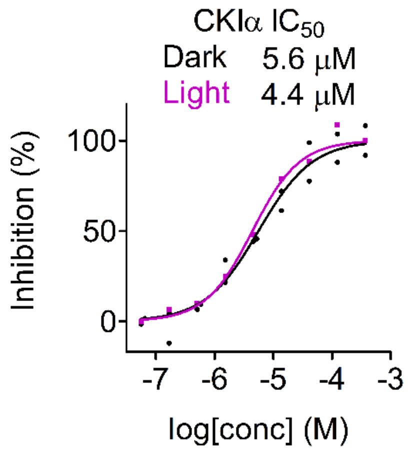

Supplementary Figure 23. Effect of longdaysin (**1**) on CKIα. Black and purple show dark and UV-irradiated conditions, respectively.

Evaluation of the circadian period photo-modulation

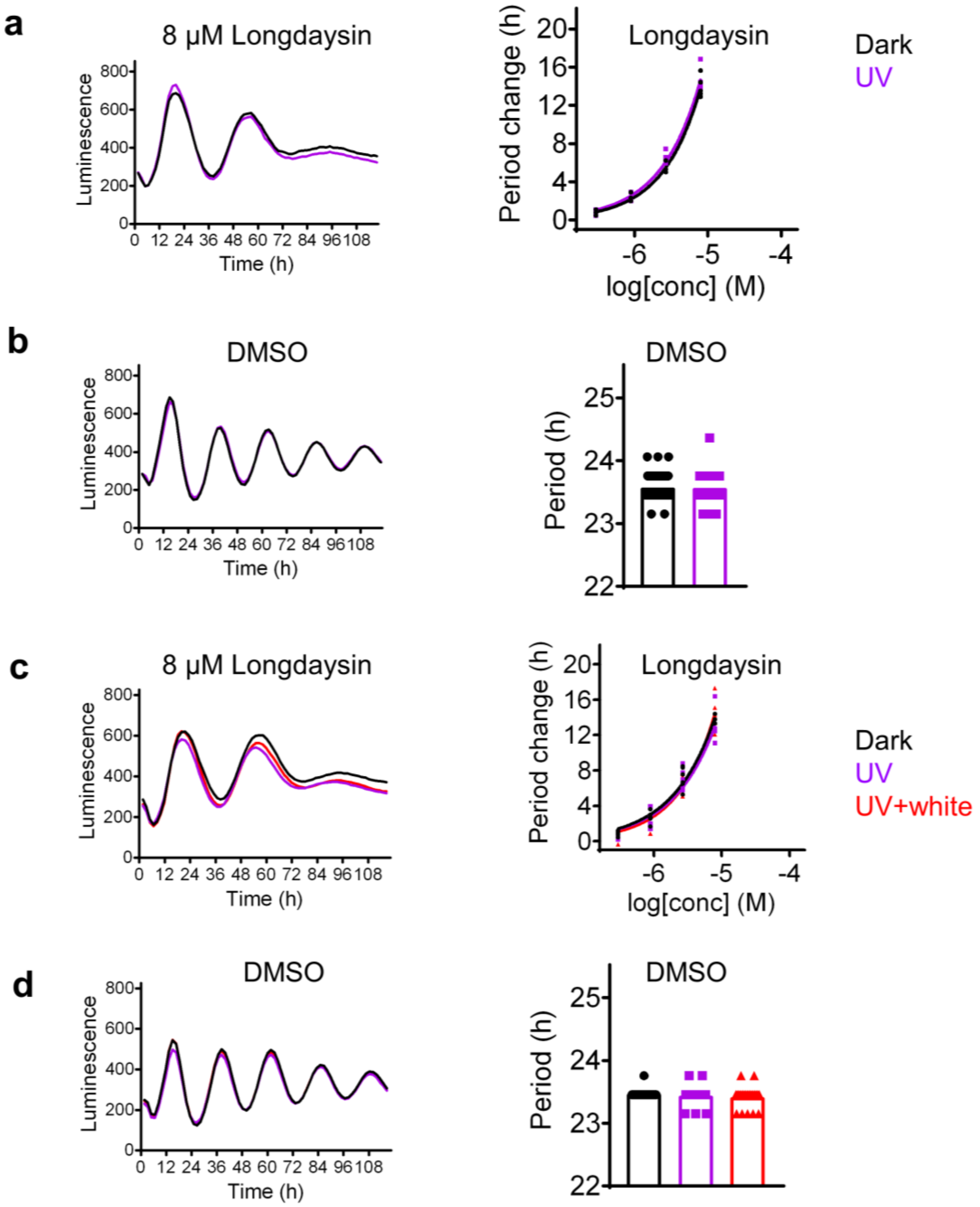

Supplementary Figure 24. Longdaysin and DMSO control experiments for compounds **3**-**8** of Figure 2d and 3c. n = 6 (**a**), 48 (**b**), 4-6 (**c**), and 24 (**d**) biologically independent samples.

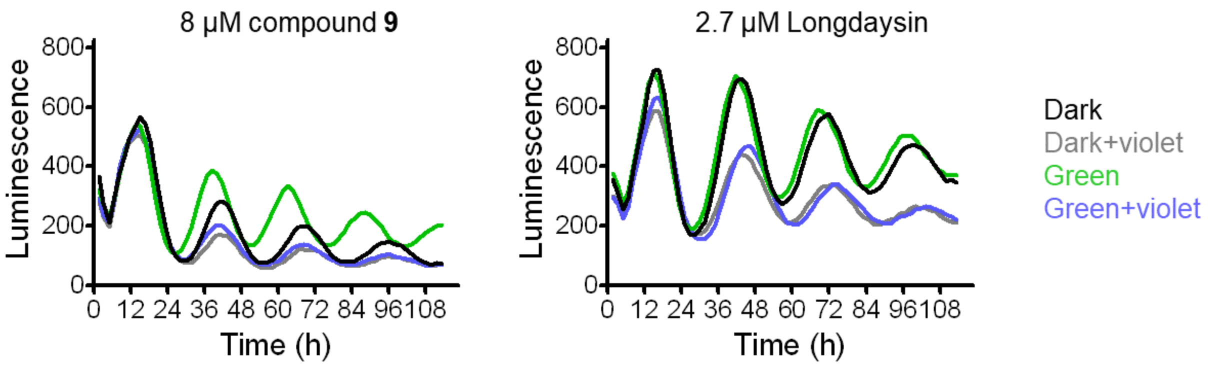

Supplementary Figure 25. Luminescence profiles for 8 μM compound **9** and 2.7 μM longdaysin of Figure 4e. Mean of n = 3 biologically independent samples.

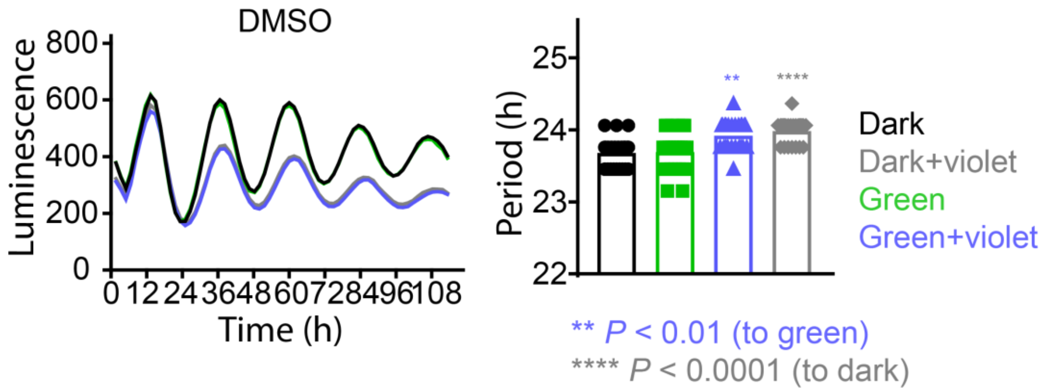

Supplementary Figure 26. DMSO control experiment for compound **9** of Figure 4e. n = 24 biologically independent samples. One-way ANOVA followed by a Tukey's multiple comparisons test was used for statistical analysis. *P* value was shown in the figure.

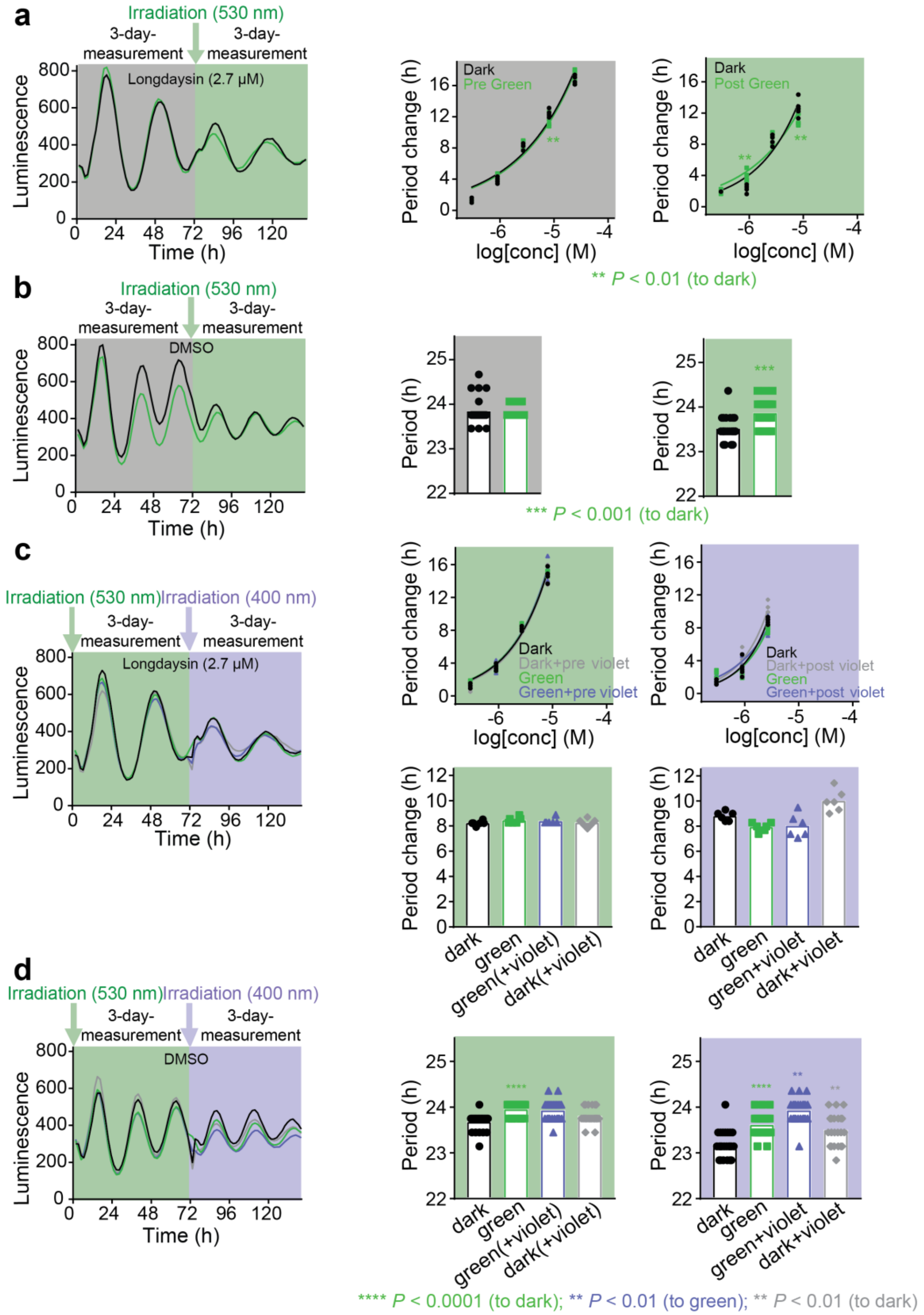

Supplementary Figure 27. Longdaysin and DMSO control experiments for long-term photomodulation of Figure 5. n = 4-6 (**a**), 23-24 (**b**), 6 (**c**), and 24 (**d**) biologically independent samples. Two-way ANOVA followed by a Sidak's multiple comparisons test (**a** and **c**, top panels), two-sided Student's t-test (**b**), or one-way ANOVA followed by a Tukey's multiple comparisons test (**c**, bottom panels and **d**) was used for statistical analysis. *P* value was shown in the figure.

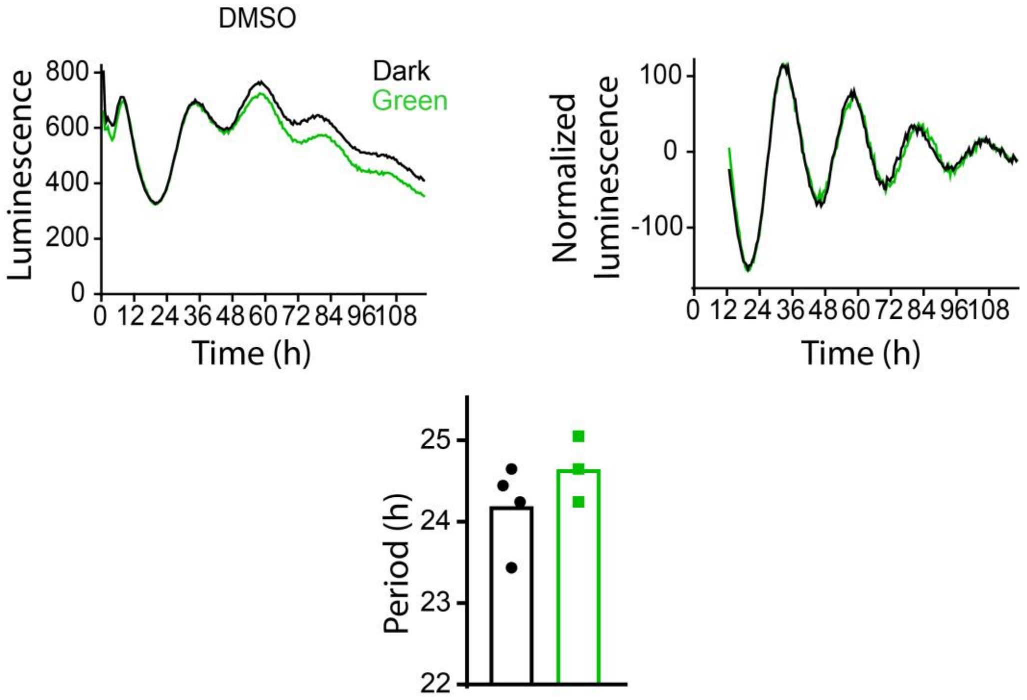

Supplementary Figure 28. The DMSO control experiment for mouse spleen explants of Figure 6a-c. n = 4 biologically independent samples for dark and n = 3 for 530 nm light.

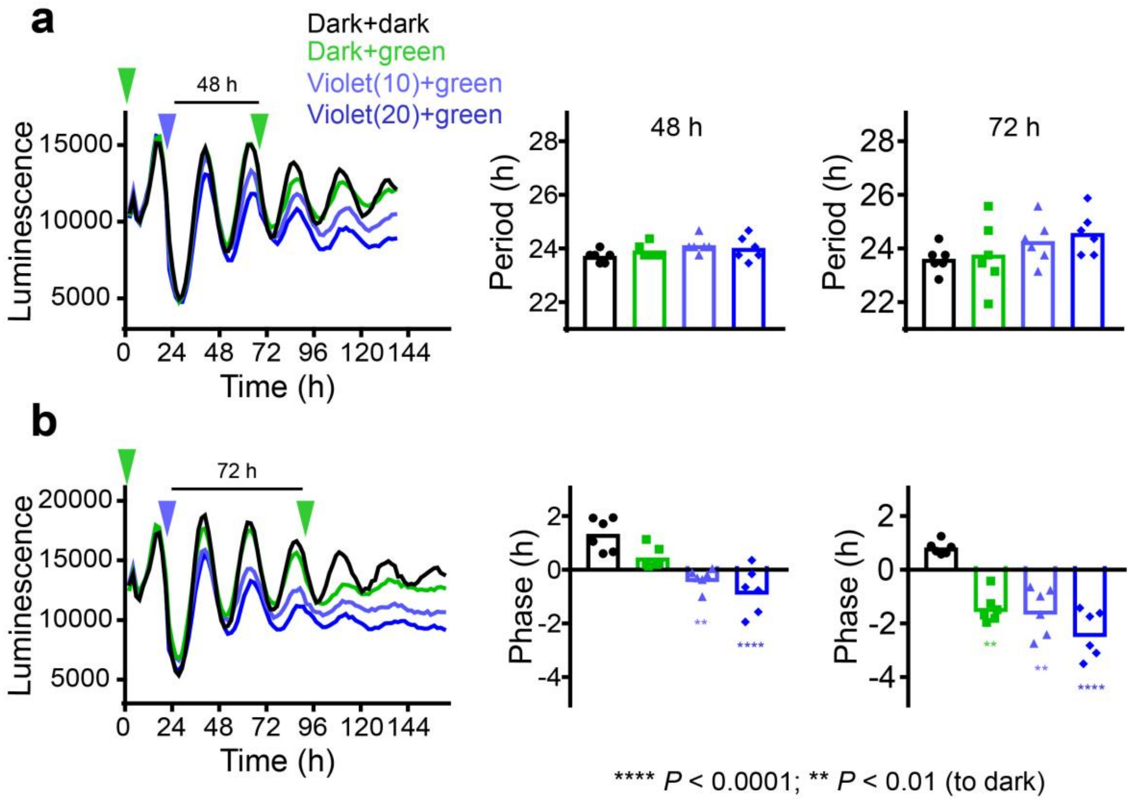

Supplementary Figure 29. DMSO control experiments for phase regulation of Figure 7. n = 6 biologically independent samples. One-way ANOVA followed by a Tukey's multiple comparisons test was used for statistical analysis. *P* value was shown in the figure.

UV-vis absorption spectra and fatigue studies

Luciferin

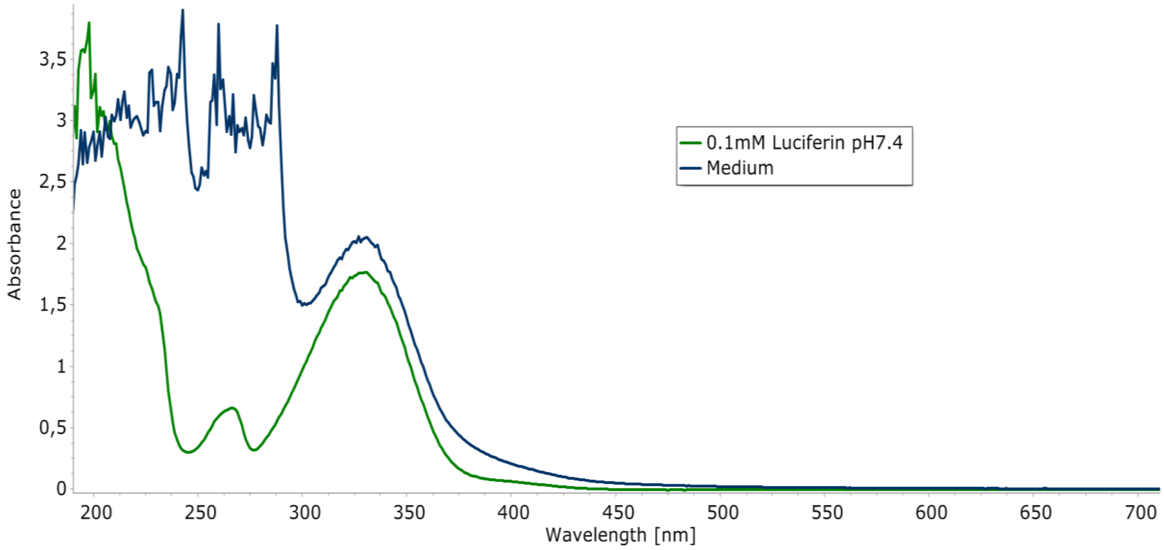

Supplementary Figure 30. Absorption spectrum of luciferin and of cell culture medium containing luciferin. The spectrum is measured at pH 7.4 (0.1 mM in water) and in presence of 0.1 mM concertation of luciferin.

Compound **3**

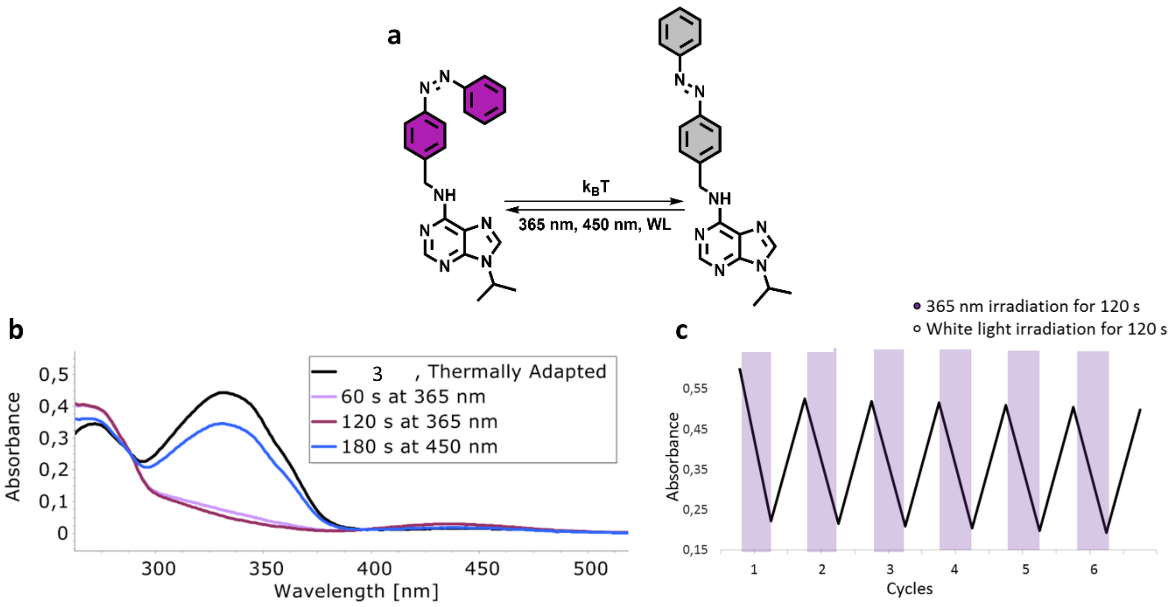

Supplementary Figure 31. Photochemical properties of compound **3**. (**a**) Isomerization process scheme. (**b**) Switching studies in DMSO (20 µM, 30 °C) with 365 nm and 450 nm light. (**c**) Fatigue determination in a DMSO solution (20 µM). Cycles performed by irradiating with 365 nm for 120 s, followed by irradiation with white light for 120 sec.

Compound **4**

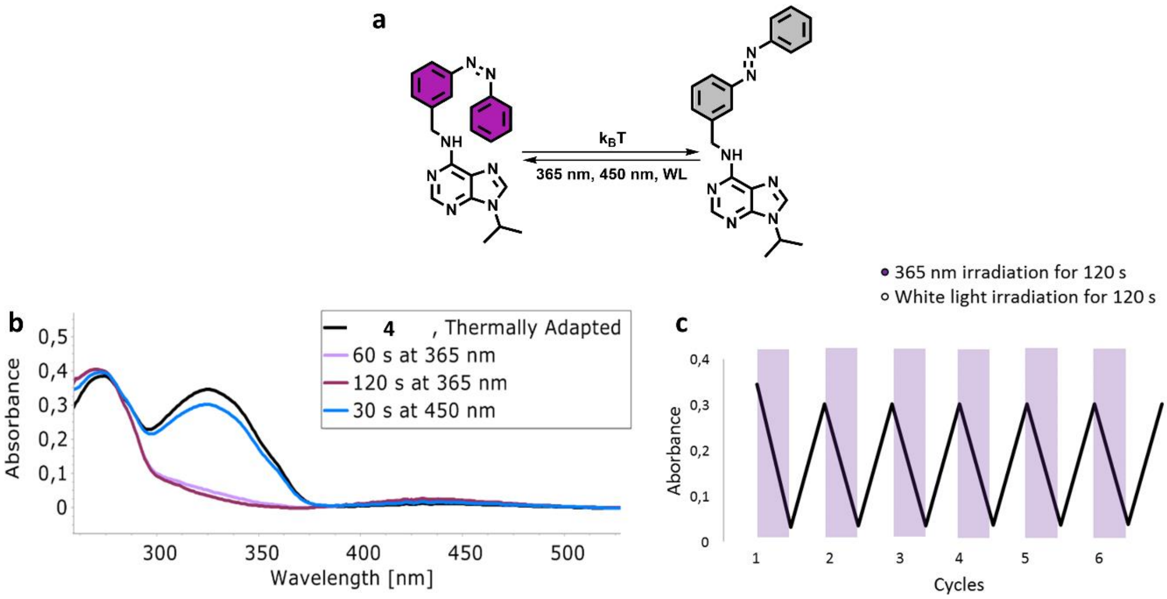

Supplementary Figure 32. Photochemical properties of compound **4**. (**a**) Isomerization process scheme. (**b**) Switching studies in DMSO (20 µM, 30 °C) with 365 nm and 450 nm light. (**c**) Fatigue determination in a DMSO solution (20 µM). Cycles performed by irradiating with 365 nm for 120 s, followed by irradiation with white light for 120 sec.

Compound **5**

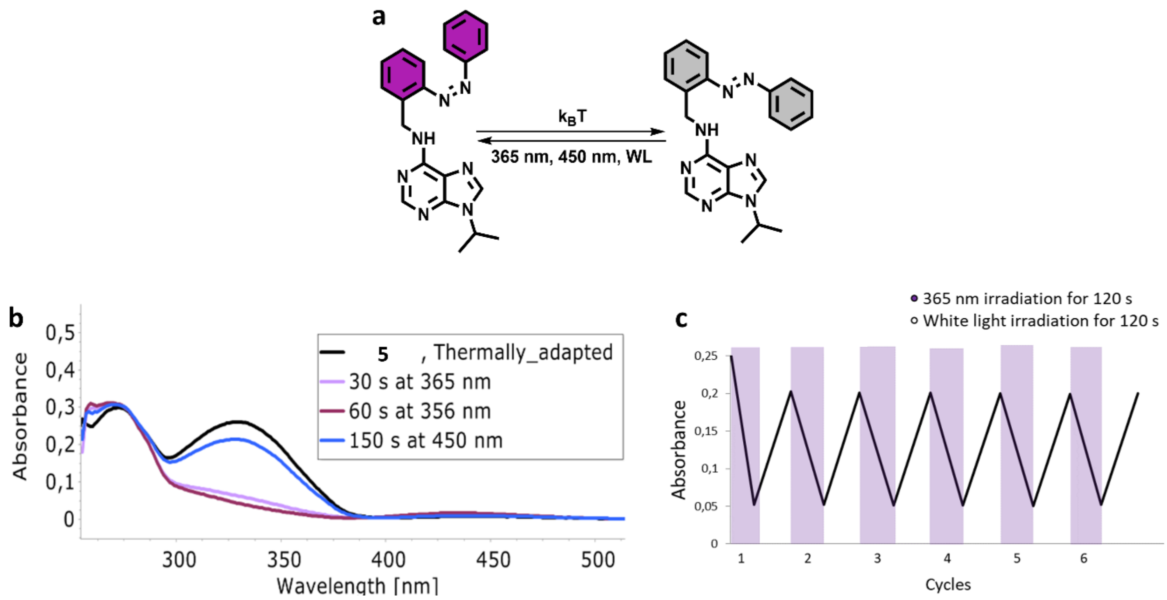

Supplementary Figure 33. Photochemical properties of compound **5**. (**a**) Isomerization process scheme. (**b**) Switching studies in DMSO (20 µM, 30 °C) with 365 nm and 450 nm light. (**c**) Fatigue determination in a DMSO solution (20 µM). Cycles performed by irradiating with 365 nm for 120 s, followed by irradiation with white light for 120 sec.

Compound **6**

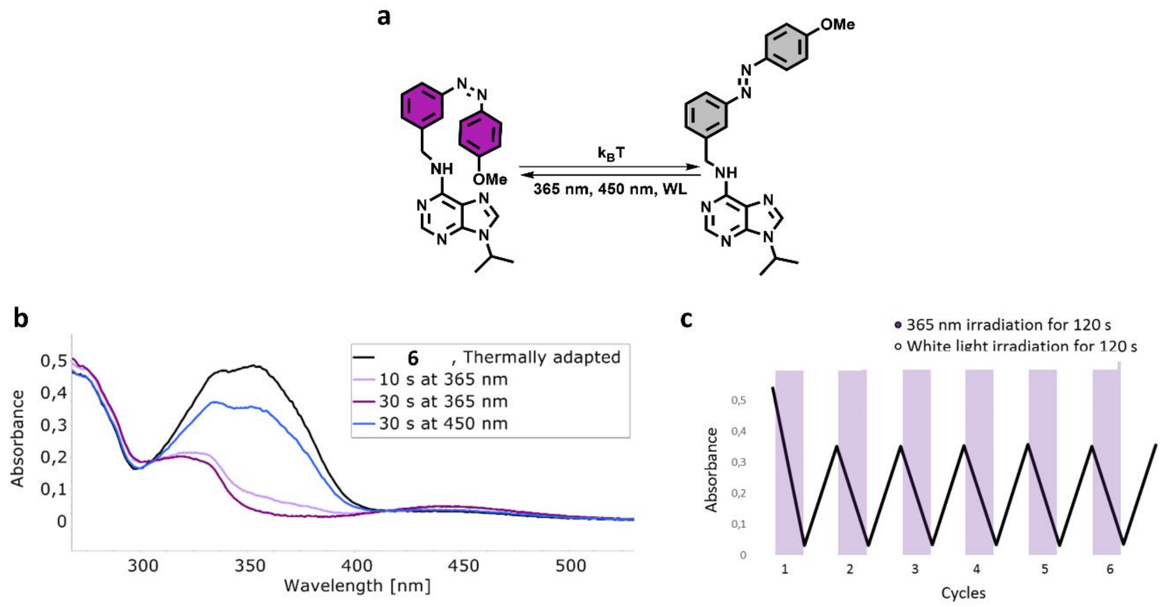

Supplementary Figure 34. Photochemical properties of compound **6**. (**a**) Isomerization process scheme. (**b**) Switching studies in DMSO (20 µM, 30 °C) with 365 nm and 450 nm light. (**c**) Fatigue determination in a DMSO solution (20 µM). Cycles performed by irradiating with 365 nm for 120 s, followed by irradiation with white light for 120 sec.

Compound **7**

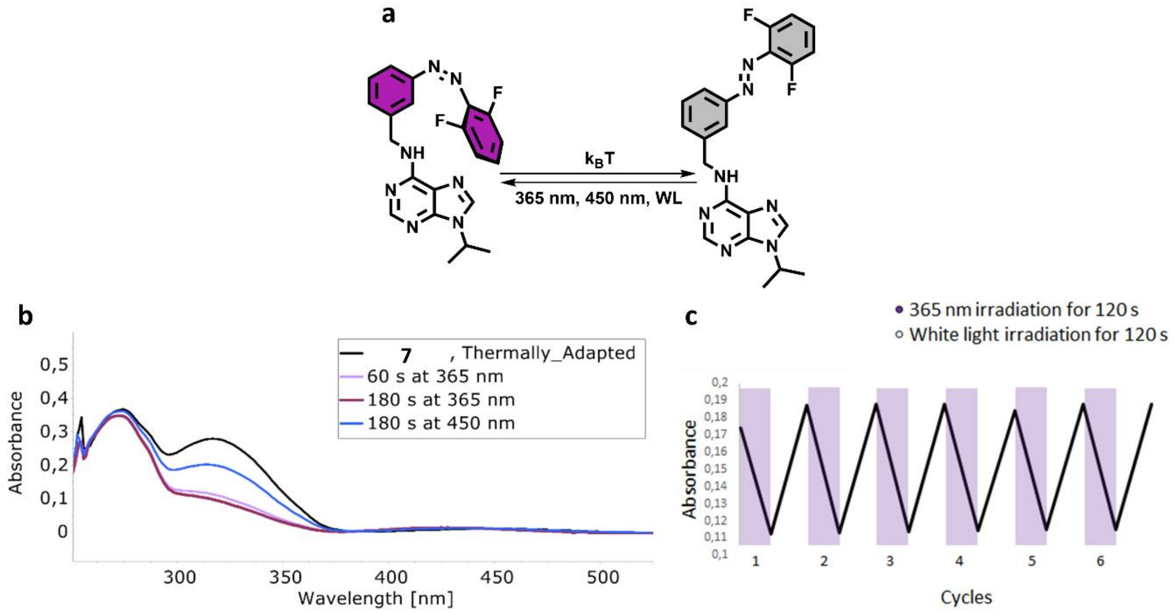

Supplementary Figure 35. Photochemical properties of compound **7**. (**a**) Isomerization process scheme. (**b**) Switching studies in DMSO (20 µM, 30 °C) with 365 nm and 450 nm light. (**c**) Fatigue determination in a DMSO solution (20 µM). Cycles performed by irradiating with 365 nm for 120 s, followed by irradiation with white light for 120 sec.

Compound **8**

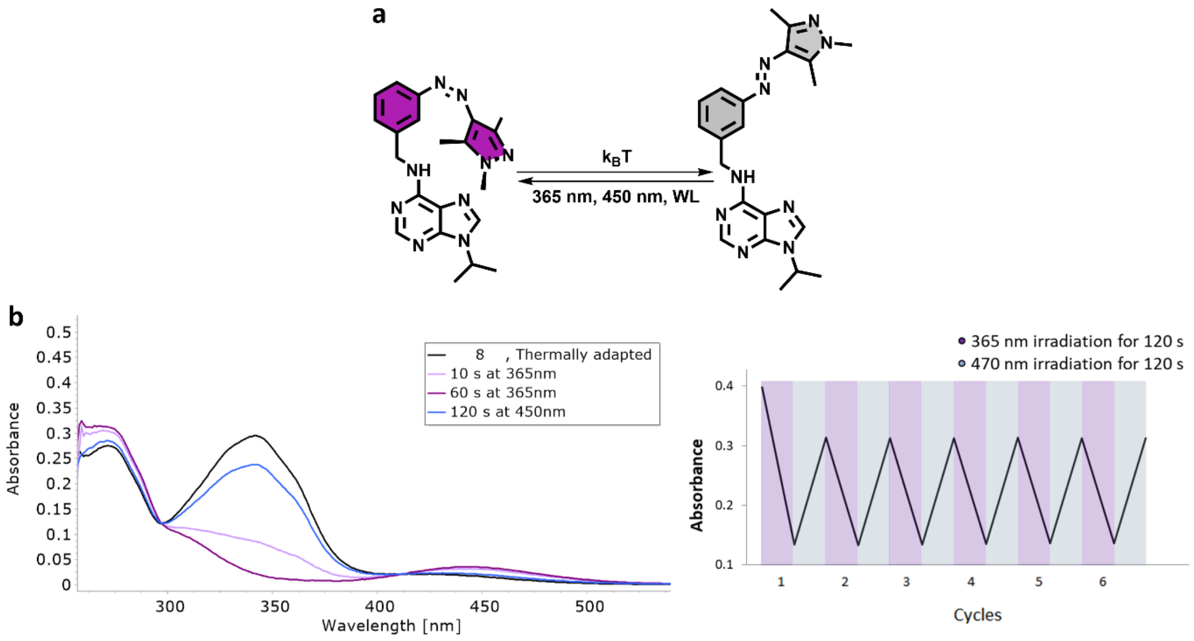

Supplementary Figure 36. Photochemical properties of compound **8**. (**a**) Isomerization process scheme. (**b**) Switching studies in DMSO (20 µM, 30 °C) with 365 nm and 450 nm light. (**c**) Fatigue determination in a DMSO solution (20 µM). Cycles performed by irradiating with 365 nm for 120 s, followed by irradiation with 470 nm for 120 sec.

Compound **9**

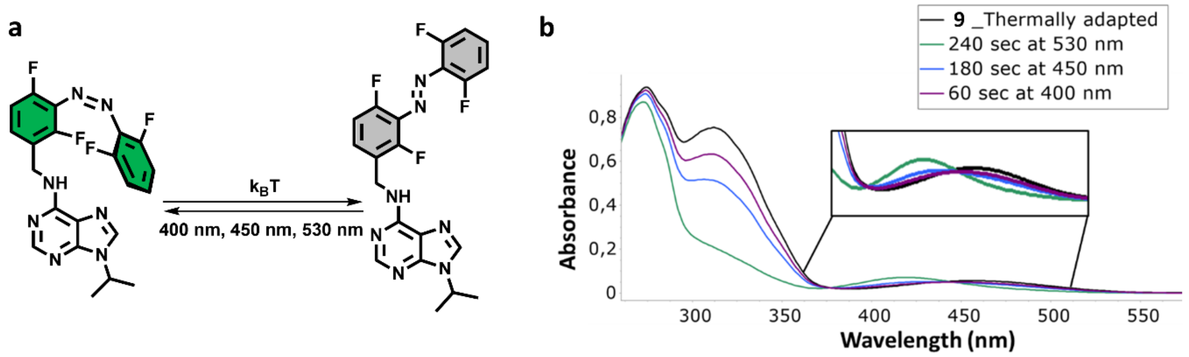

Supplementary Figure 37. Photochemical properties of compound **9**. (**a**) Isomerization process scheme. (**b**) Switching studies in DMSO (20 µM, 30 °C) with 530 nm, 450 nm and 400 nm light.

Molecular docking

Molecular docking analysis of the photoinduced differences in binding

To explain and rationalize the obtained experimental results and interactions of our photoswitchable kinase inhibitors, molecular docking of all photoswitchable modulators with CK1α was performed. Longdaysin and its derivatives are known to bind to the ATP-binding site of CK1α and CK1δ.[4] Most of these inhibitors utilize the canonical hydrogen bond between ligand and the backbone of the hinge region residue (L93 and L85 in CK1α and CK1δ respectively). All the modulators studied here (except *cis*-**4**) dock in the ATP binding site with at least one binding mode utilizing the hydrogen bond interaction with amide proton and/or amide oxygen of L93 (Supplementary Figure 39-45). Considering the large volume of the binding pocket, it is not surprising that all the modulators show binding both in *trans* and *cis* form. We further calculated binding energies of the docked complexes using the MM-GBSA method.[5,6]

Optimization of the docked complexes by short MD simulations has been known to provide better estimation of the binding energies.[5,7,8] Therefore, in order to understand the difference between the docked complexes of *trans* and *cis* form of different modulators we performed short MD simulations and calculated binding energies using frames of these MD simulation trajectories. The *trans* form of **3**, **4**, **6**, **7,** and **8** show lower average binding energies as compared to their corresponding *cis* forms (**Error! Reference source not found.**38a). On the other hand, the *cis* form of **5** shows lower binding energy as compared to *trans* form (**Error! Reference source not found.**38a). The observations are consistent with experimental results.

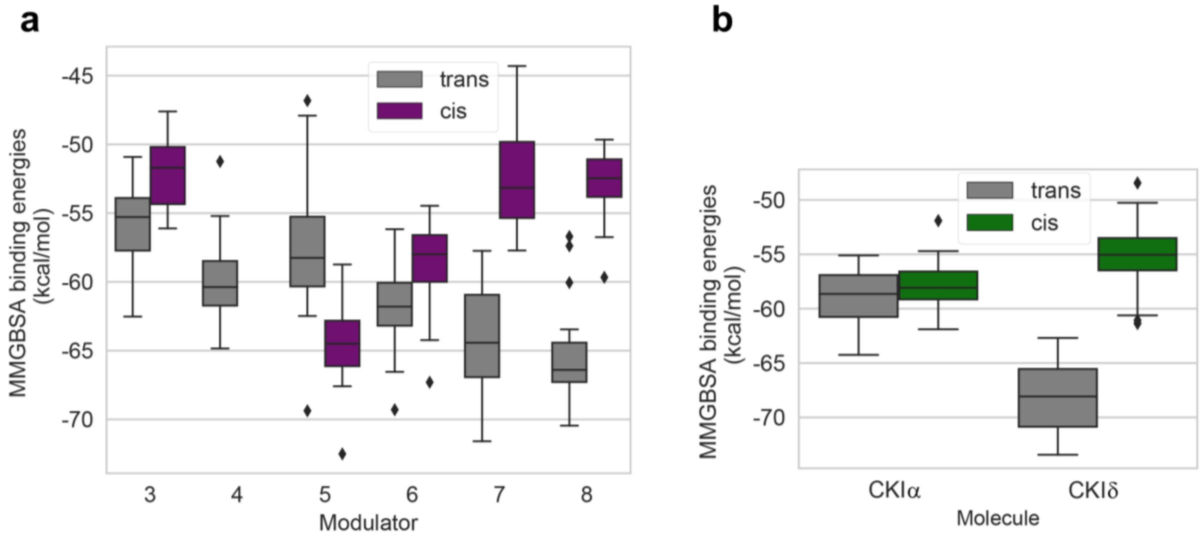

Supplementary Figure 38. MM-GBSA binding energies (in kcal/mol) for modulators (**a**) **3**-**8** and (**b**) **9** in *trans* and *cis* forms. For *cis*-**9**, MM-GBSA binding energies are given for α and δ isoforms of the CKI protein. *Cis*-**4** did not give a binding mode with hinge region interaction with any of the CK1α structures. The bounded boxes show the interquartile range, the whiskers show the whole range of the distribution with minima and maxima as horizontal

lines, the horizontal line within the box shows median. The outliers have been shown as separate black diamond markers.

In order to understand the isoform dependent differences of the compound effects, we performed the docking analysis of *trans*-**9** and *cis*-**9** to CK1δ crystal structures (Supplementary Figure 38b). High resolution CK1δ crystal structures with no missing residues near the active site were selected. This gave 23 individual chains of CK1δ in complex with different ligand molecules. These crystal structures were then clustered based on the binding site residues and 13 individual chains were selected representing different clusters (PDB 3UYT:C, 3UZP:A, 4HNF:B, 4KBK:B, 4TN6:A 4TN6:B, 4TW9:A, 4TWC:A, 5IH6:A, 5MQV:A, 5OKT:A, 5W4W:A, 6GZM:A). Same protocol as described for CK1α docking was followed for each system. The docked complexes with ligand binding mode similar to longdaysin and hydrogen bonds with the hinge residue L85 were selected.

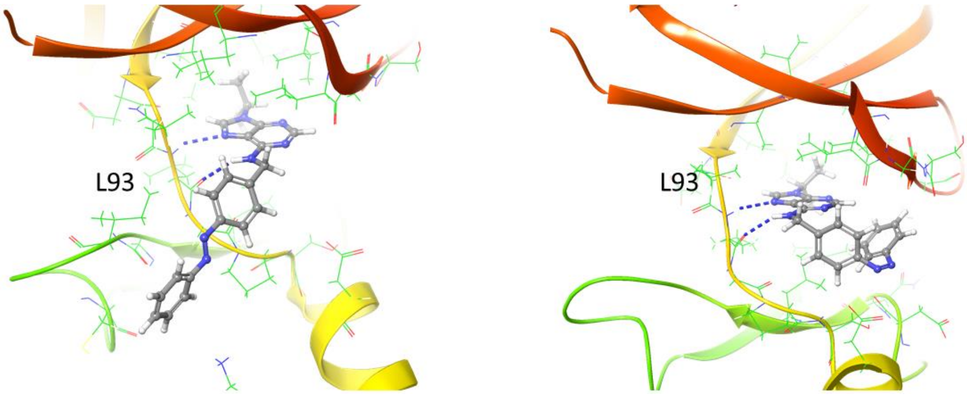

Supplementary Figure 39. Binding mode of *trans*-**3** (A) and *cis*-**3** (B). Modes are obtained after Glide XP docking and further used for MD simulations.

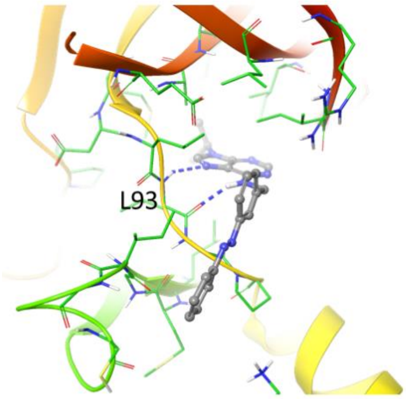

Supplementary Figure 40. Binding mode of *trans*-**4** (A). Modes are obtained after Glide XP docking and further used for MD simulations.

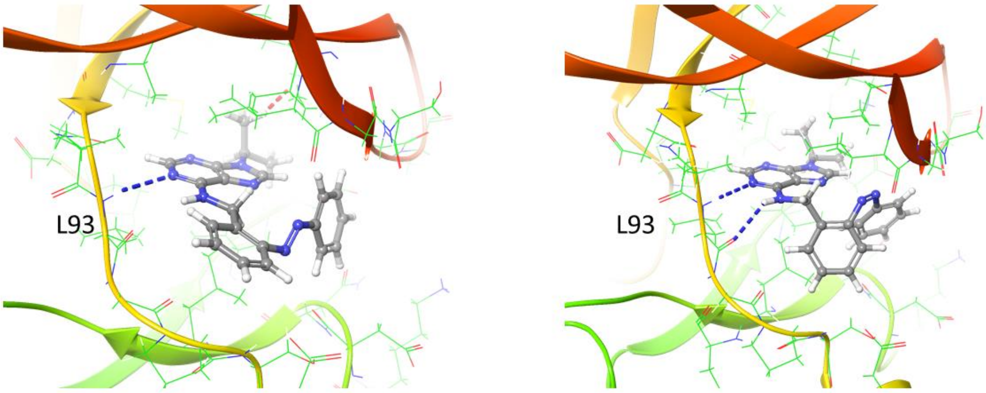

Supplementary Figure 41. Binding mode of *trans*-**5** and *cis*-**5**. Modes are obtained after Glide XP docking and further used for MD simulations.

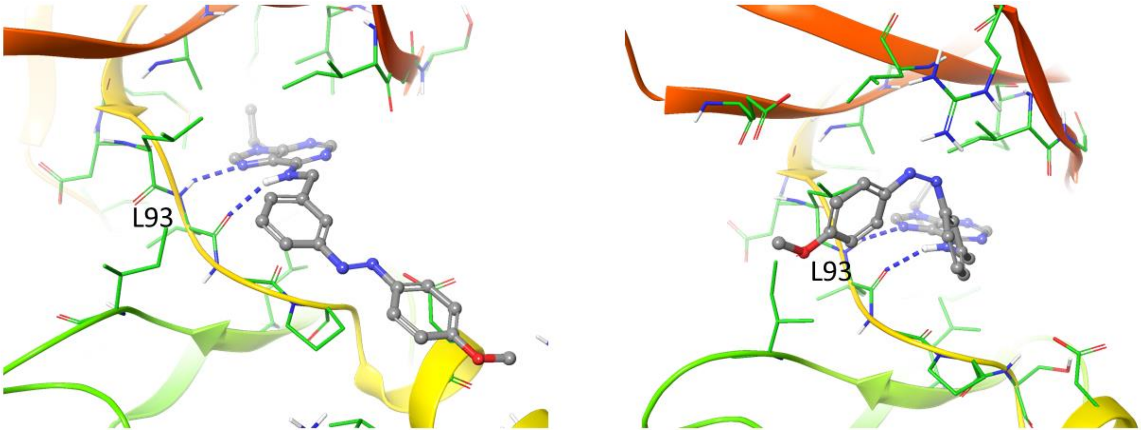

Supplementary Figure 42. Binding mode of *trans*-**6** and *cis*-**6**. Modes are obtained after Glide XP docking and further used for MD simulations.

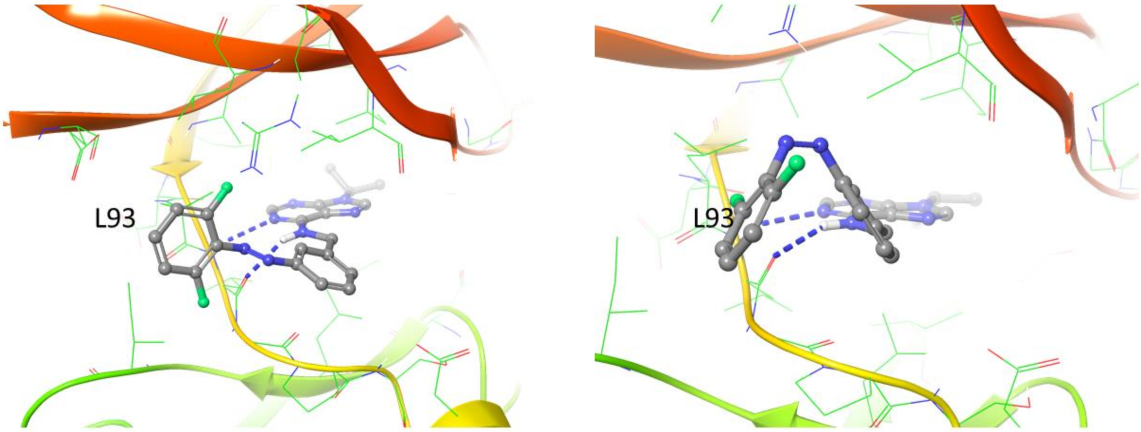

Supplementary Figure 43. Binding mode of *trans*-**7** and *cis*-**7**. Modes are obtained after Glide XP docking and further used for MD simulations.

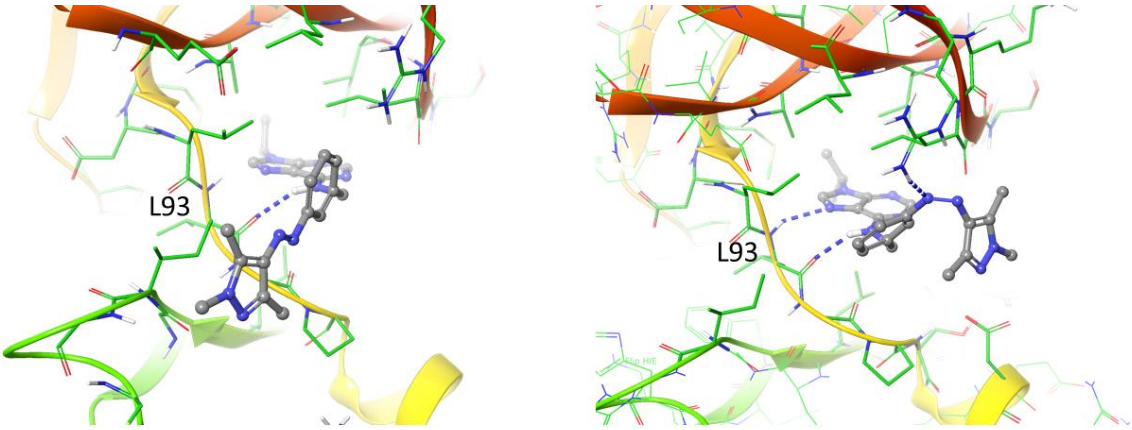

Supplementary Figure 44. Binding mode of *trans*-**8** and *cis*-**8**. Modes are obtained after Glide XP docking and further used for MD simulations.

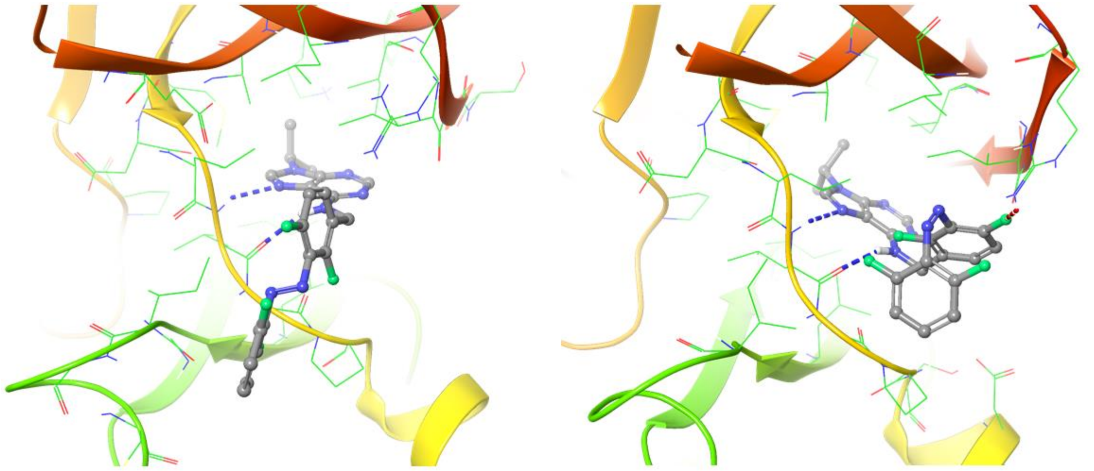

Supplementary Figure 45. Binding mode of *trans*-**9** and *cis*-**9**. Modes are obtained after Glide XP docking and further used for MD simulations.

NMR and HRMS spectra

N-(4-aminobenzyl)-9-isopropyl-9H-purin-6-amine (**13**)

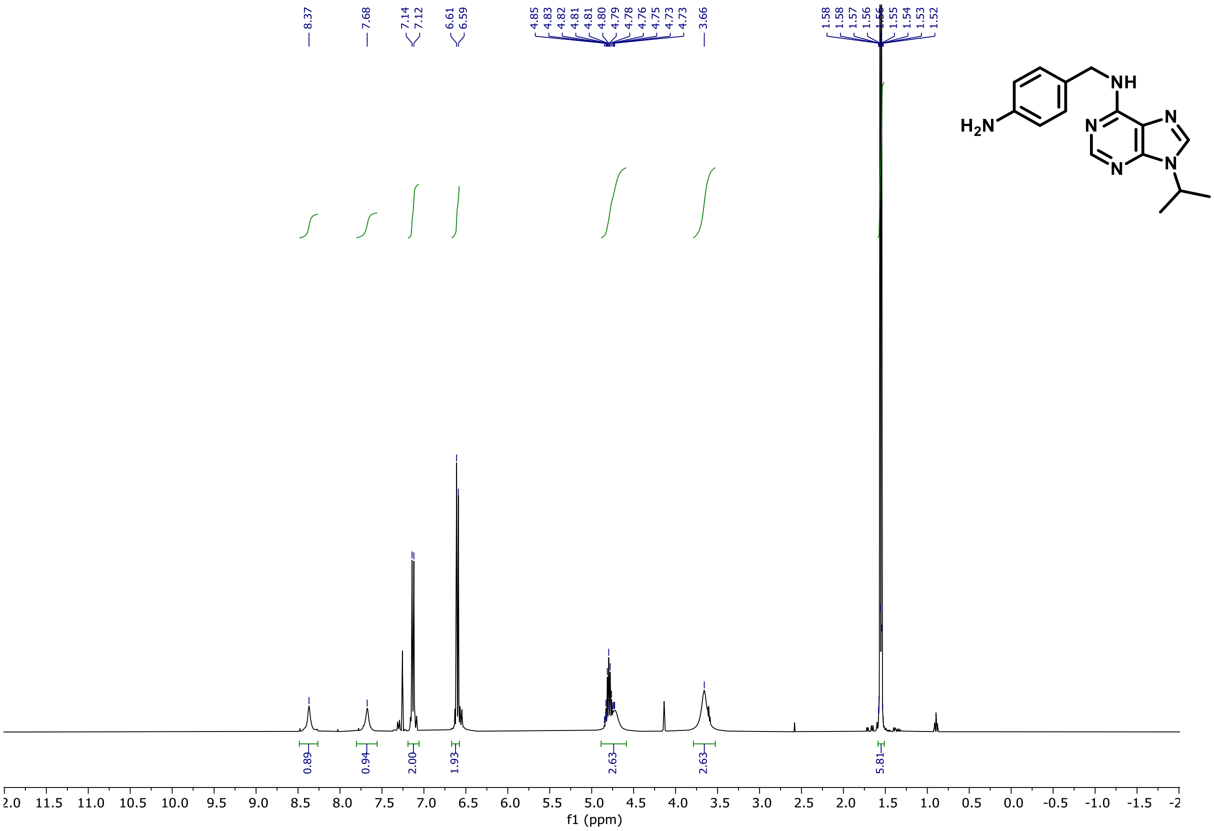

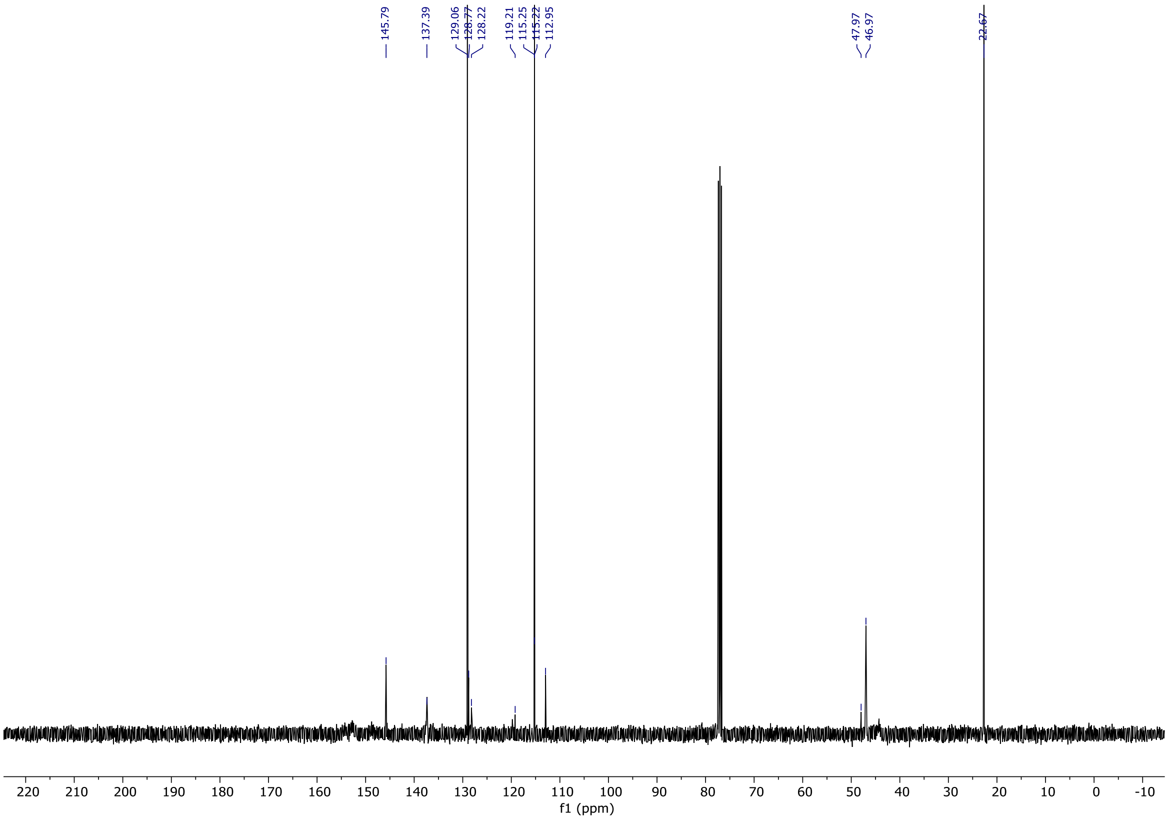

(E)-9-isopropyl-N-(4-(phenyldiazenyl)benzyl)-9H-purin-6-amine (**3**):

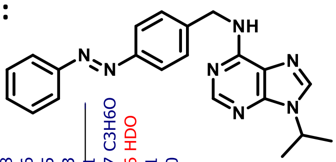

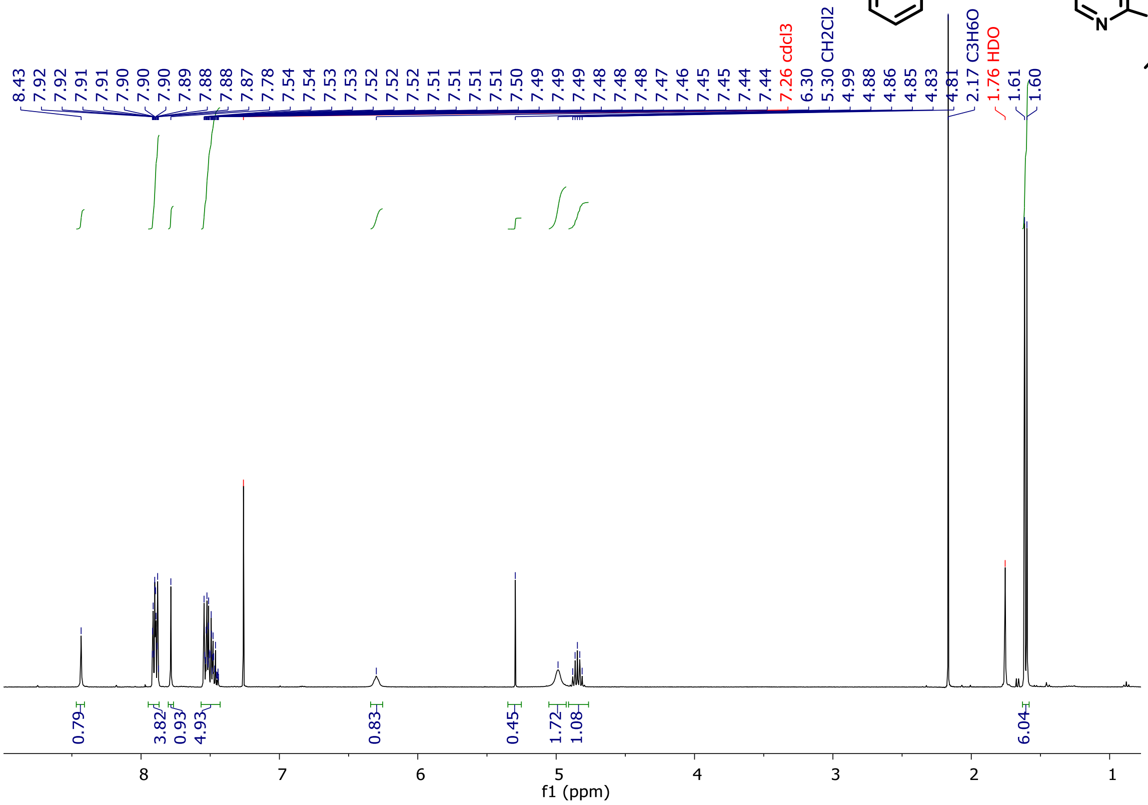

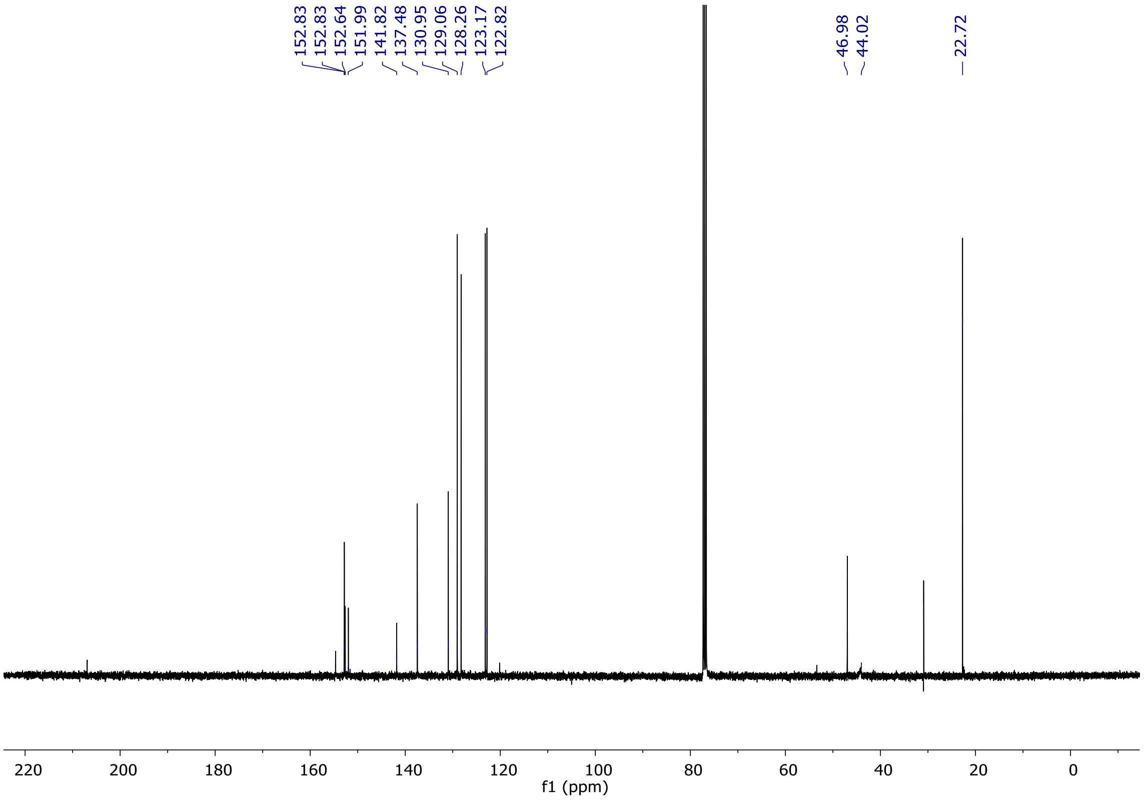

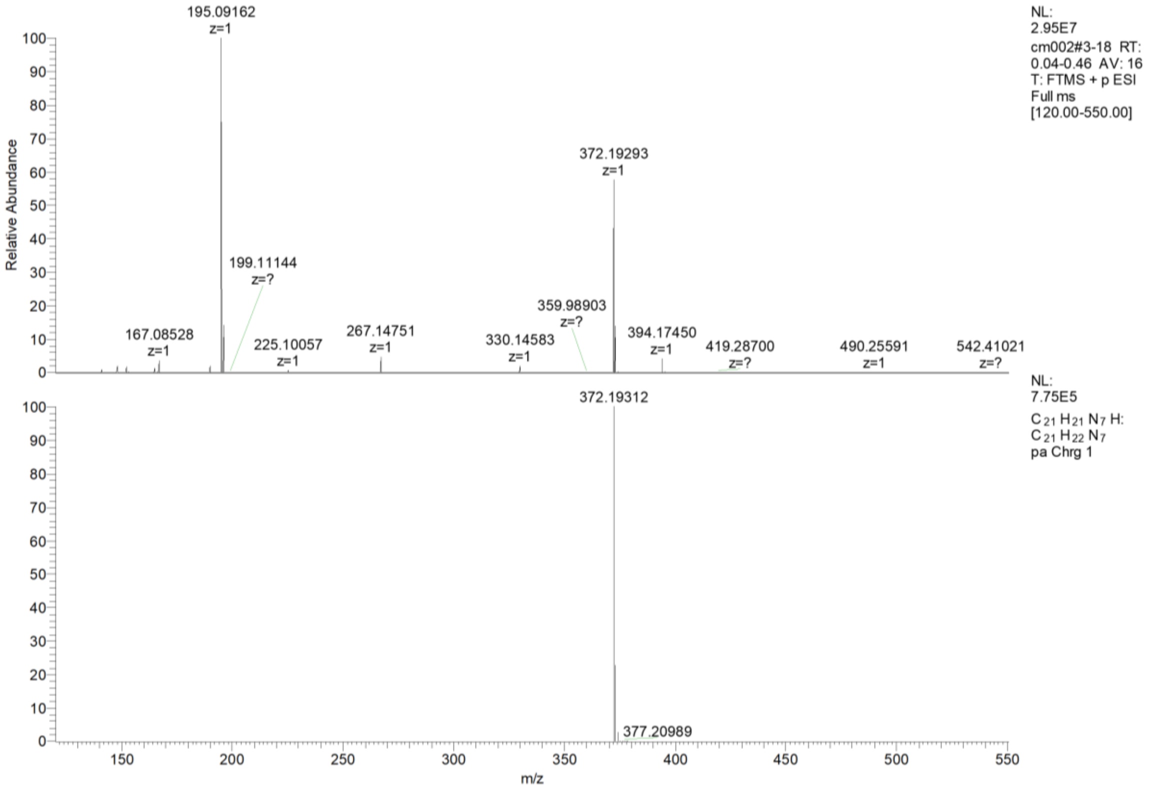
195.09162
z=1
372.19293
z=1
199.11144
z=?
359.98903
z=?
167.08528
z=1
225.10057
z=1
267.14751
z=1
330.14583
z=1
394.17450
z=1
419.28700
z=?
490.25591
z=1
542.41021
z=?
NL:
2.95E7
cm002#3-18 RT:
0.04-0.46 AV: 16
T: FTMS + p ESI
Full ms
[120.00-550.00]
Relative Abundance
372.19312
377.20989
NL:
7.75E5
C 21 H21 N7 H:
C 21 H22 N7
pa Chrg 1
m/z

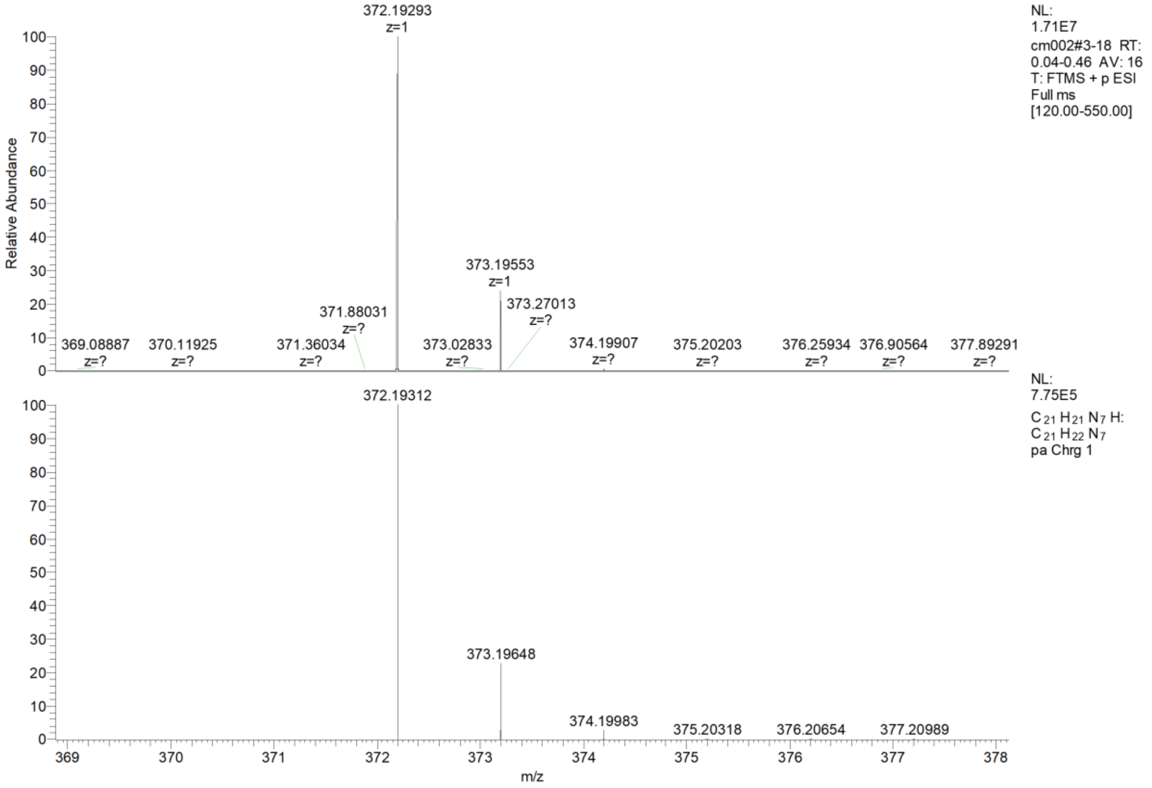
372.19293
z=1
373.19553
z=1
373.27013
z=?
371.88031
z=?
369.08887
z=?
370.11925
z=?
371.36034
z=?
373.02833
z=?
374.19907
z=?
375.20203
z=?
376.25934
z=?
376.90564
z=?
377.89291
z=?
NL:
1.71E7
cm002#3-18 RT:
0.04-0.46 AV: 16
T: FTMS + p ESI
Full ms
[120.00-550.00]
Relative Abundance
372.19312
373.19648
374.19983
375.20318
376.20654
377.20989
NL:
7.75E5
C 21 H21 N7 H:
C 21 H22 N7
pa Chrg 1
m/z

(*E*)-9-isopropyl-N-(3-(phenyldiazenyl)benzyl)-9H-purin-6-amine (**4**)

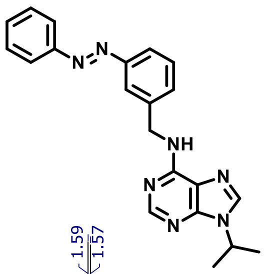

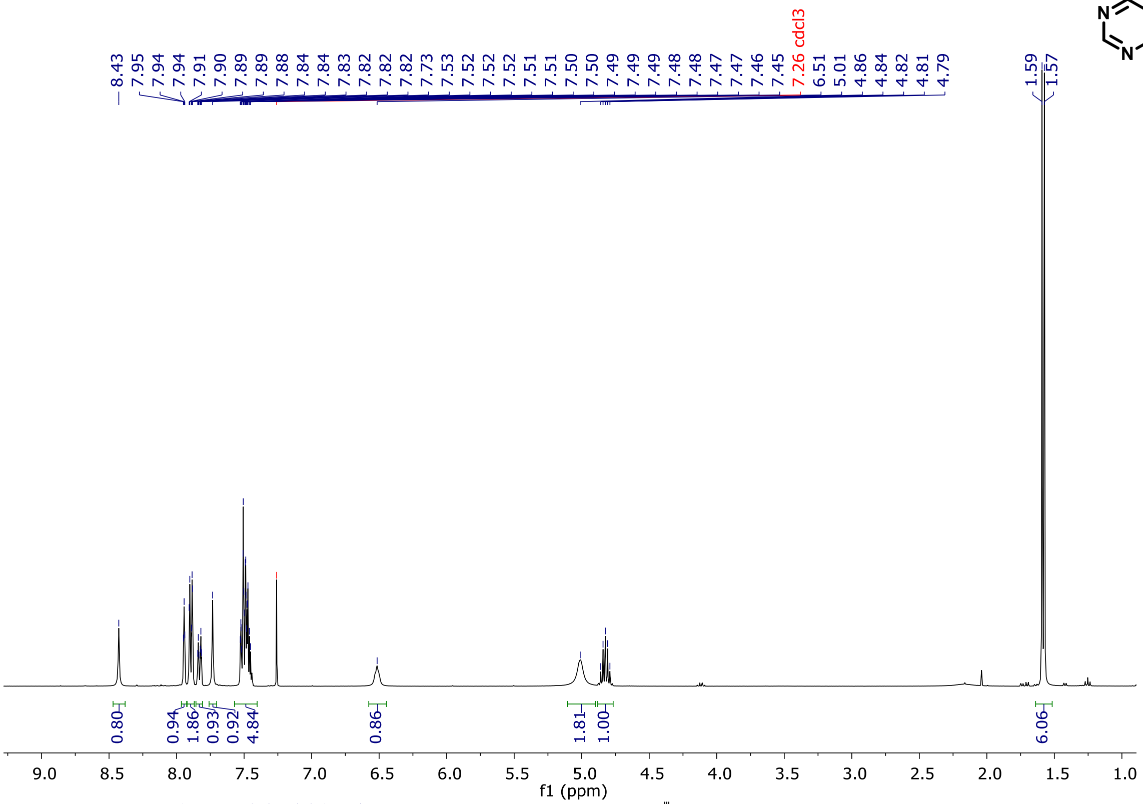

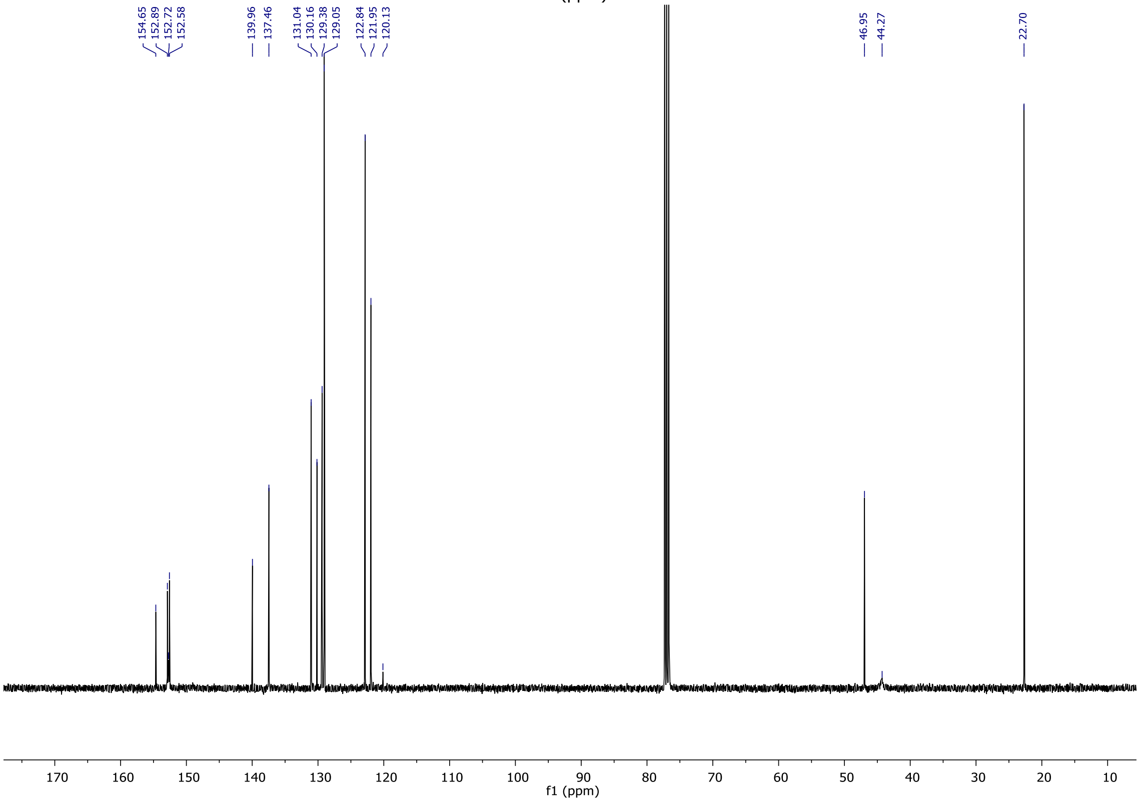

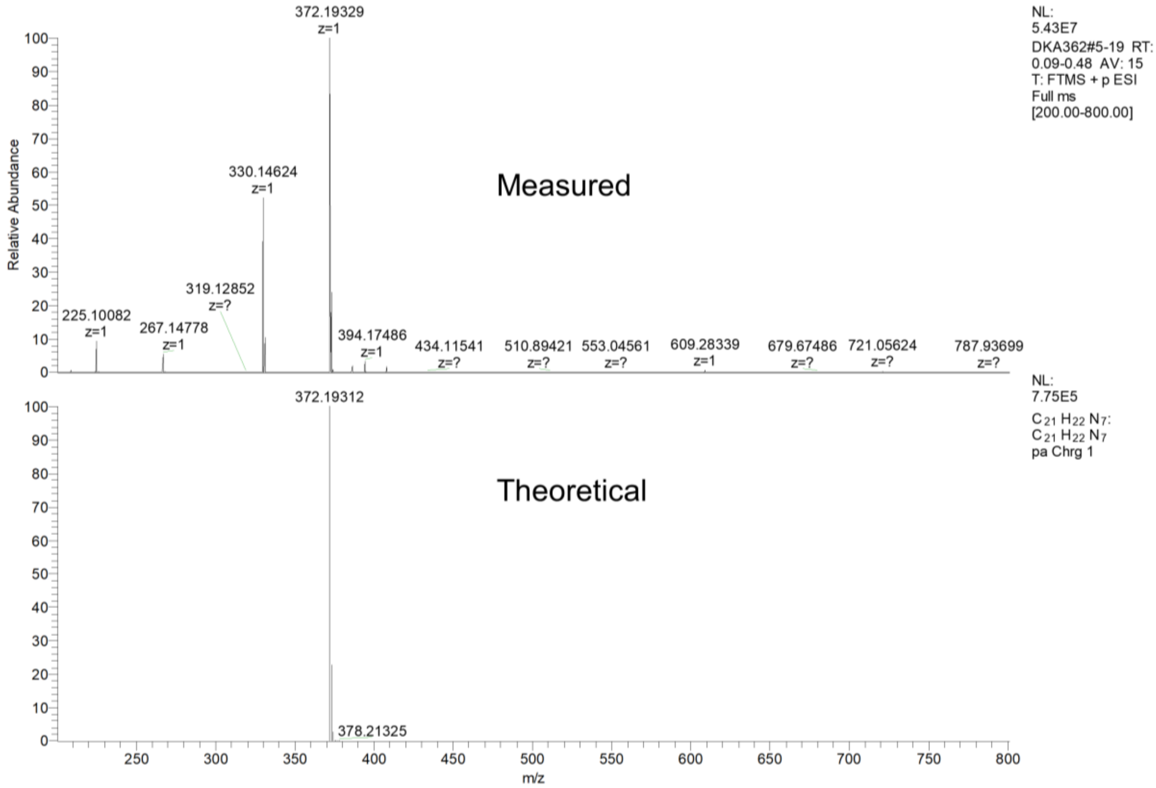
Measured
Theoretical
372.19329
z=1
330.14624
z=1
319.12852
z=?
225.10082
z=1
267.14778
z=1
394.17486
z=1
434.11541
z=?
510.89421
z=?
553.04561
z=?
609.28339
z=1
679.67486
z=?
721.05624
z=?
787.93699
z=?
372.19312
378.21325
NL:
5.43E7
DKA362#5-19 RT:
0.09-0.48 AV: 15
T: FTMS + p ESI
Full ms
[200.00-800.00]
NL:
7.75E5
C 21 H22 N7:
C 21 H22 N7
pa Chrg 1
Relative Abundance
m/z

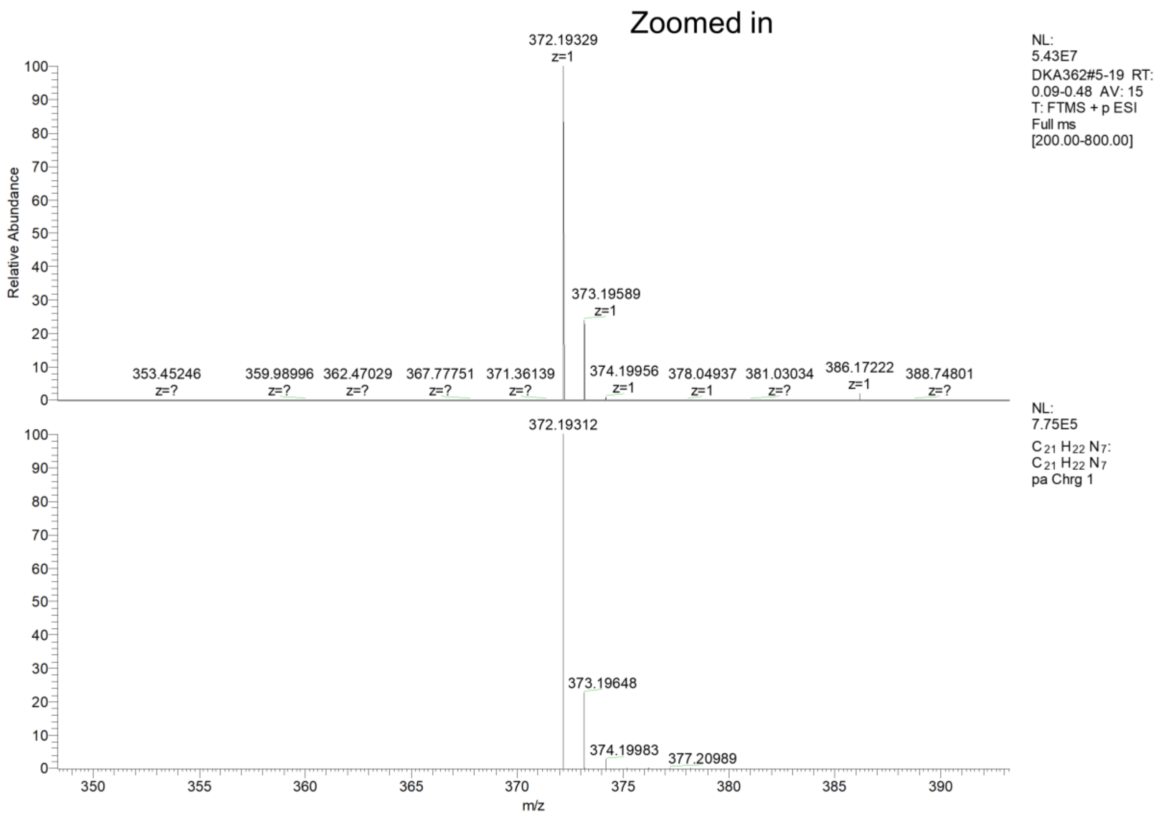
Zoomed in
372.19329
z=1
373.19589
z=1
353.45246
z=?
359.98996
z=?
362.47029
z=?
367.77751
z=?
371.36139
z=?
374.19956
z=1
378.04937
z=1
381.03034
z=?
386.17222
z=1
388.74801
z=?
372.19312
373.19648
374.19983
377.20989
NL:
5.43E7
DKA362#5-19 RT:
0.09-0.48 AV: 15
T: FTMS + p ESI
Full ms
[200.00-800.00]
NL:
7.75E5
C 21 H22 N7:
C 21 H22 N7
pa Chrg 1
Relative Abundance
m/z

(*E*)-9-isopropyl-N-(2-(phenyldiazenyl)benzyl)-9H-purin-6-amine (**5**)

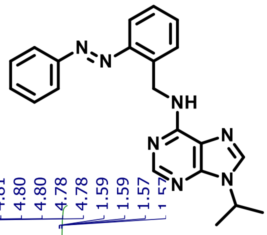

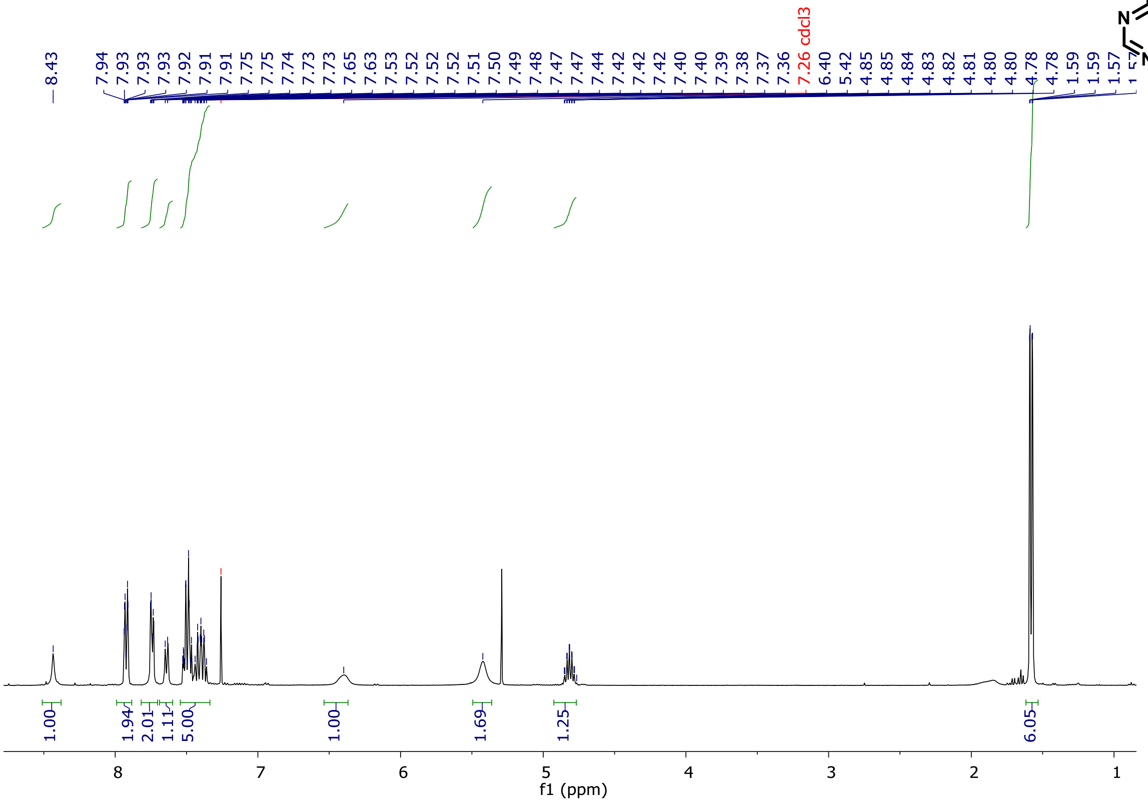

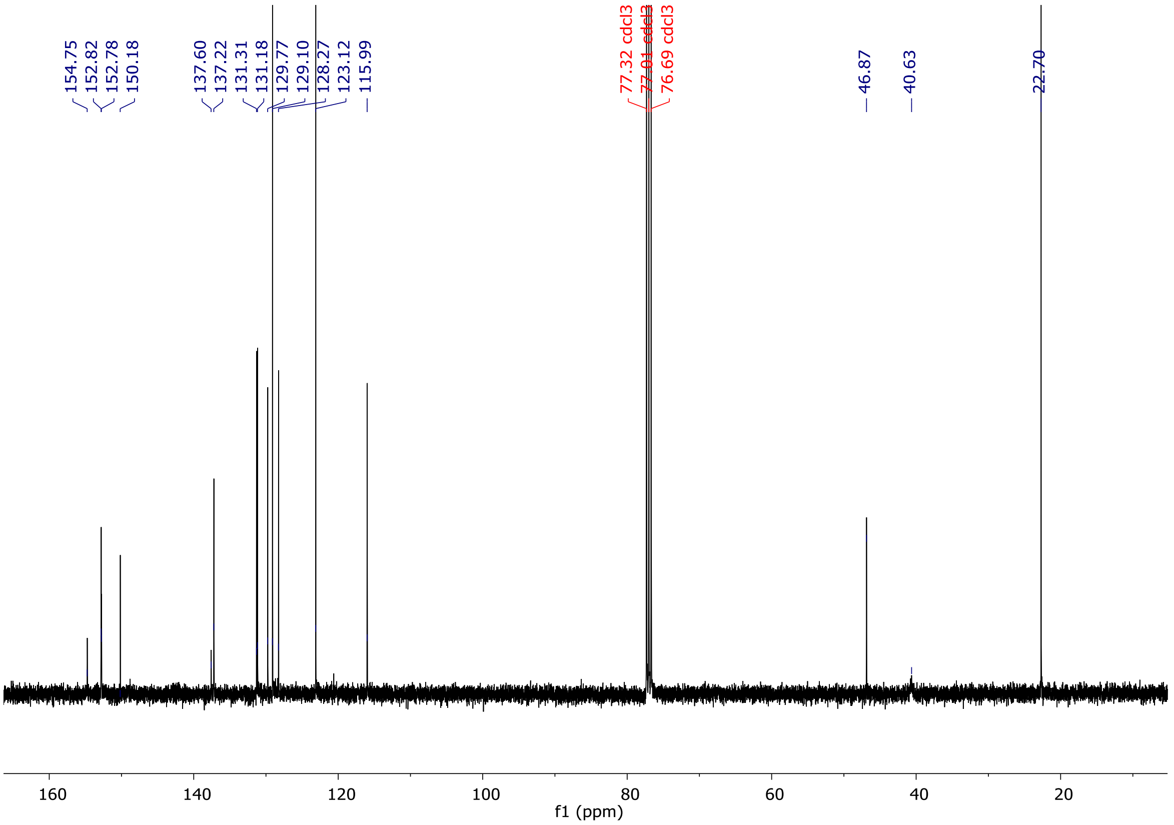

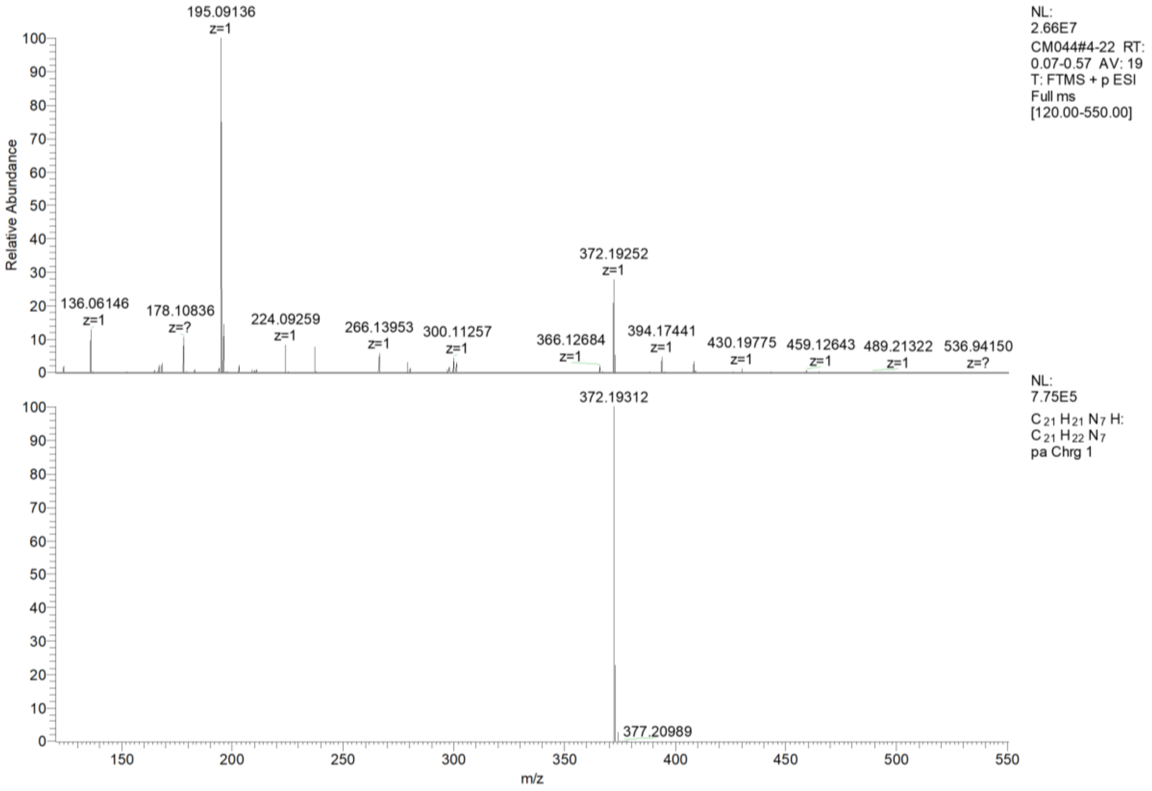
195.09136
z=1
NL:
2.66E7
CM044#4-22 RT:
0.07-0.57 AV: 19
T: FTMS + p ESI
Full ms
[120.00-550.00]
Relative Abundance
136.06146
178.10836
224.09259
266.13953
300.11257
366.12684
372.19252
394.17441
430.19775
459.12643
489.21322
536.94150
NL:
7.75E5
C 21 H 21 N 7 H:
C 21 H 22 N 7
pa Chrg 1
372.19312
377.20989
m/z

Zoomed in

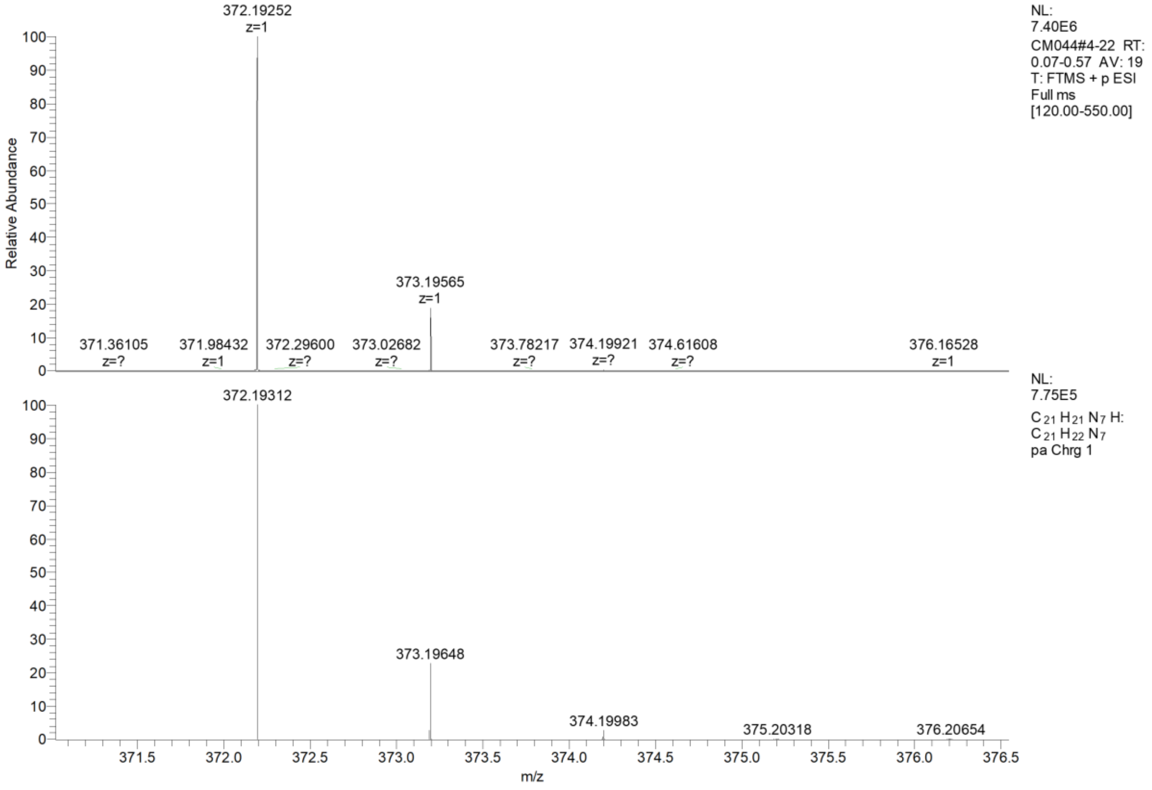
372.19252
z=1
NL:
7.40E6
CM044#4-22 RT:
0.07-0.57 AV: 19
T: FTMS + p ESI
Full ms
[120.00-550.00]
Relative Abundance
373.19565
z=1
371.36105
371.98432
372.29600
373.02682
373.78217
374.19921
374.61608
376.16528
NL:
7.75E5
C 21 H 21 N 7 H:
C 21 H 22 N 7
pa Chrg 1
372.19312
373.19648
374.19983
375.20318
376.20654
m/z

N-(3-aminobenzyl)-9-isopropyl-9H-purin-6-amine (**12**)

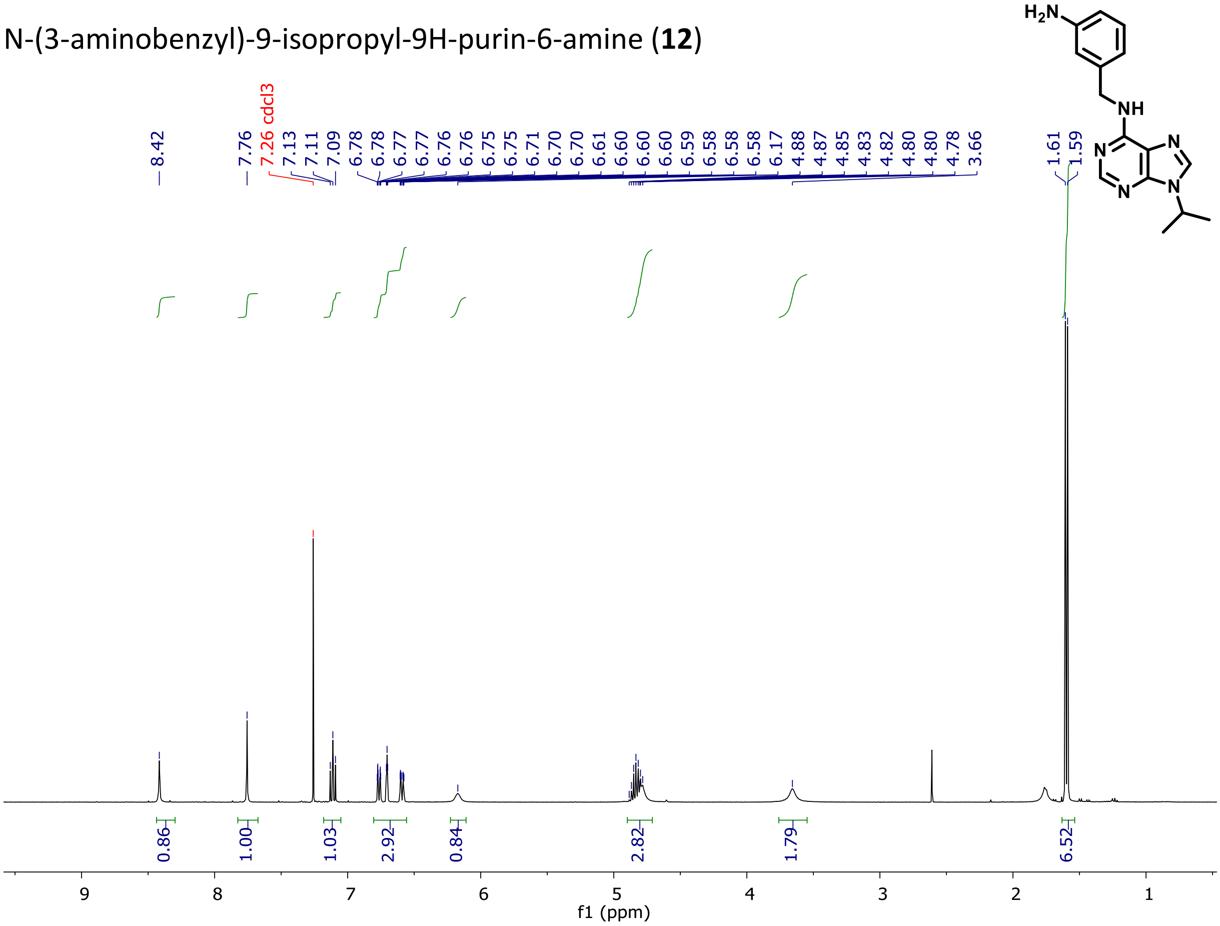

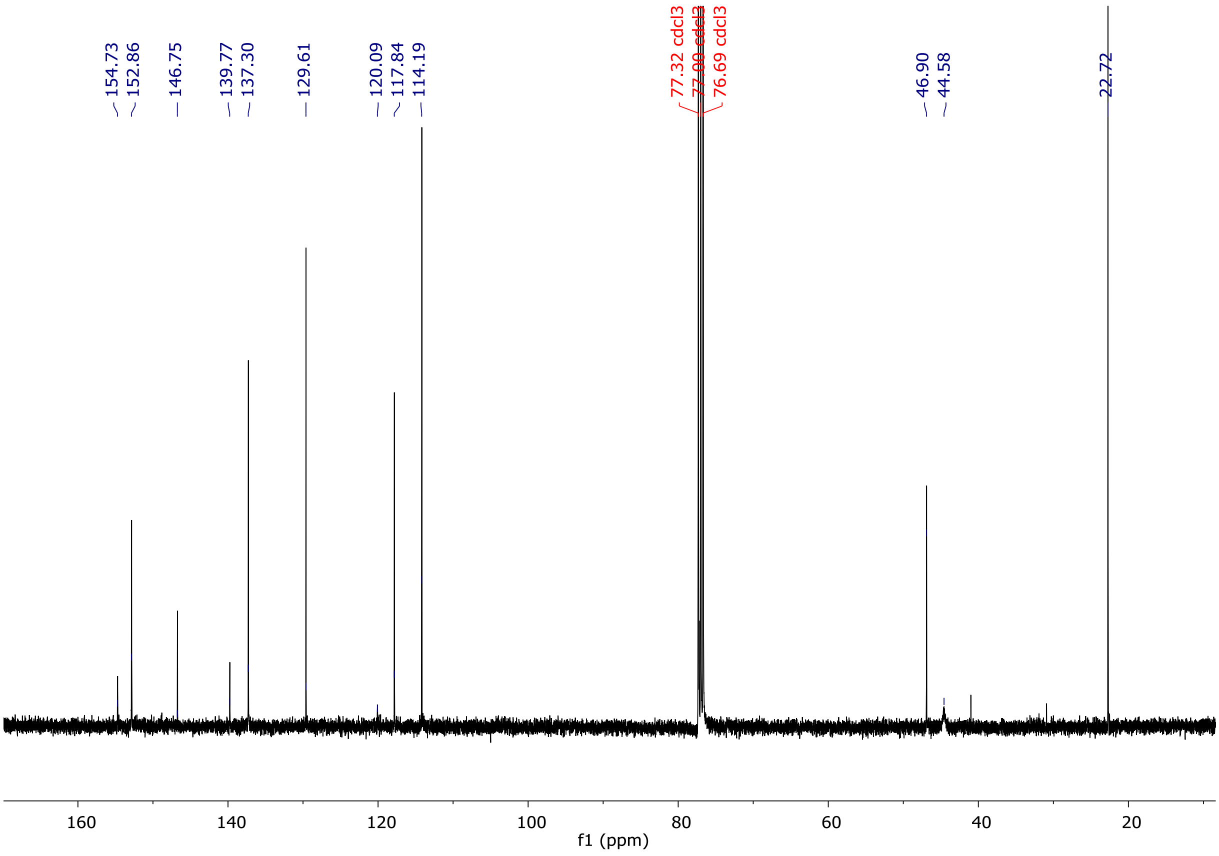

Methyl (*E*)-3-((4-hydroxyphenyl)diazenyl)benzoate (**15**)

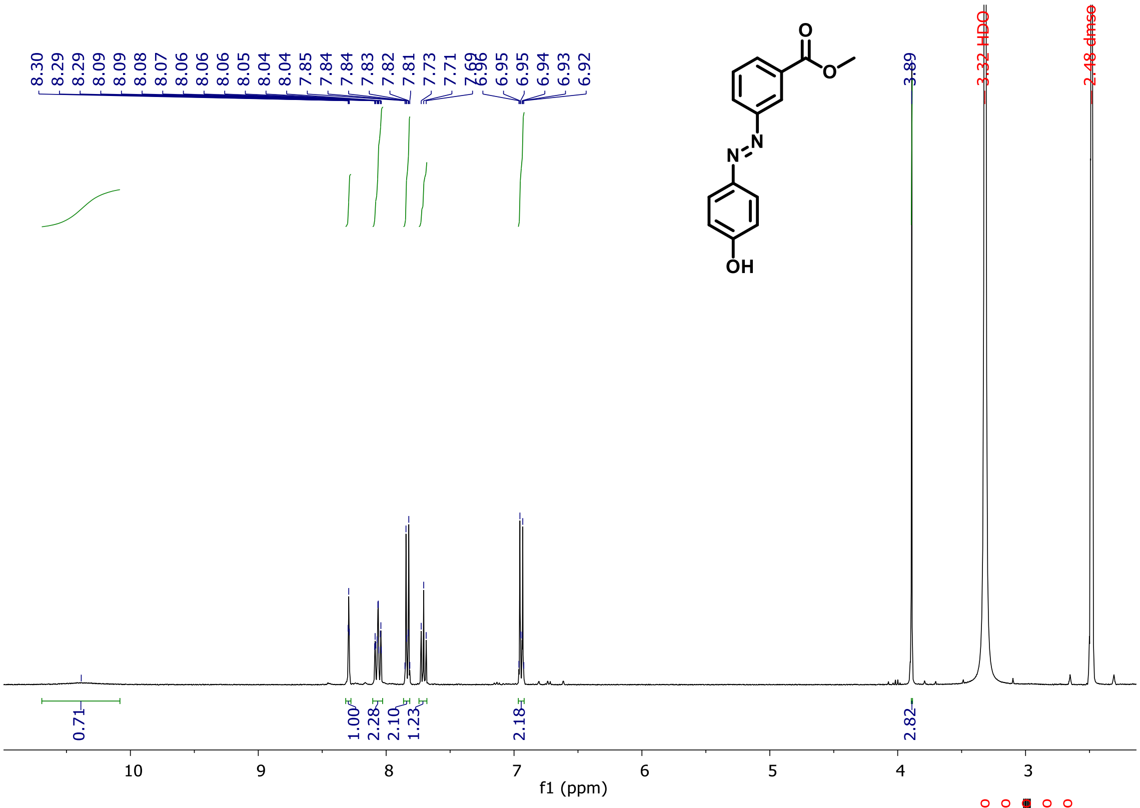

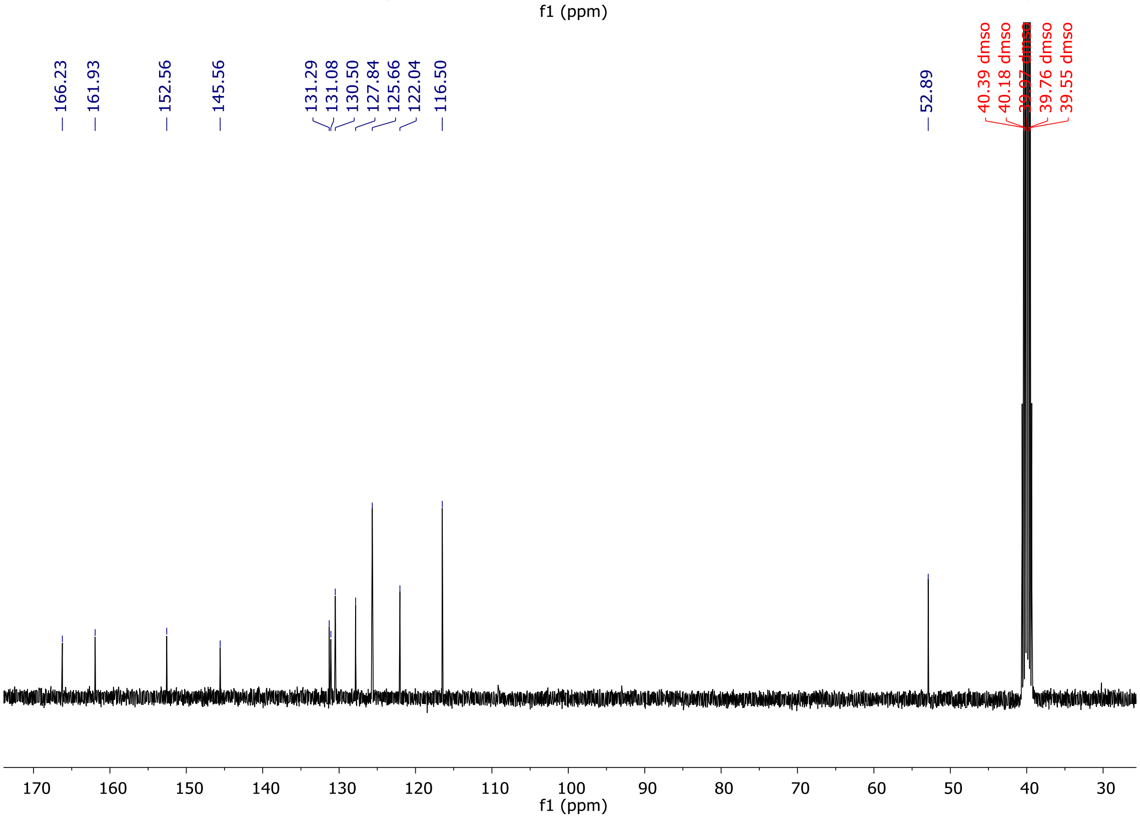

ESI pos

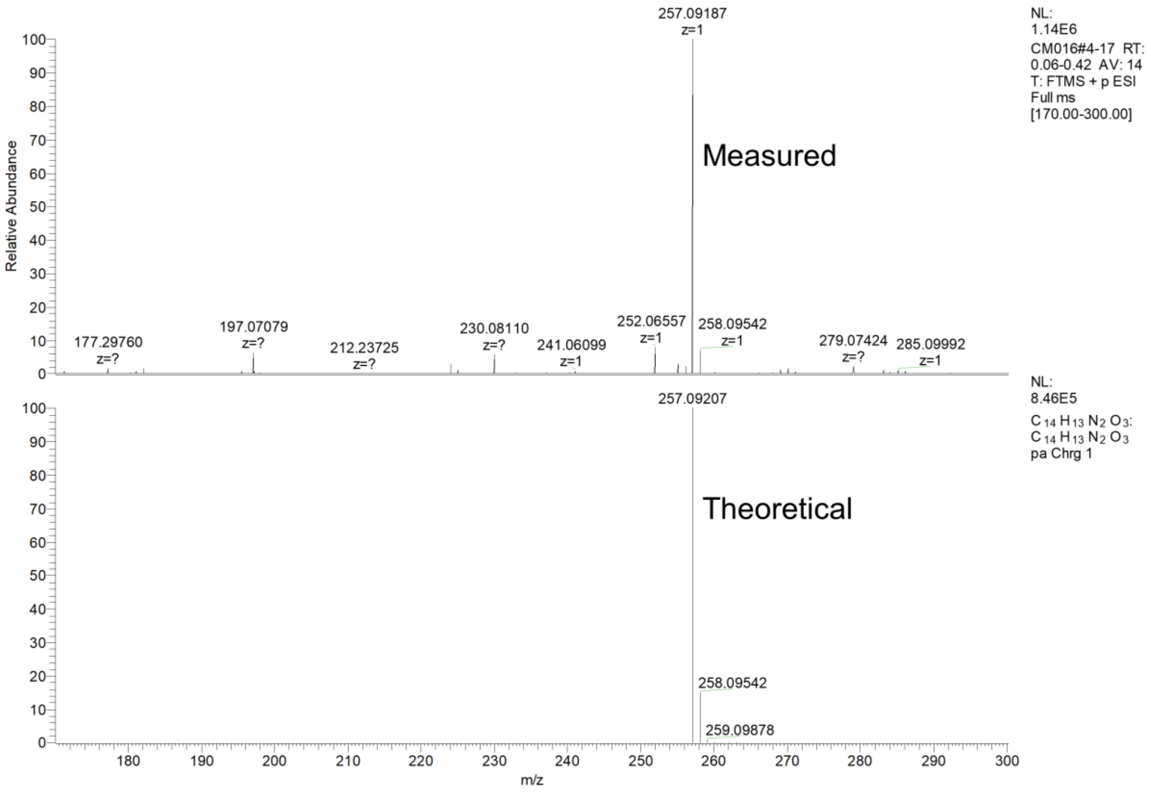

Zoomed in

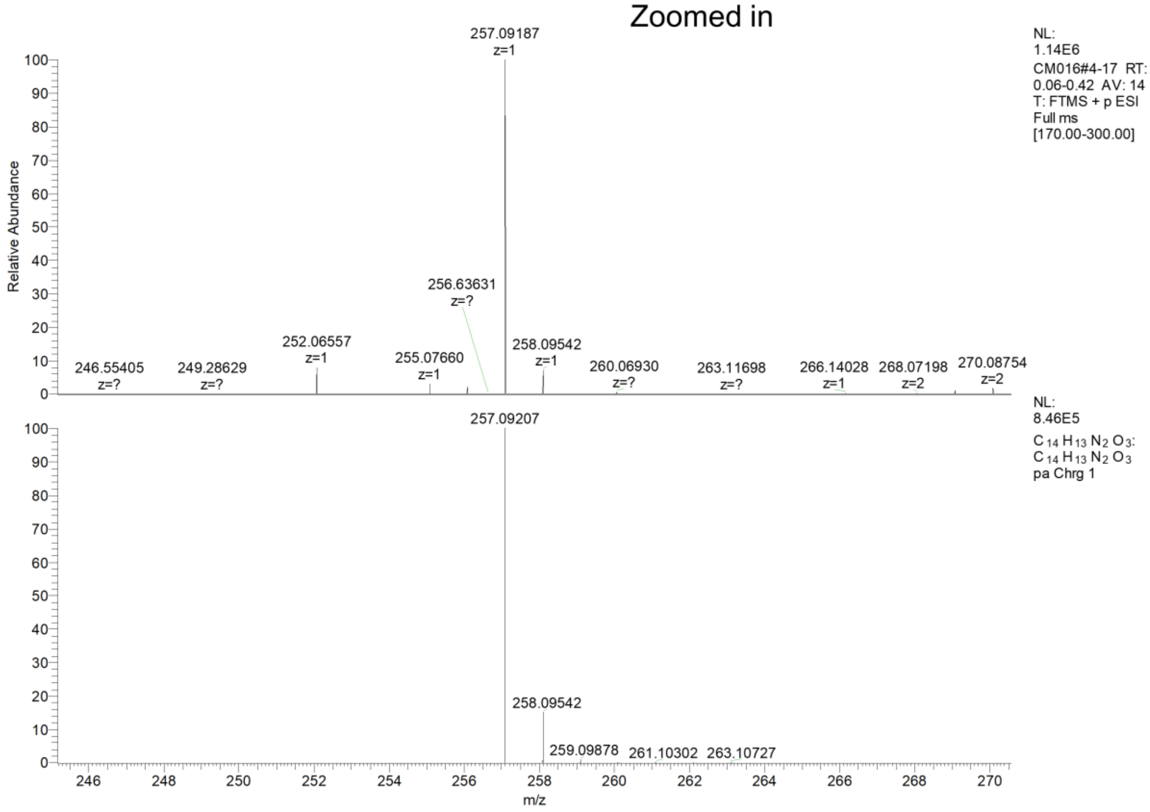

Methyl (*E*)-3-((4-methoxyphenyl)diazenyl)benzoate (**16**)

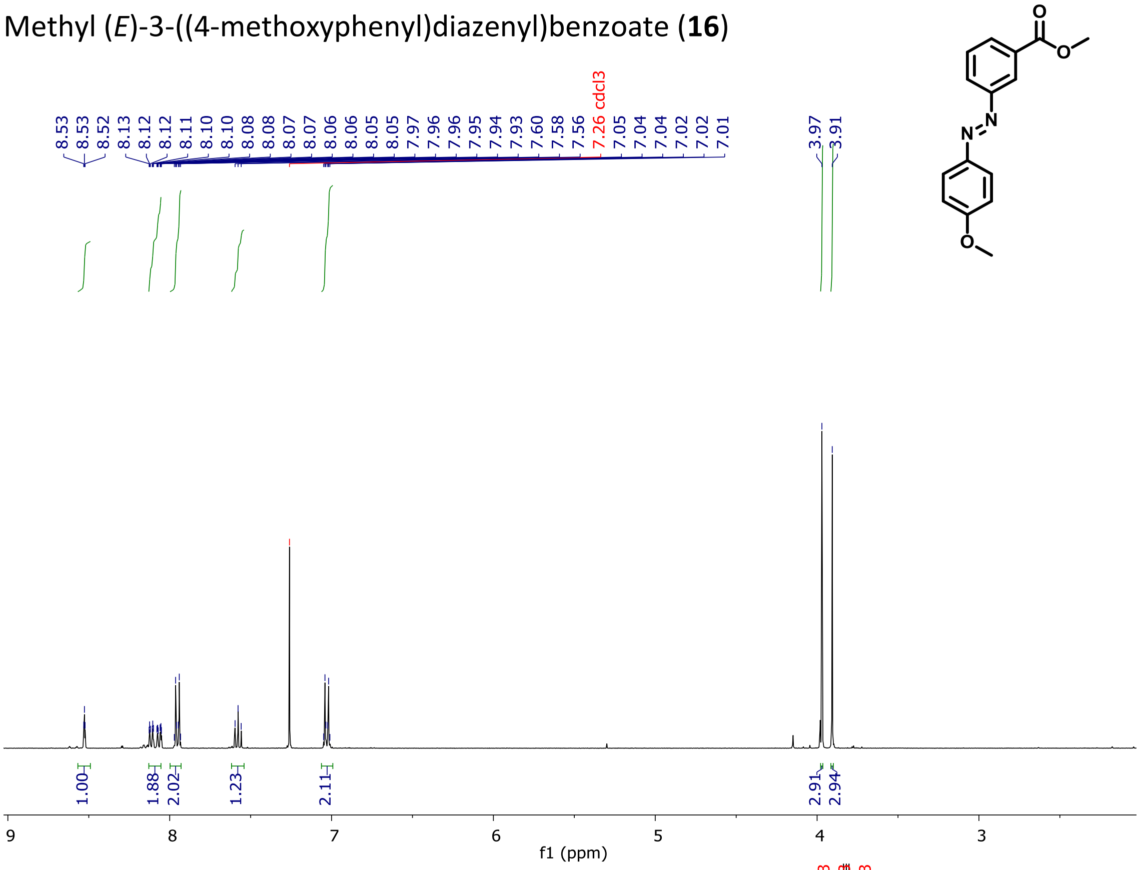

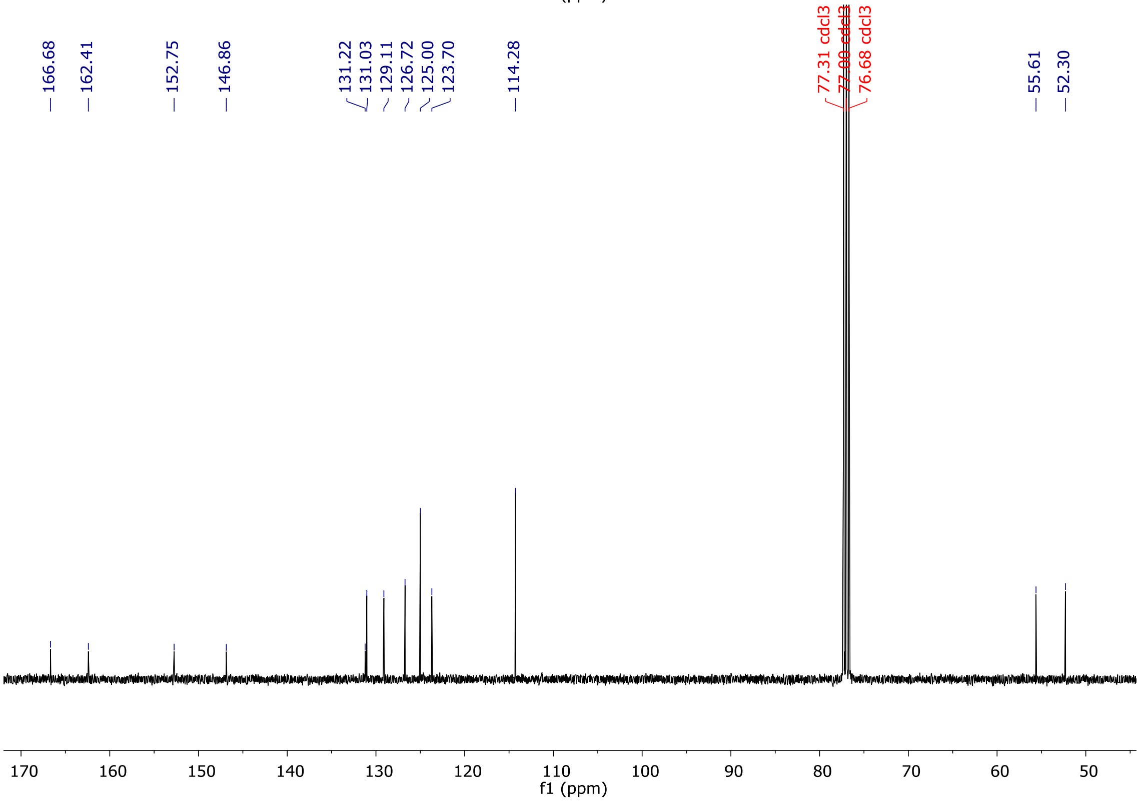

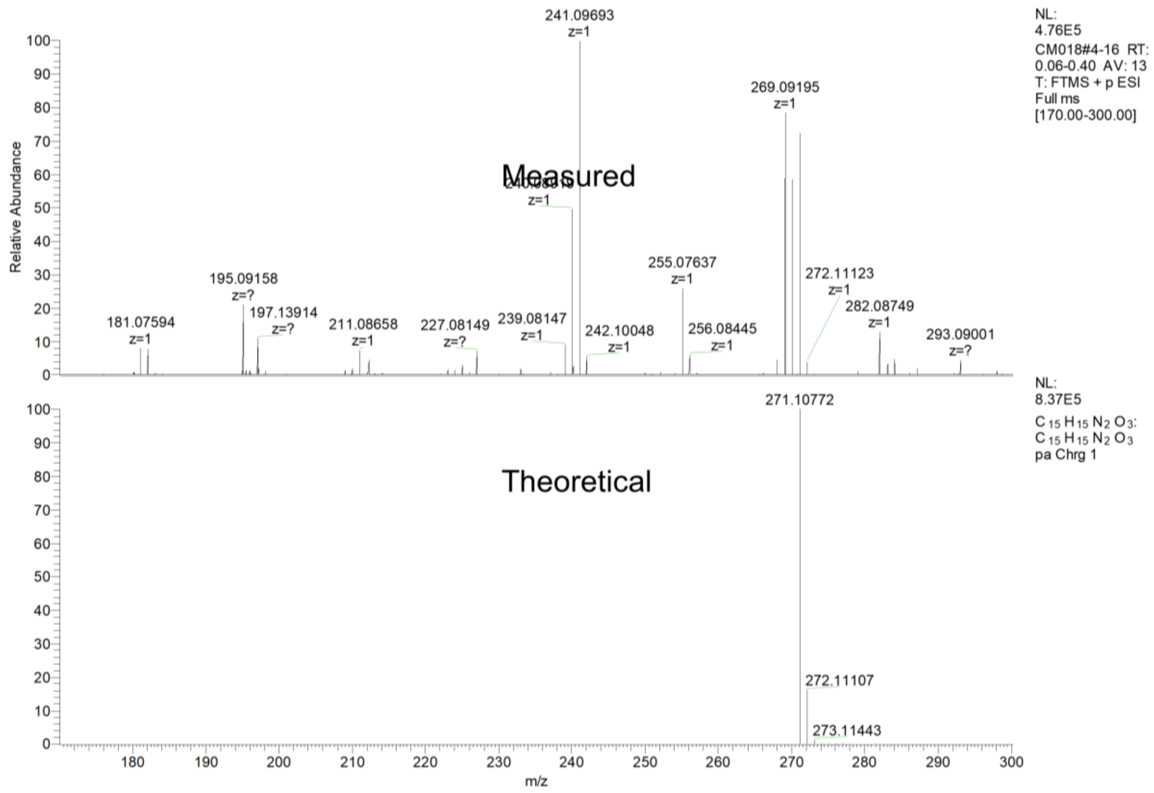
Measured
Theoretical
241.09693
269.09195
195.09158
197.13914
181.07594
211.08658
227.08149
239.08147
242.10048
255.07637
256.08445
272.11123
282.08749
293.09001
271.10772
272.11107
273.11443
NL: 4.76E5
CM018#4-16 RT: 0.06-0.40 AV: 13
T: FTMS + p ESI Full ms [170.00-300.00]
NL: 8.37E5
C 15 H 15 N 2 O 3: C 15 H 15 N 2 O 3
pa Chrg 1
Relative Abundance
m/z

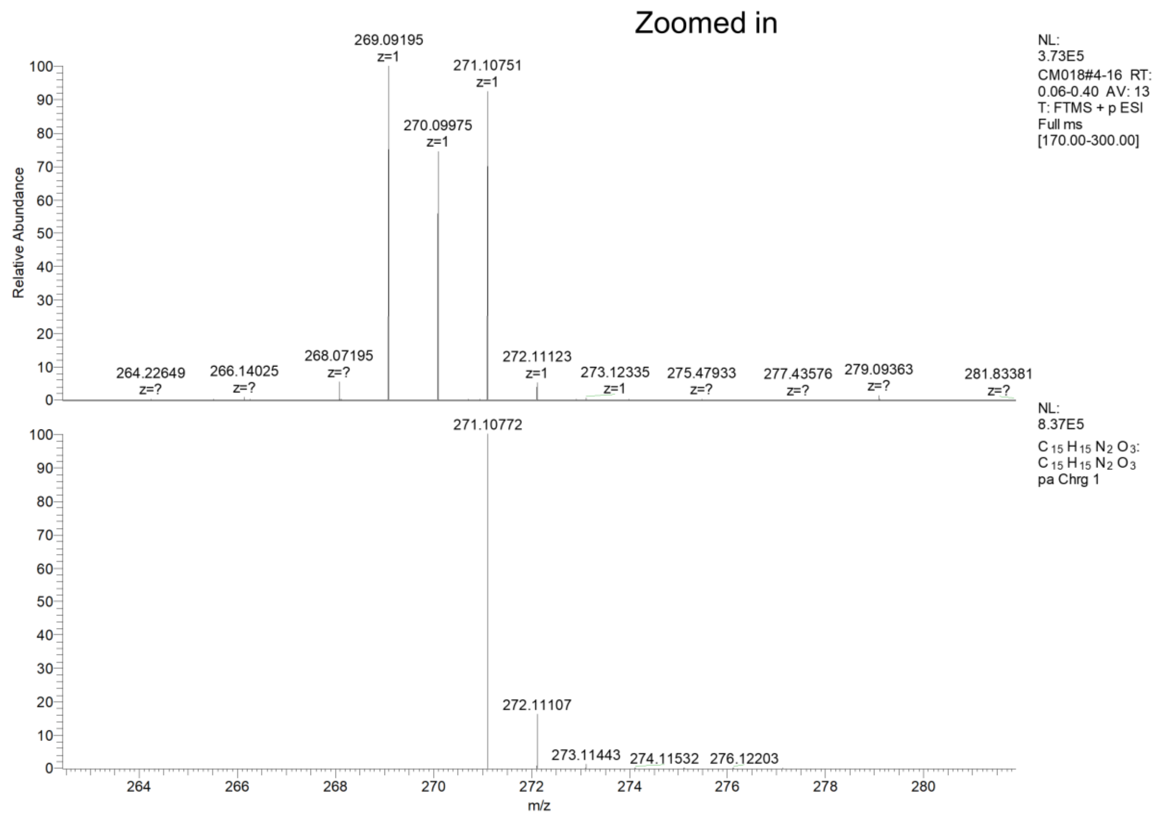
Zoomed in
269.09195
271.10751
270.09975
264.22649
266.14025
268.07195
272.11123
273.12335
275.47933
277.43576
279.09363
281.83381
271.10772
272.11107
273.11443
274.11532
276.12203
NL: 3.73E5
CM018#4-16 RT: 0.06-0.40 AV: 13
T: FTMS + p ESI Full ms [170.00-300.00]
NL: 8.37E5
C 15 H 15 N 2 O 3: C 15 H 15 N 2 O 3
pa Chrg 1
Relative Abundance
m/z

(*E*)-(3-((4-methoxyphenyl)diazenyl)phenyl)methanol (**17**)

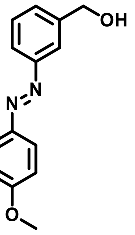

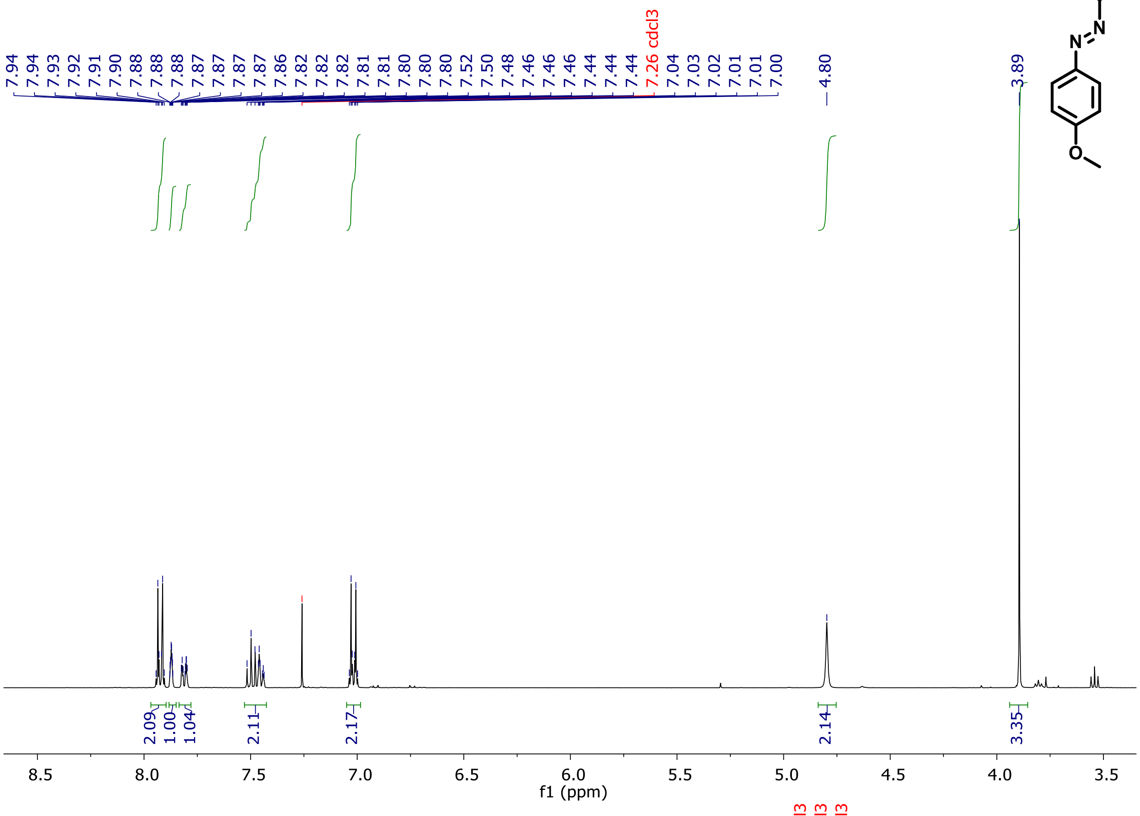

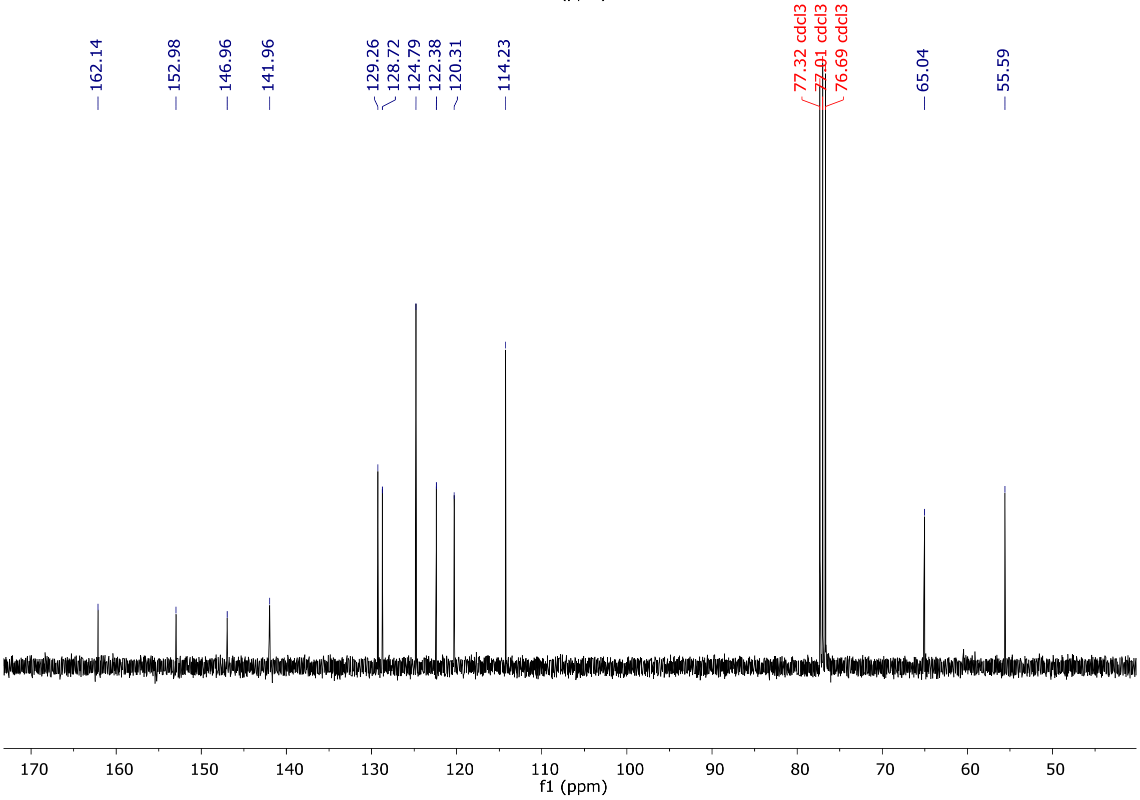

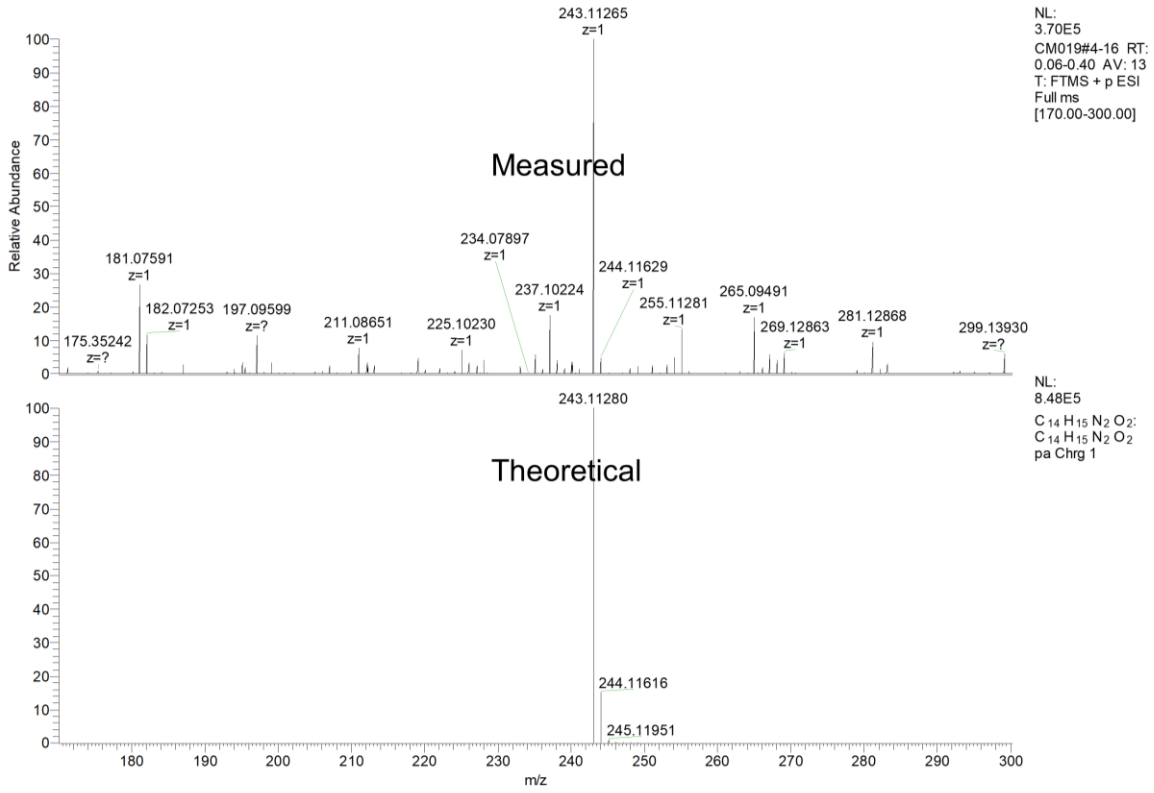

Measured
Theoretical
243.11265 z=1
181.07591 z=1
182.07253 z=1
197.09599 z=?
175.35242 z=?
211.08651 z=1
225.10230 z=1
234.07897 z=1
237.10224 z=1
244.11629 z=1
255.11281 z=1
265.09491 z=1
269.12863 z=1
281.12868 z=1
299.13930 z=?
243.11280
244.11616
245.11951
Relative Abundance
m/z
NL: 3.70E5
CM019#4-16 RT: 0.06-0.40 AV: 13
T: FTMS + p ESI Full ms [170.00-300.00]
NL: 8.48E5
C 14 H 15 N 2 O 2:
C 14 H 15 N 2 O 2
pa Chrg 1

Zoomed in

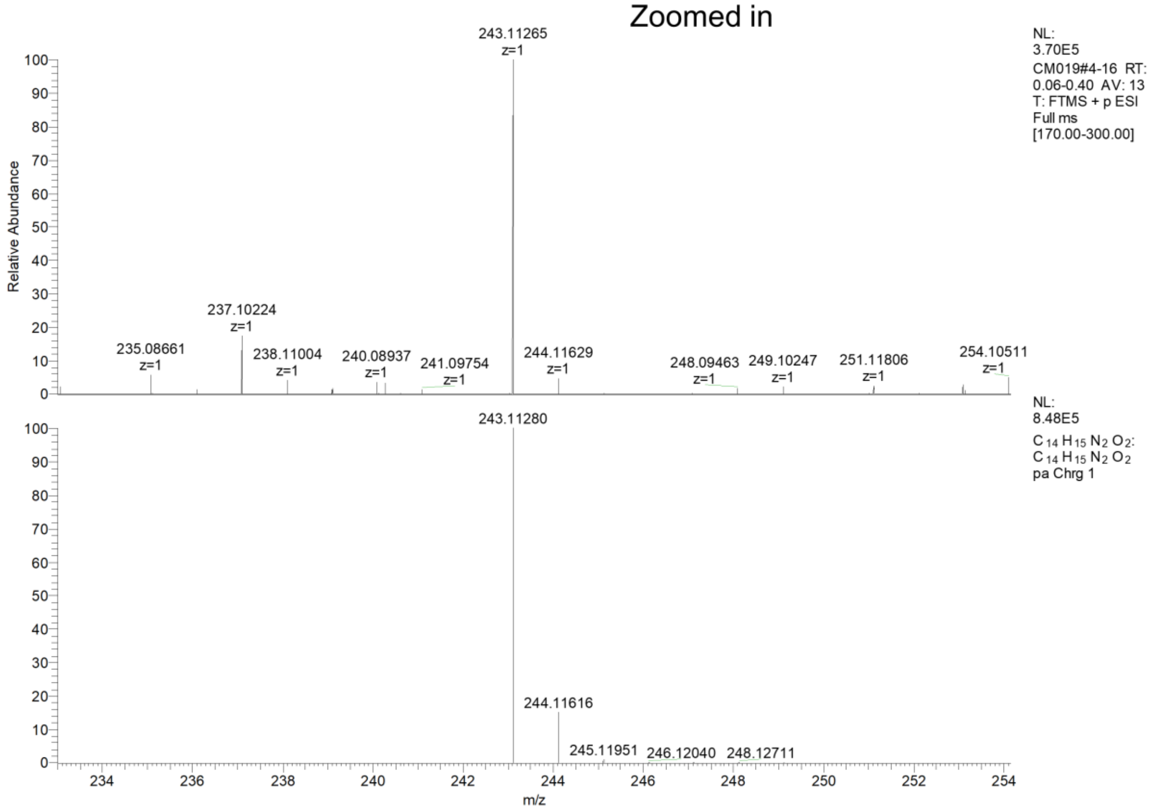

243.11265 z=1
235.08661 z=1
237.10224 z=1
238.11004 z=1
240.08937 z=1
241.09754 z=1
244.11629 z=1
248.09463 z=1
249.10247 z=1
251.11806 z=1
254.10511 z=1
243.11280
244.11616
245.11951
246.12040
248.12711
Relative Abundance
m/z
NL: 3.70E5
CM019#4-16 RT: 0.06-0.40 AV: 13
T: FTMS + p ESI Full ms [170.00-300.00]
NL: 8.48E5
C 14 H 15 N 2 O 2:
C 14 H 15 N 2 O 2
pa Chrg 1

(*E*)-1-(3-(bromomethyl)phenyl)-2-(4-methoxyphenyl)diazene (**18**)

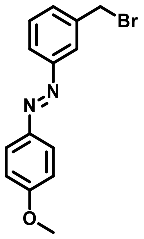

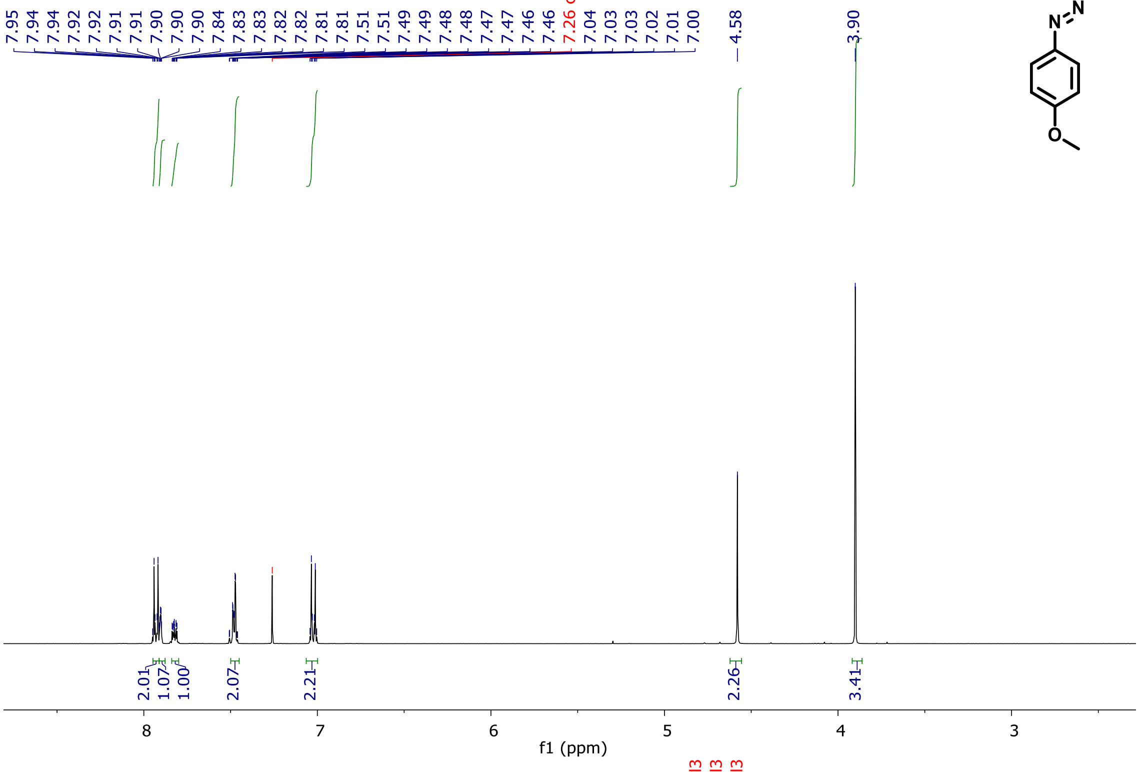

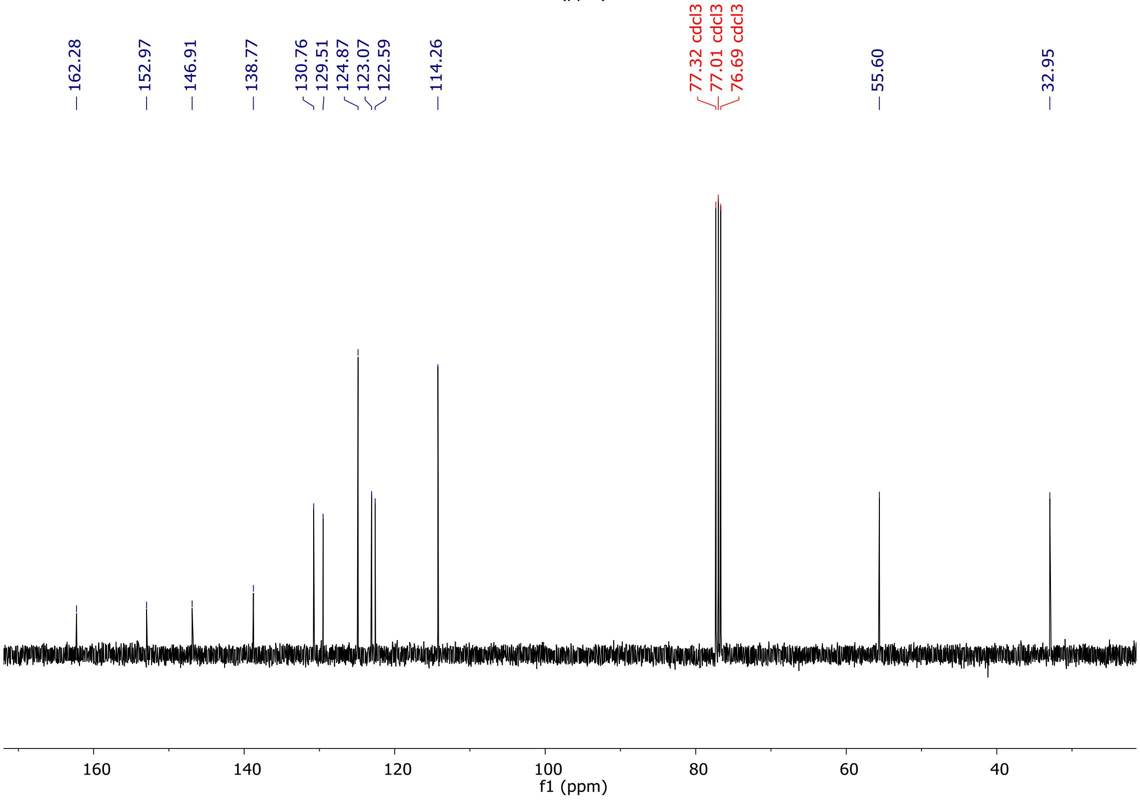

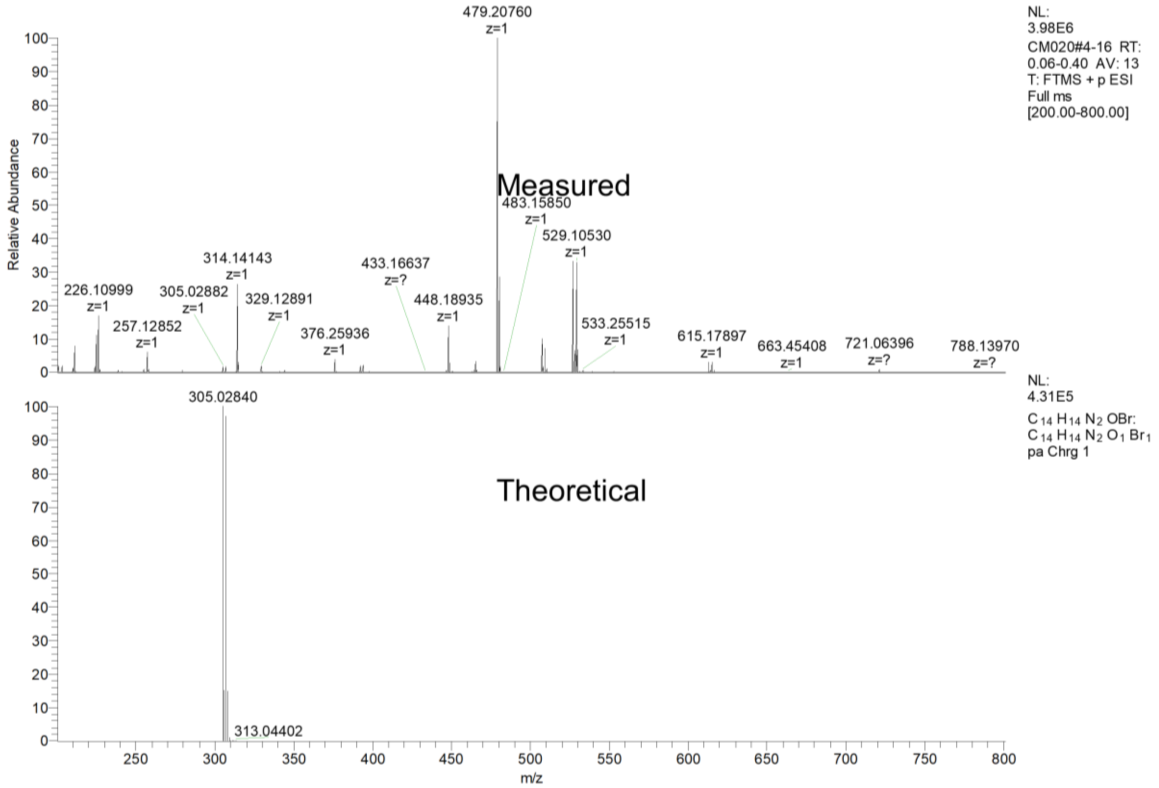
Measured
Theoretical
Relative Abundance
m/z
479.20760 z=1
483.15850 z=1
529.10530 z=1
314.14143 z=1
433.16637 z=?
226.10999 z=1
305.02882 z=1
329.12891 z=1
448.18935 z=1
257.12852 z=1
376.25936 z=1
533.25515 z=1
615.17897 z=1
663.45408 z=1
721.06396 z=?
788.13970 z=?
305.02840
313.04402
NL: 3.98E6
CM020#4-16 RT: 0.06-0.40 AV: 13
T: FTMS + p ESI Full ms [200.00-800.00]
NL: 4.31E5
C 14 H 14 N 2 OBr: C 14 H 14 N 2 O 1 Br 1
pa Chrg 1

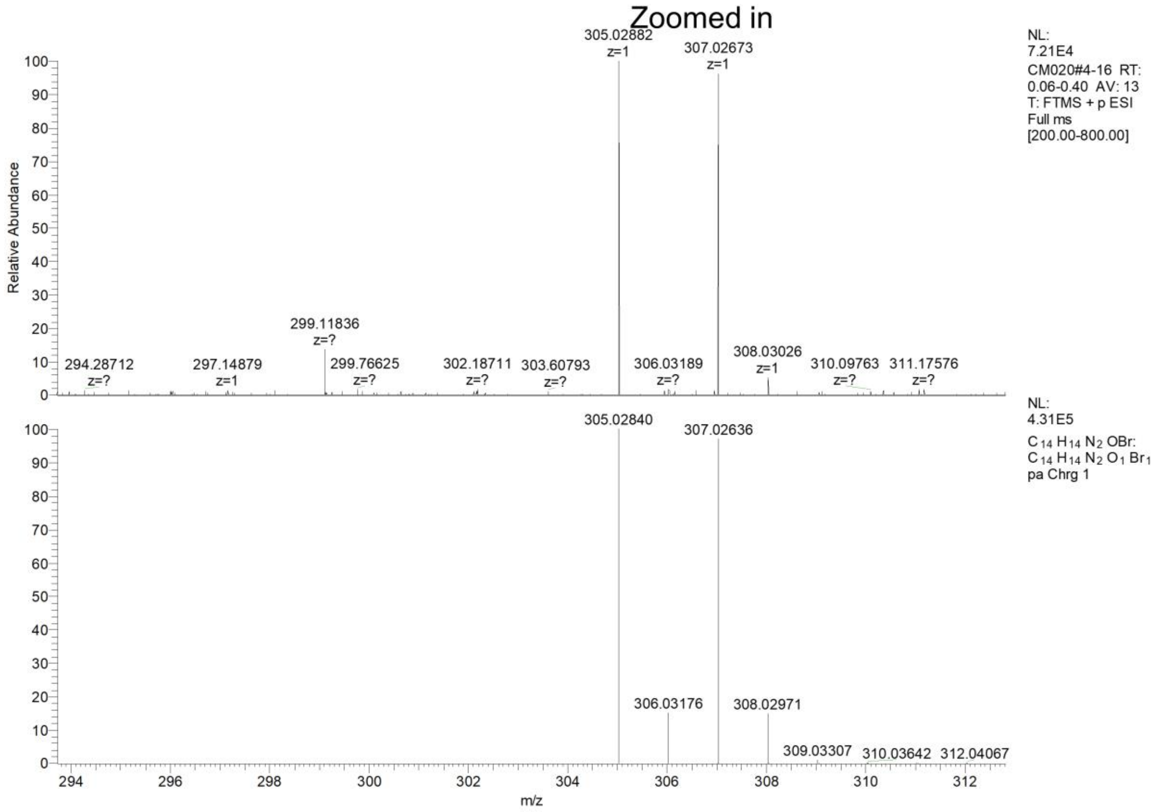
Zoomed in
Relative Abundance
m/z
305.02882 z=1
307.02673 z=1
299.11836 z=?
294.28712 z=?
297.14879 z=1
299.76625 z=?
302.18711 z=?
303.60793 z=?
306.03189 z=?
308.03026 z=1
310.09763 z=?
311.17576 z=?
305.02840
307.02636
306.03176
308.02971
309.03307
310.03642
312.04067
NL: 7.21E4
CM020#4-16 RT: 0.06-0.40 AV: 13
T: FTMS + p ESI Full ms [200.00-800.00]
NL: 4.31E5
C 14 H 14 N 2 OBr: C 14 H 14 N 2 O 1 Br 1
pa Chrg 1

(*E*)-(3-((4-methoxyphenyl)diazenyl)phenyl)methanamine (**19**)

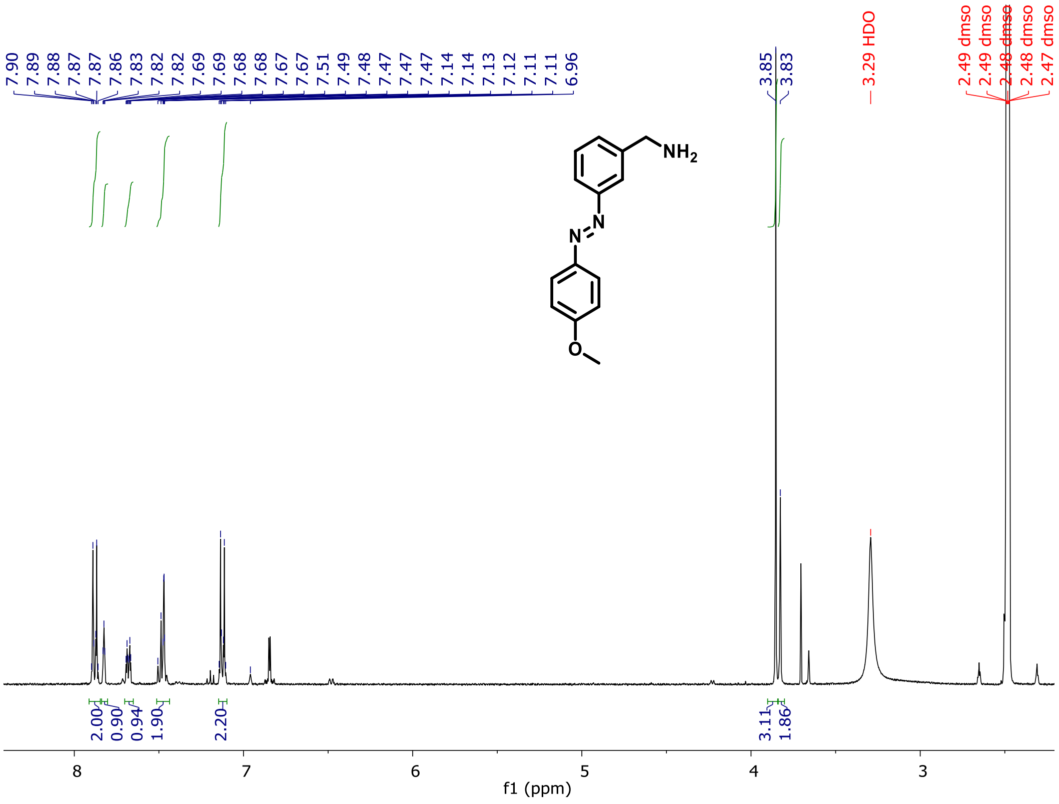

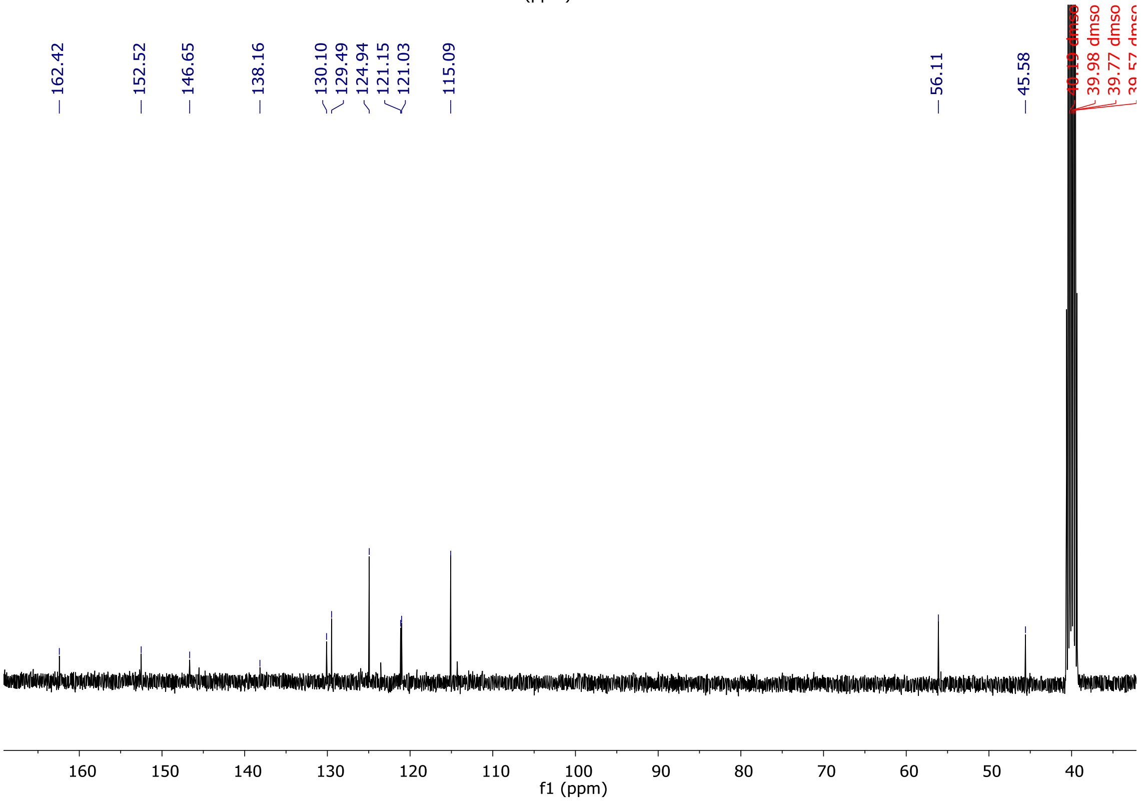

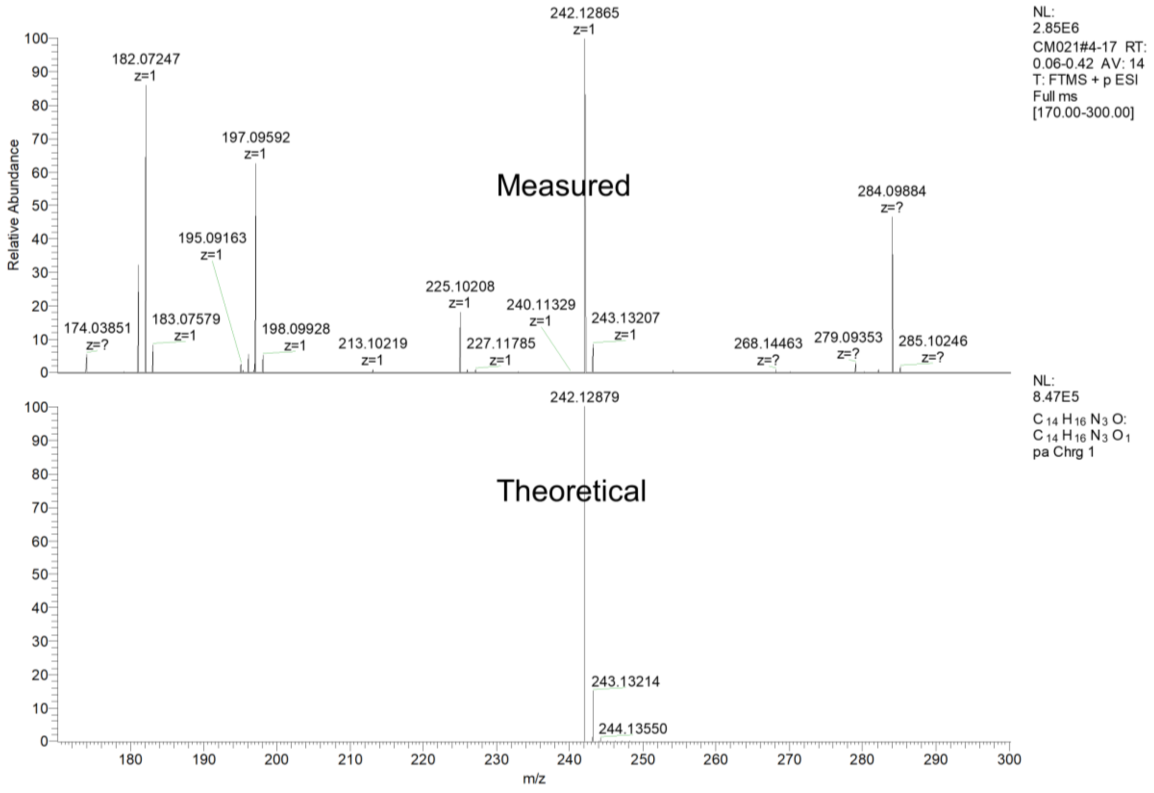
Measured
Theoretical
Relative Abundance
m/z
242.12865 z=1
182.07247 z=1
197.09592 z=1
284.09884 z=?
195.09163 z=1
225.10208 z=1
240.11329 z=1
243.13207 z=1
174.03851 z=?
183.07579 z=1
198.09928 z=1
213.10219 z=1
227.11785 z=1
268.14463 z=?
279.09353 z=?
285.10246 z=?
NL: 2.85E6
CM021#4-17 RT: 0.06-0.42 AV: 14
T: FTMS + p ESI Full ms [170.00-300.00]
242.12879
243.13214
244.13550
NL: 8.47E5
C 14 H 16 N 3 O:
C 14 H 16 N 3 O 1
pa Chrg 1

Zoomed in

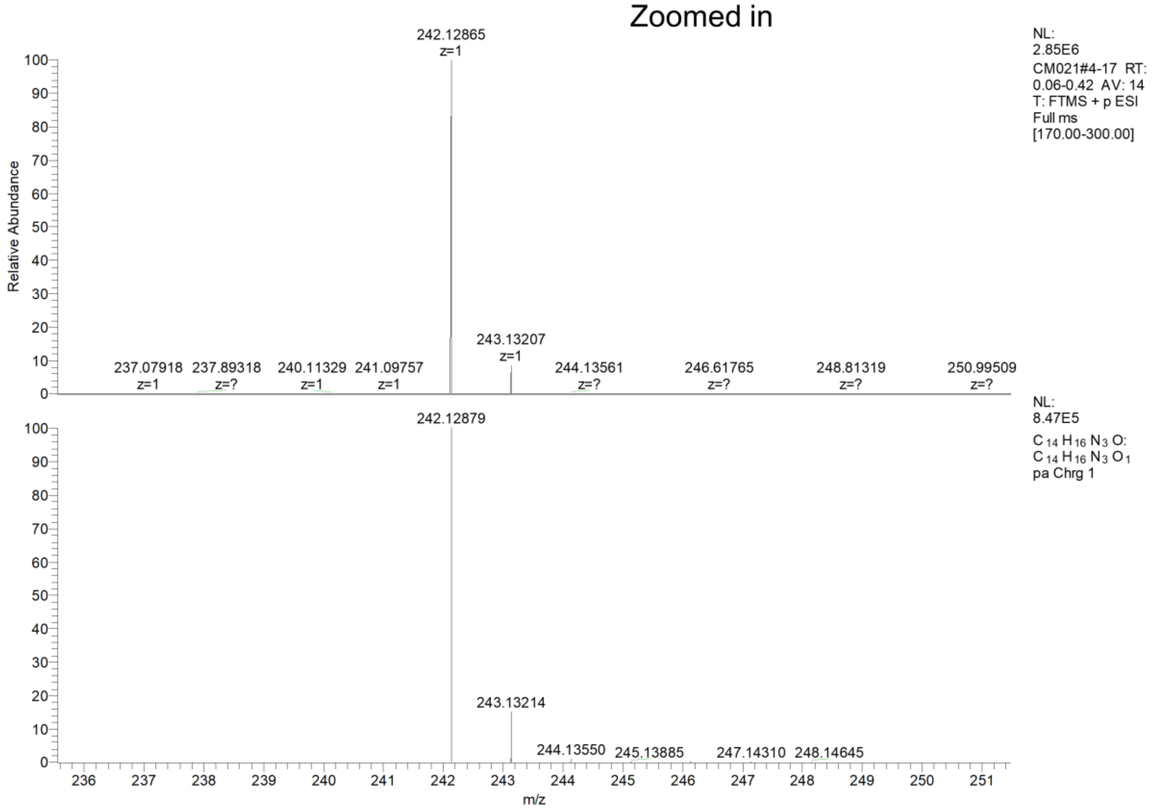
Relative Abundance
m/z
242.12865 z=1
243.13207 z=1
237.07918 z=1
237.89318 z=?
240.11329 z=1
241.09757 z=1
244.13561 z=?
246.61765 z=?
248.81319 z=?
250.99509 z=?
NL: 2.85E6
CM021#4-17 RT: 0.06-0.42 AV: 14
T: FTMS + p ESI Full ms [170.00-300.00]
242.12879
243.13214
244.13550
245.13885
247.14310
248.14645
NL: 8.47E5
C 14 H 16 N 3 O:
C 14 H 16 N 3 O 1
pa Chrg 1

(*E*)-9-isopropyl-N-(3-((4-methoxyphenyl)diazenyl)benzyl)-9H-purin-6-amine (**6**)

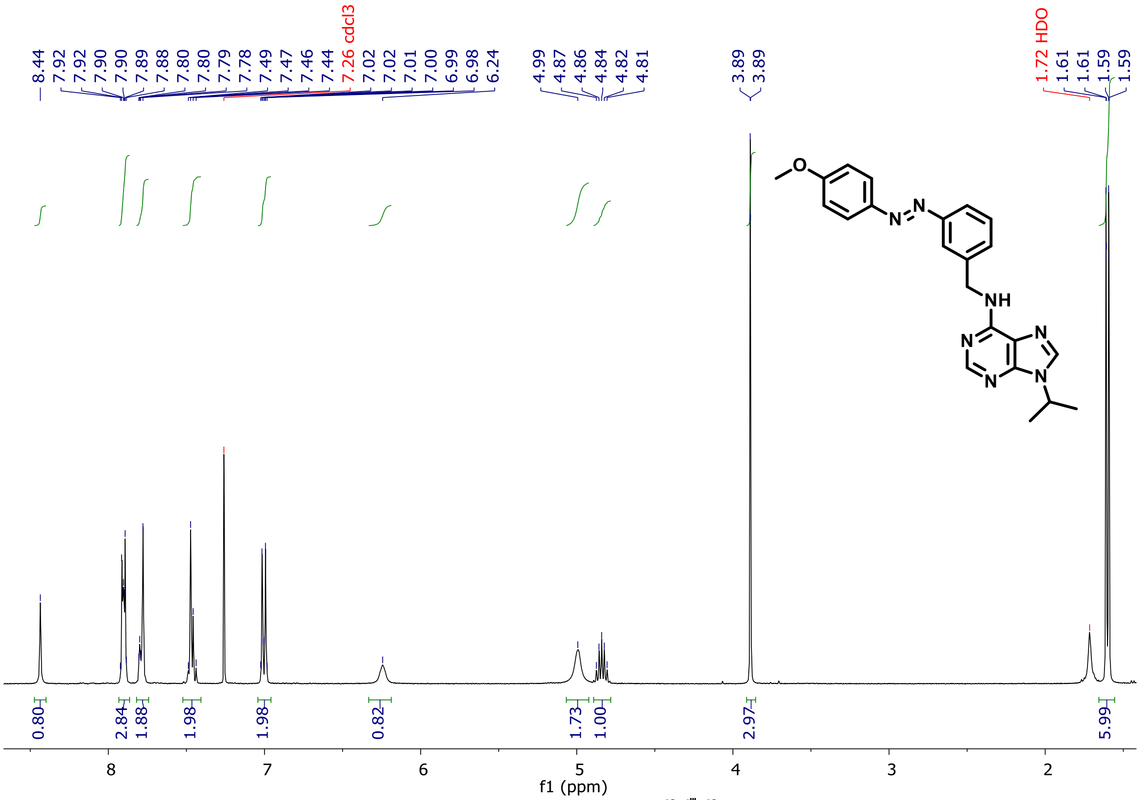

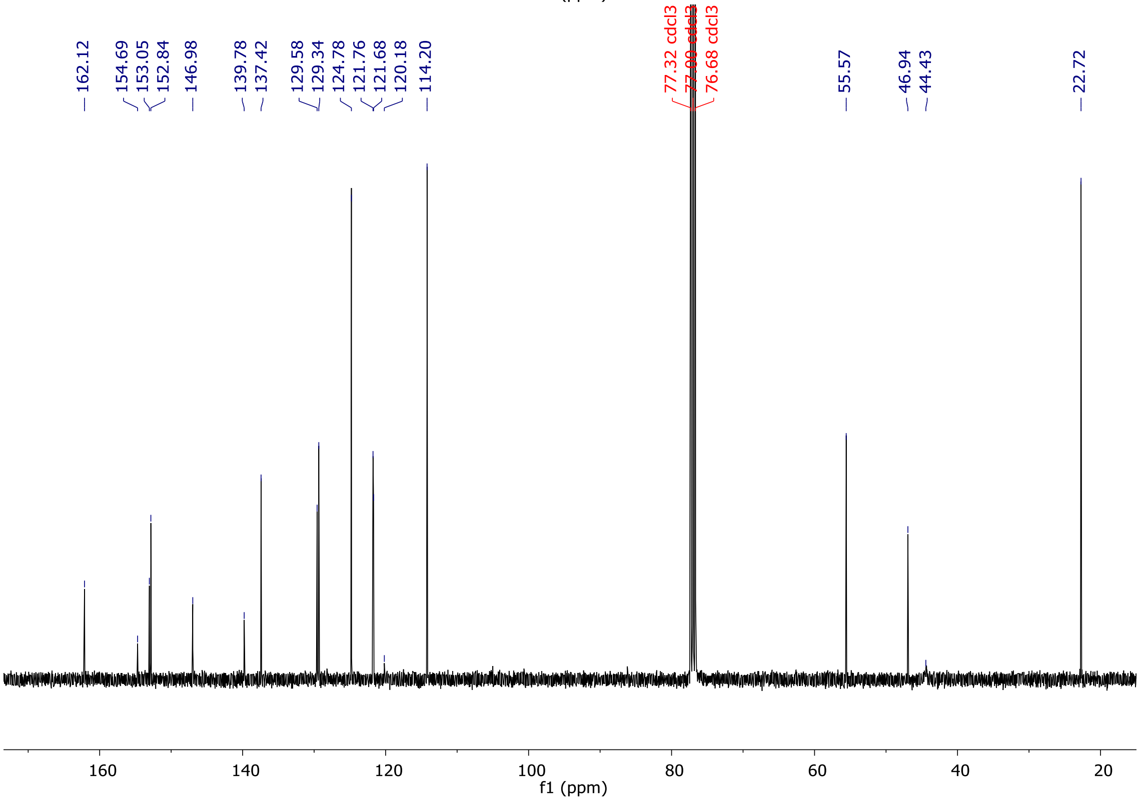

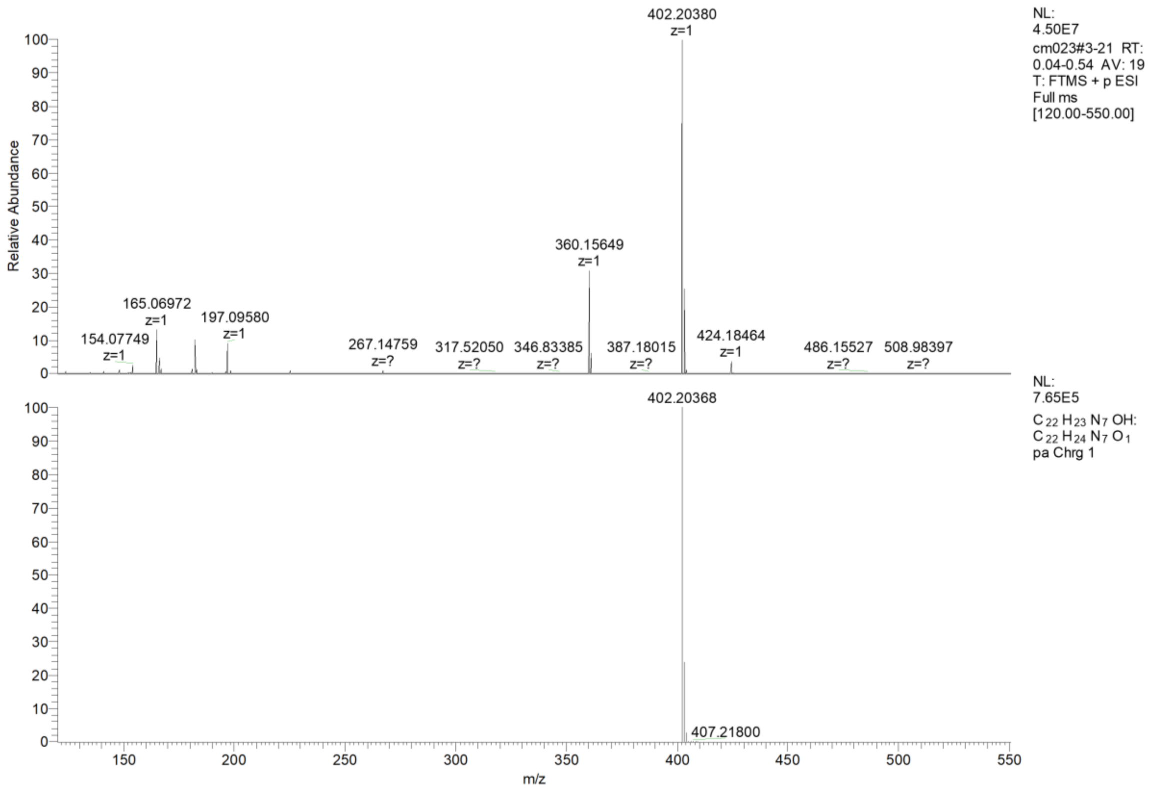
402.20380
z=1
NL:
4.50E7
cm023#3-21 RT:
0.04-0.54 AV: 19
T: FTMS + p ESI
Full ms
[120.00-550.00]
360.15649
z=1
165.06972
z=1
197.09580
z=1
154.07749
z=1
267.14759
z=?
317.52050
z=?
346.83385
z=?
387.18015
z=?
424.18464
z=1
486.15527
z=?
508.98397
z=?
Relative Abundance
NL:
7.65E5
402.20368
C 22 H 23 N 7 OH:
C 22 H 24 N 7 O 1
pa Chrg 1
407.21800
m/z

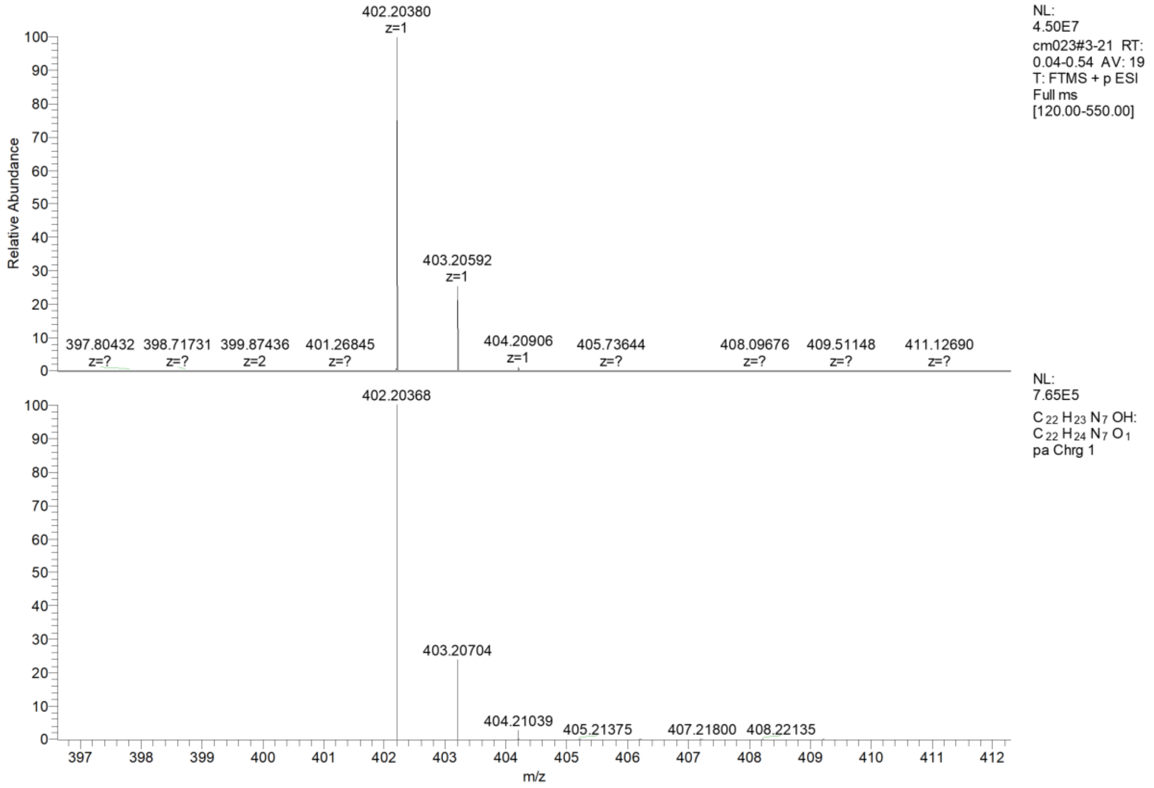
402.20380
z=1
NL:
4.50E7
cm023#3-21 RT:
0.04-0.54 AV: 19
T: FTMS + p ESI
Full ms
[120.00-550.00]
403.20592
z=1
397.80432
z=?
398.71731
z=?
399.87436
z=2
401.26845
z=?
404.20906
z=1
405.73644
z=?
408.09676
z=?
409.51148
z=?
411.12690
z=?
Relative Abundance
NL:
7.65E5
402.20368
C 22 H 23 N 7 OH:
C 22 H 24 N 7 O 1
pa Chrg 1
403.20704
404.21039
405.21375
407.21800
408.22135
m/z

(*E*)-N-(3-((2,6-difluorophenyl)diazenyl)benzyl)-9-isopropyl-9H-purin-6-amine (**7**)

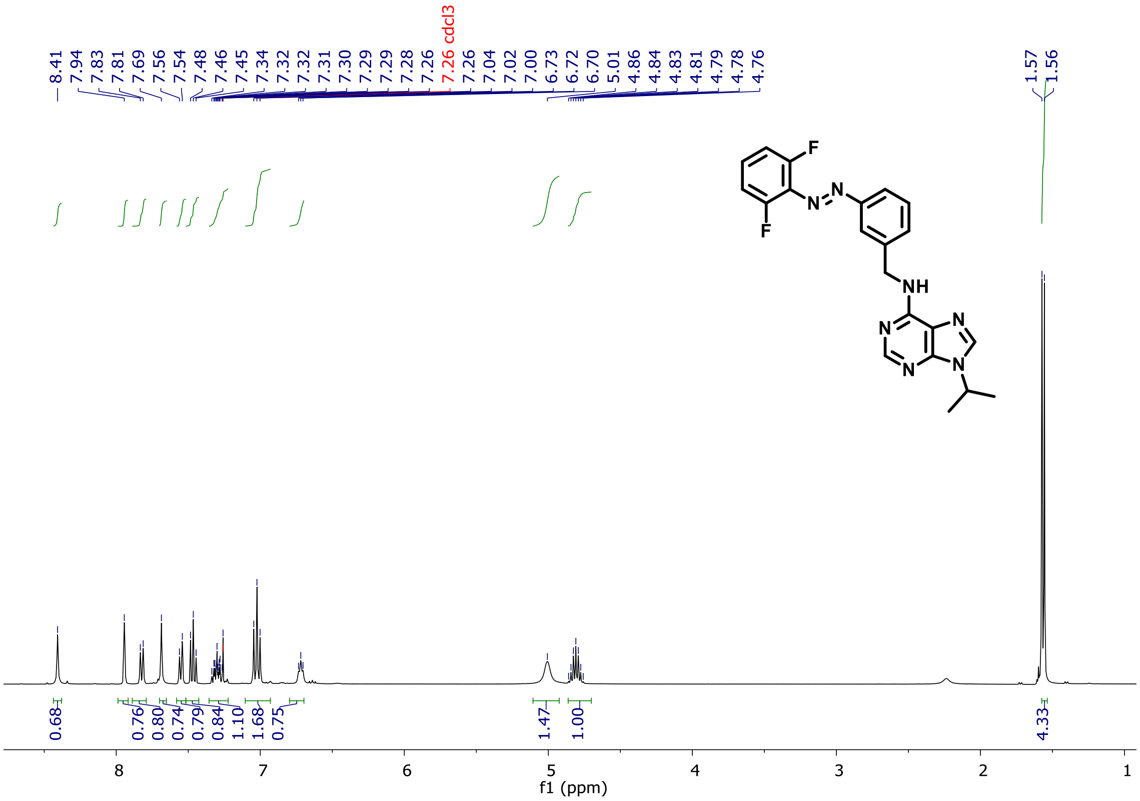

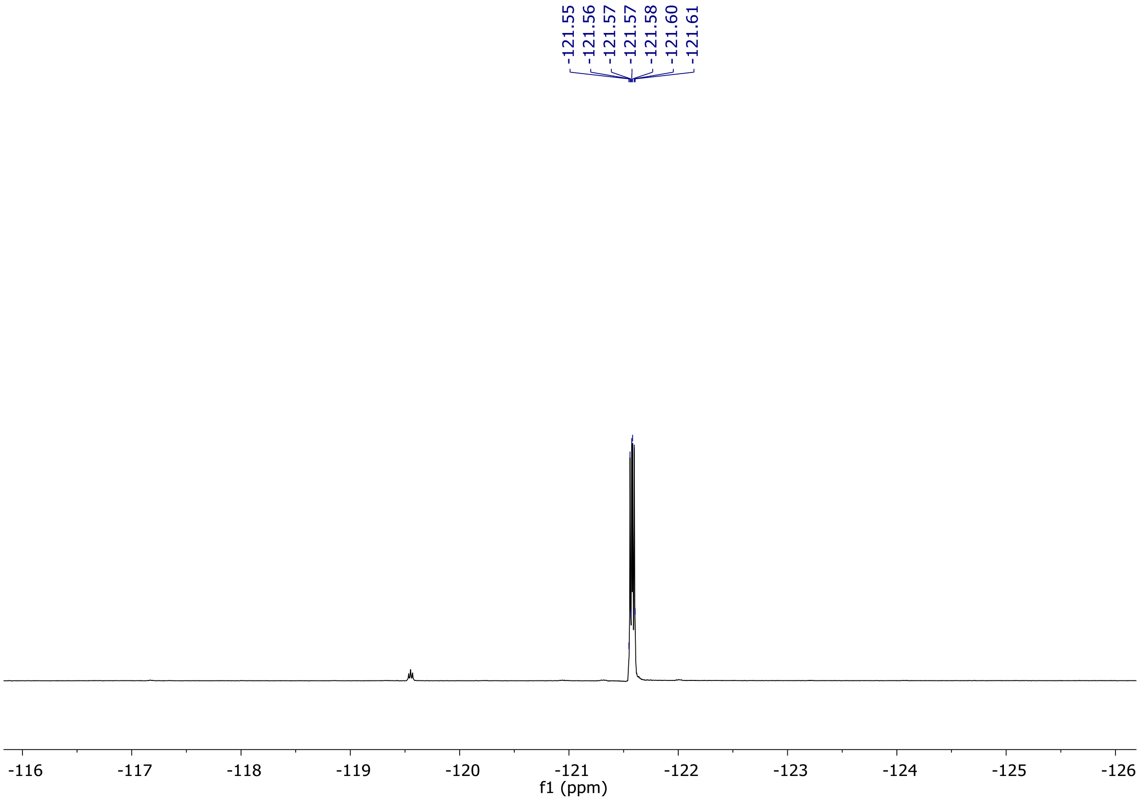

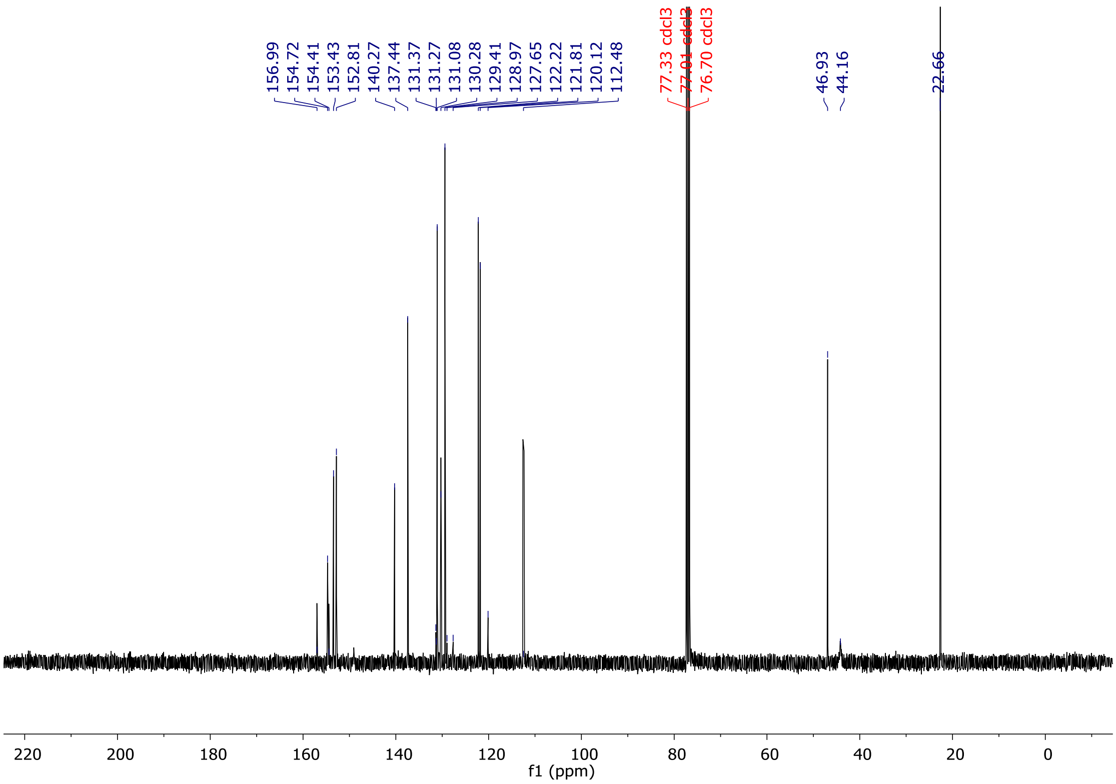
156.99
154.72
154.41
153.43
152.81
140.27
137.44
131.37
131.27
131.08
130.28
129.41
128.97
127.65
122.22
121.81
120.12
112.48
77.33 cdcl3
77.01 cdcl3
76.70 cdcl3
46.93
44.16
22.66
220
200
180
160
140
120
100
80
60
40
20
0
f1 (ppm)

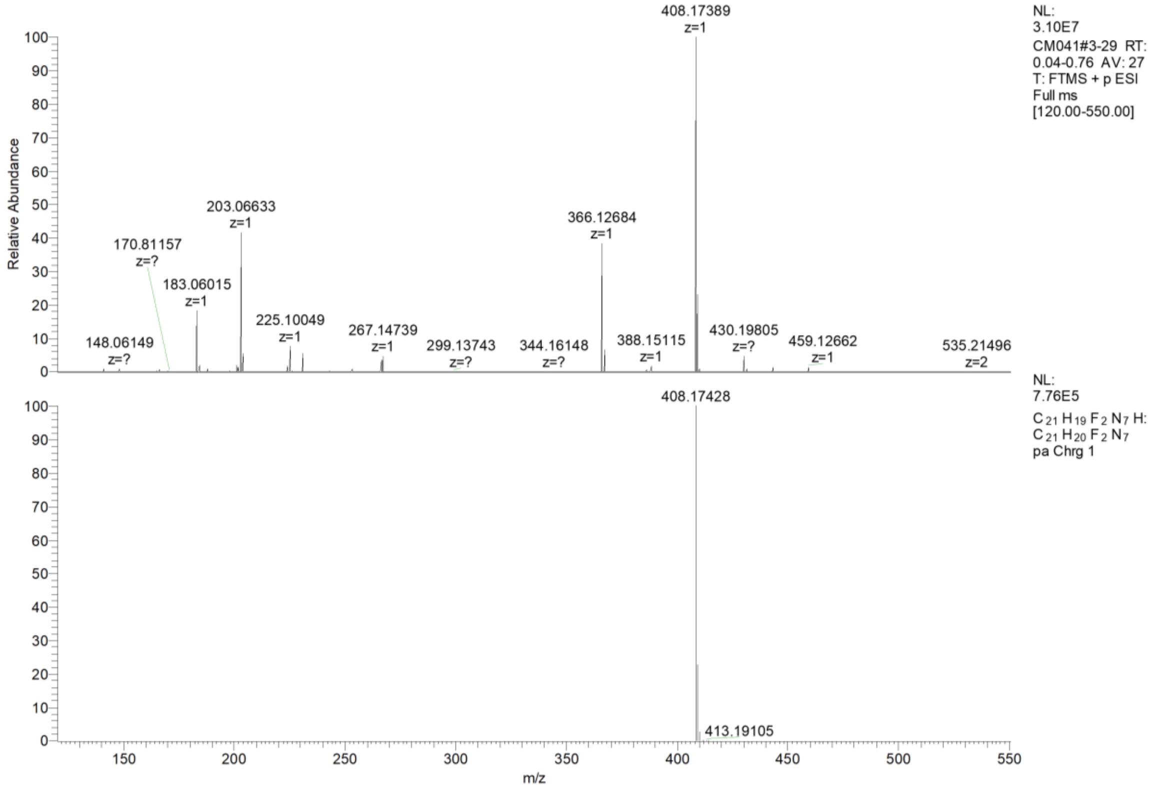
408.17389 z=1
NL: 3.10E7 CM041#3-29 RT: 0.04-0.76 AV: 27 T: FTMS + p ESI Full ms [120.00-550.00]
Relative Abundance
203.06633 z=1
366.12684 z=1
170.81157 z=?
183.06015 z=1
225.10049 z=1
267.14739 z=1
148.06149 z=?
299.13743 z=?
344.16148 z=?
388.15115 z=1
430.19805 z=?
459.12662 z=1
535.21496 z=2
NL: 7.76E5 C 21 H 19 F 2 N 7 H: C 21 H 20 F 2 N 7 pa Chrg 1
408.17428
413.19105
m/z

Zoomed in

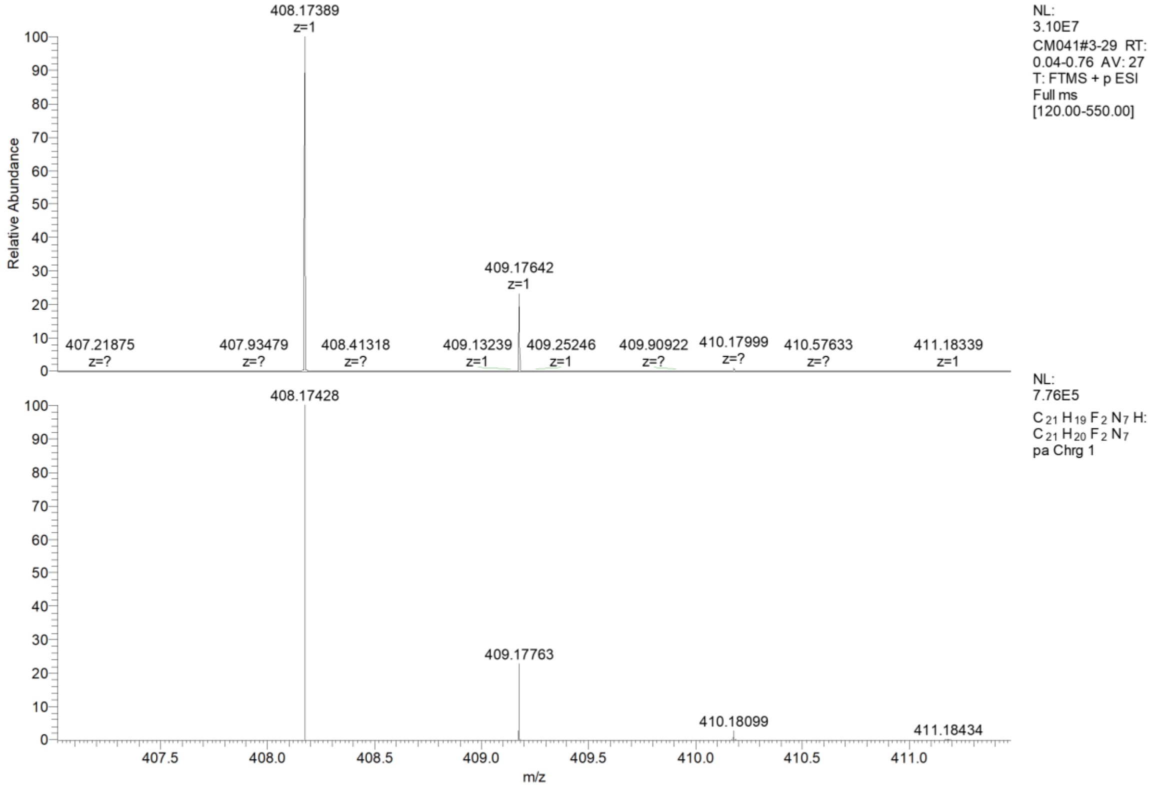
408.17389 z=1
NL: 3.10E7 CM041#3-29 RT: 0.04-0.76 AV: 27 T: FTMS + p ESI Full ms [120.00-550.00]
Relative Abundance
409.17642 z=1
407.21875 z=?
407.93479 z=?
408.41318 z=?
409.13239 z=1
409.25246 z=1
409.90922 z=?
410.17999 z=?
410.57633 z=?
411.18339 z=1
NL: 7.76E5 C 21 H 19 F 2 N 7 H: C 21 H 20 F 2 N 7 pa Chrg 1
408.17428
409.17763
410.18099
411.18434
m/z

(*E*)-9-isopropyl-*N*-(3-((1,3,5-trimethyl-1*H*-pyrazol-4-yl)diazenyl)benzyl)-9*H*-purin-6-amine (**8**)

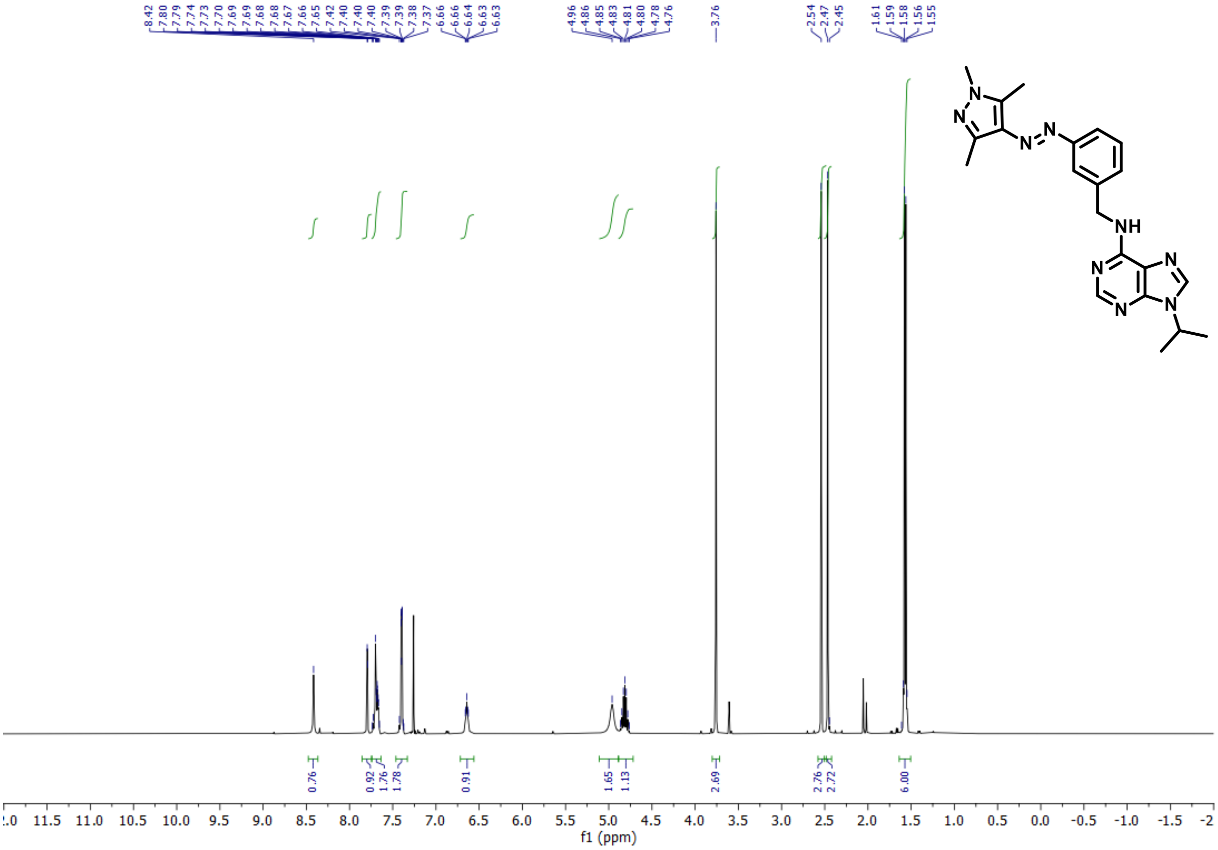

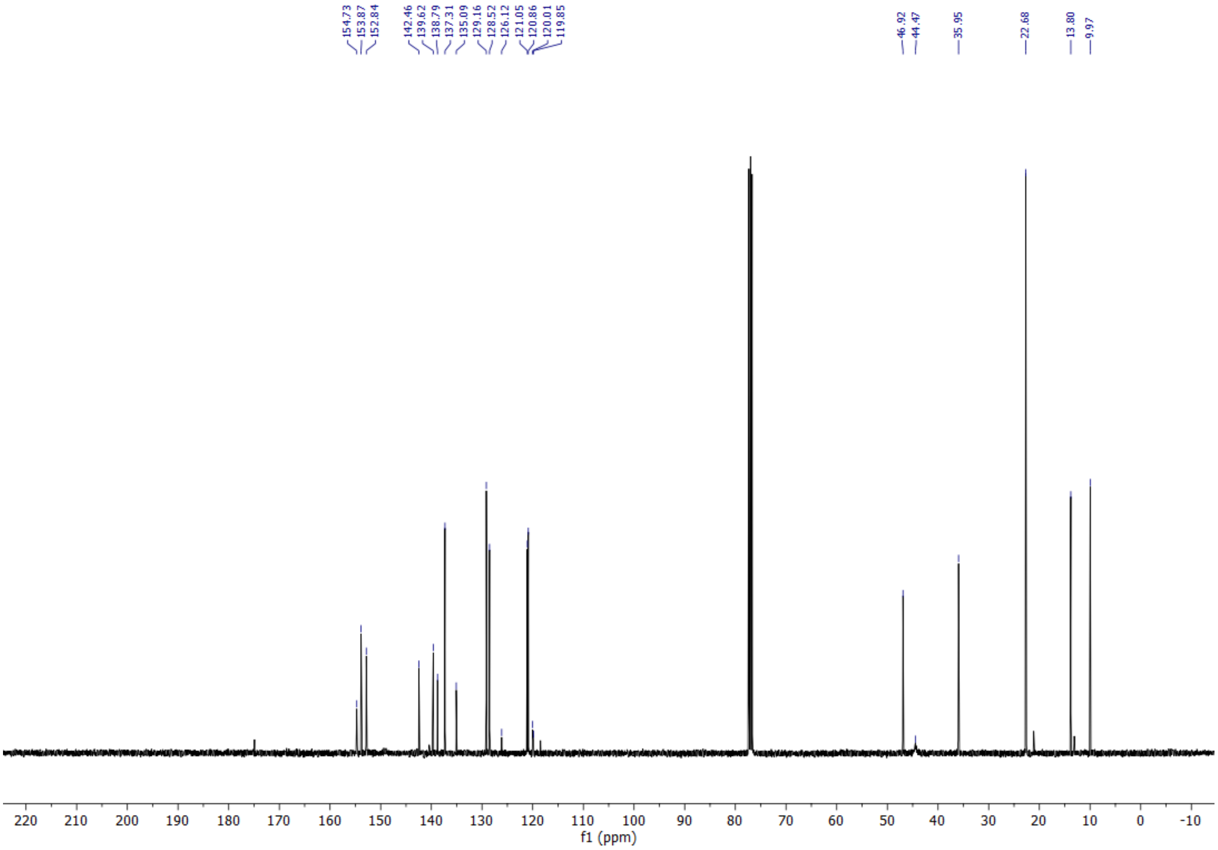

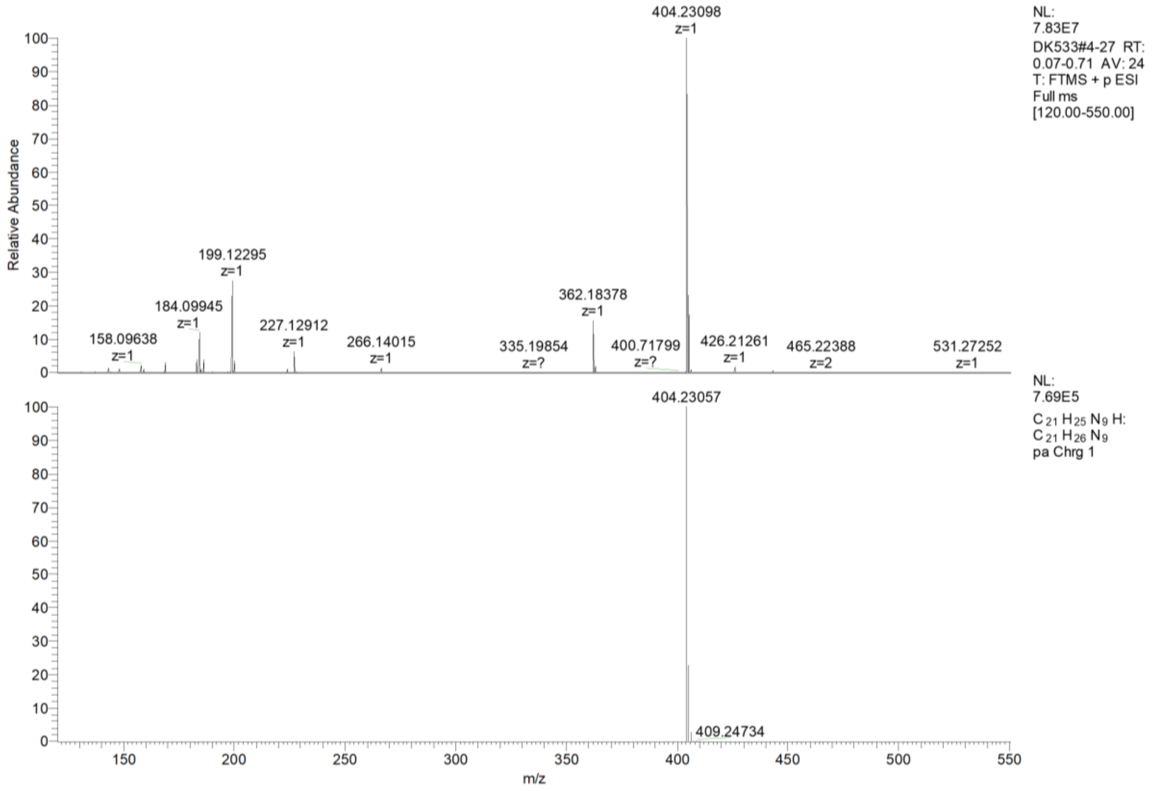
404.23098 z=1
199.12295 z=1
184.09945 z=1
227.12912 z=1
158.09638 z=1
266.14015 z=1
335.19854 z=?
362.18378 z=1
400.71799 z=?
426.21261 z=1
465.22388 z=2
531.27252 z=1
404.23057
409.24734
Relative Abundance
m/z
NL: 7.83E7
DK533#4-27 RT: 0.07-0.71 AV: 24
T: FTMS + p ESI Full ms [120.00-550.00]
NL: 7.69E5
C 21 H 25 N 9 H: C 21 H 26 N 9
pa Chrg 1

Zoomed in

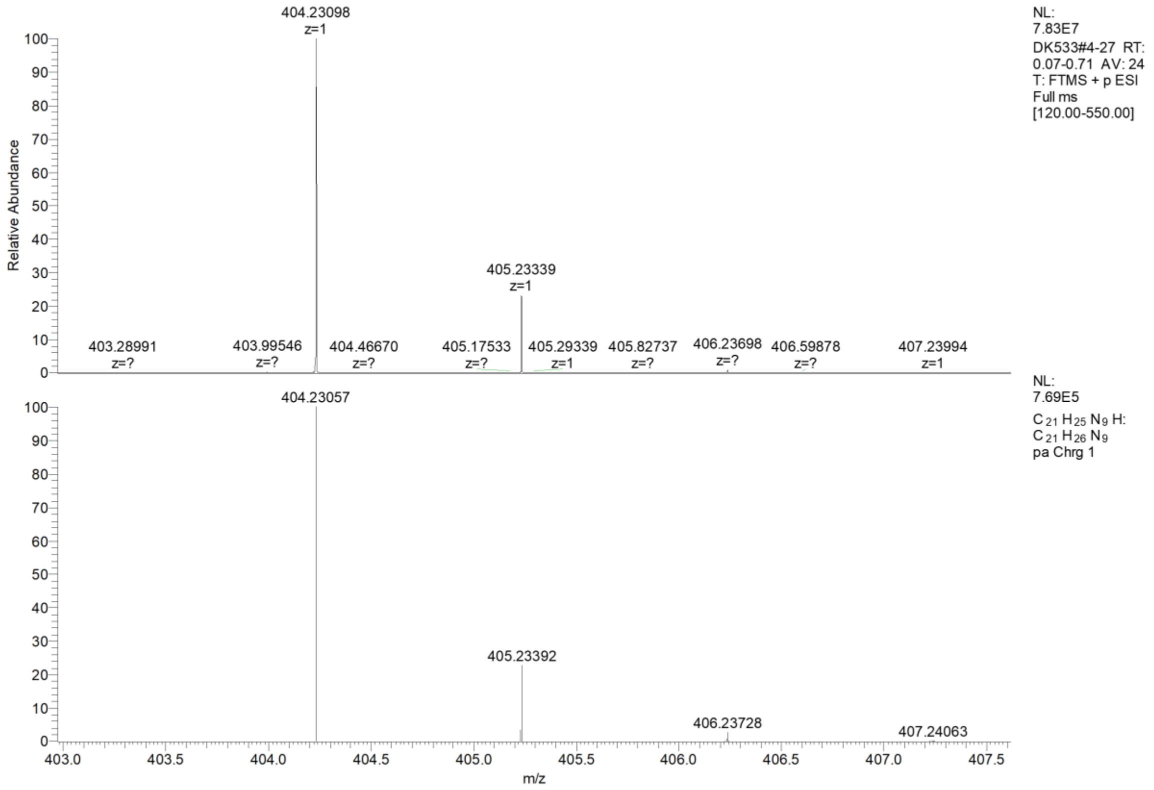
404.23098 z=1
403.28991 z=?
403.99546 z=?
404.46670 z=?
405.17533 z=?
405.23339 z=1
405.29339 z=1
405.82737 z=?
406.23698 z=?
406.59878 z=?
407.23994 z=1
404.23057
405.23392
406.23728
407.24063
Relative Abundance
m/z
NL: 7.83E7
DK533#4-27 RT: 0.07-0.71 AV: 24
T: FTMS + p ESI Full ms [120.00-550.00]
NL: 7.69E5
C 21 H 25 N 9 H: C 21 H 26 N 9
pa Chrg 1

(*E*)-1-(2,6-difluoro-3-methylphenyl)-2-(2,6-difluorophenyl)diazene (**22**)

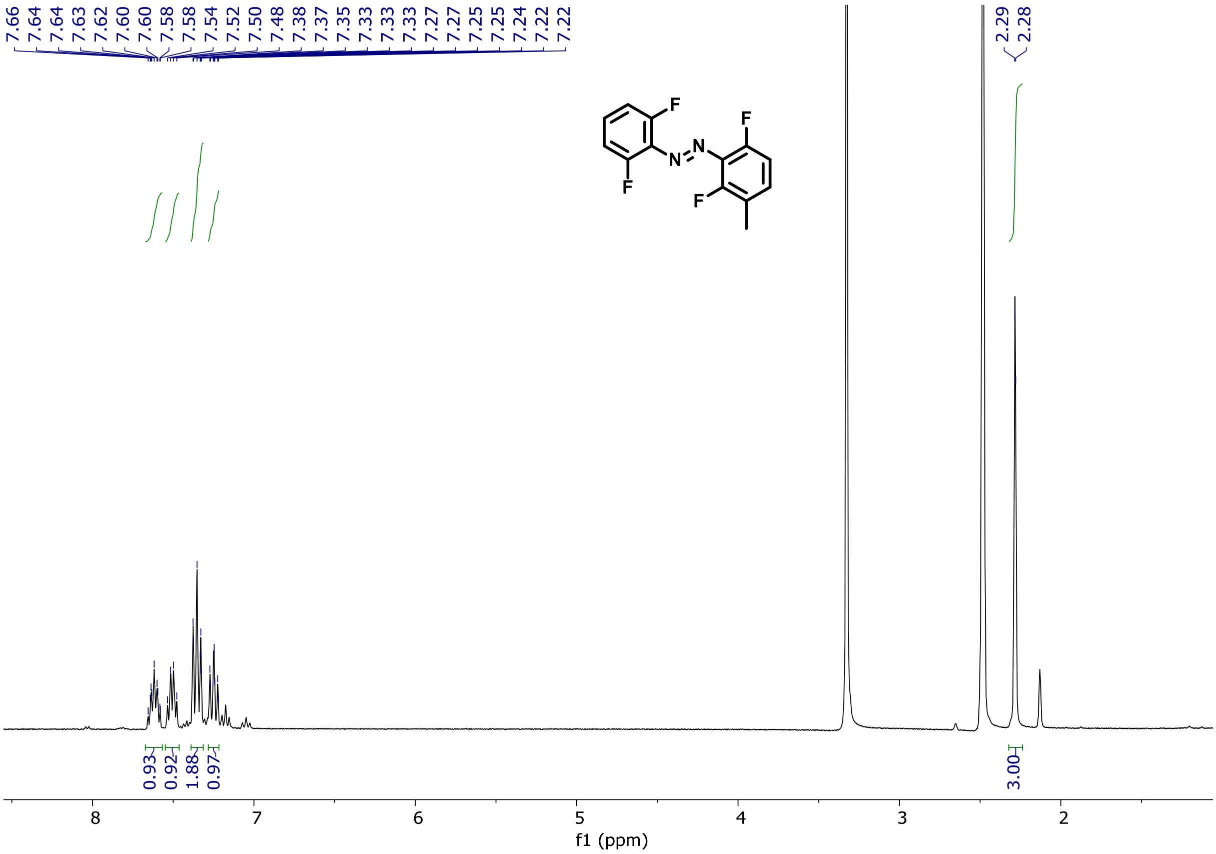

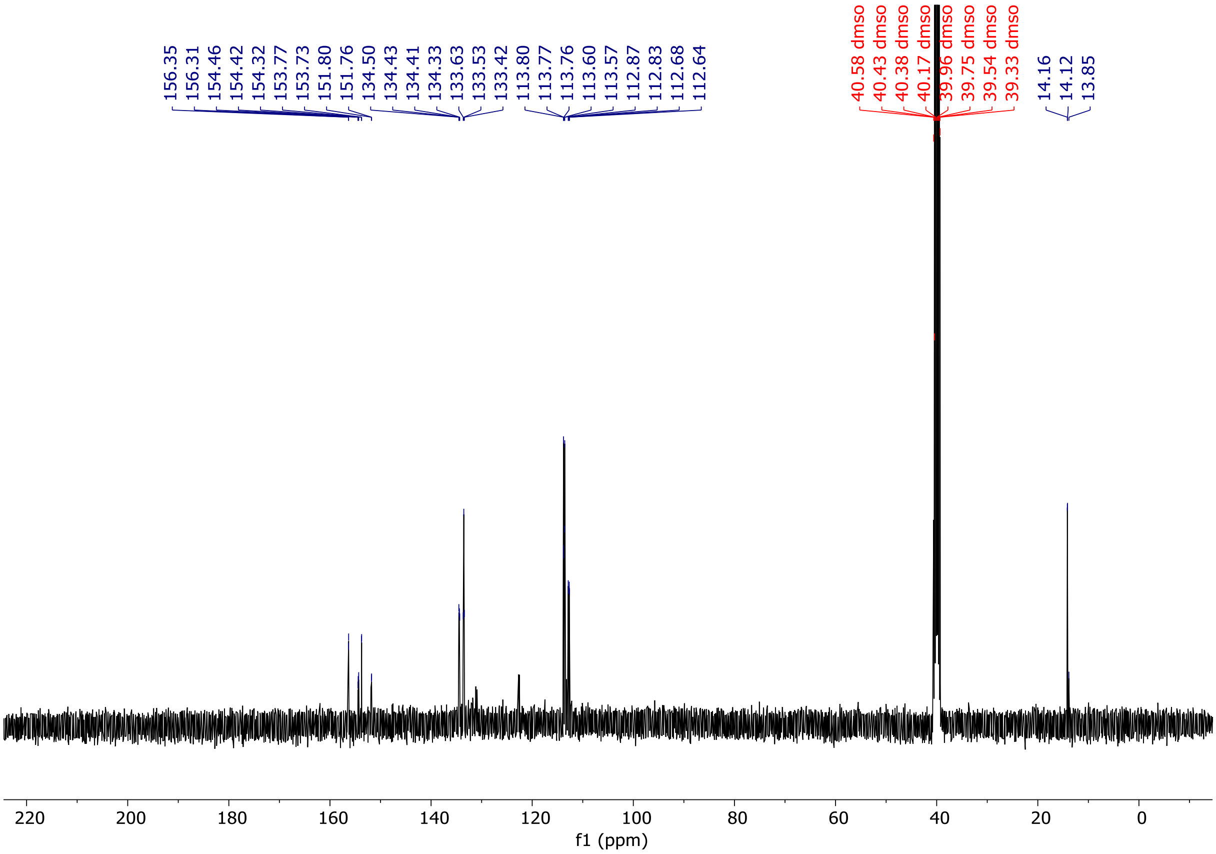

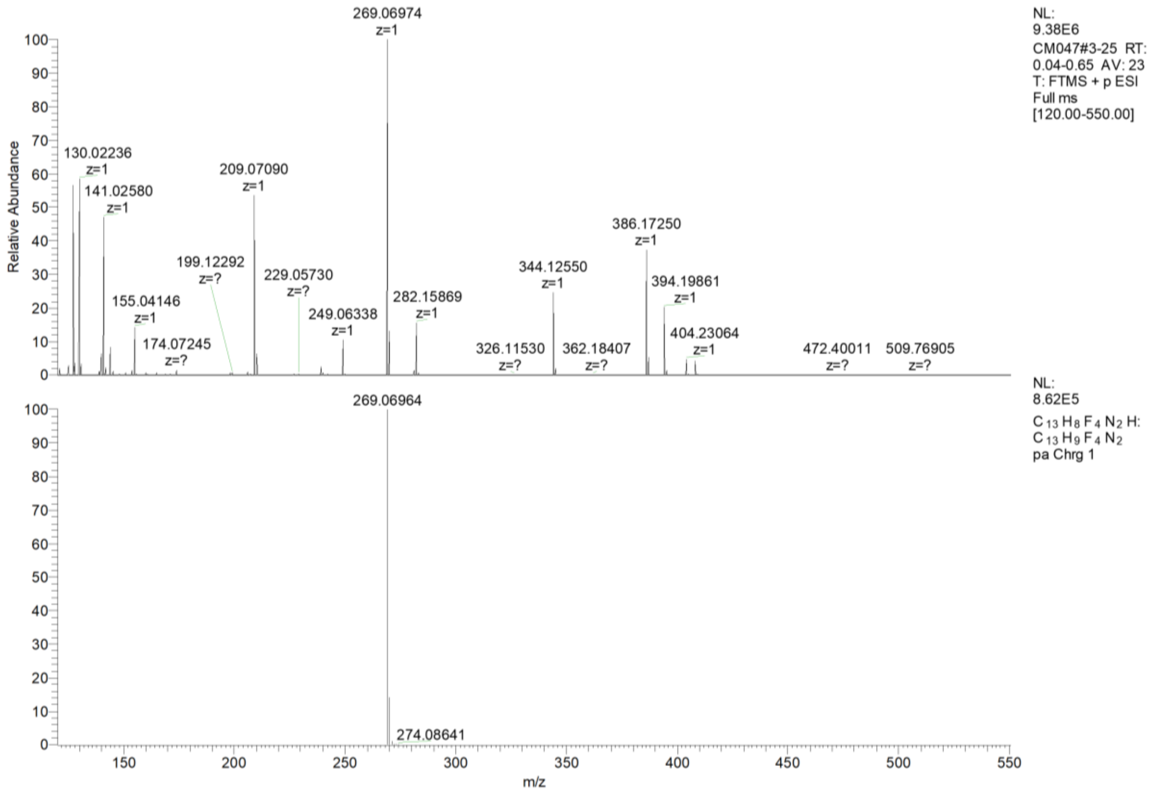
269.06974
z=1
NL:
9.38E6
CM047#3-25 RT:
0.04-0.65 AV: 23
T: FTMS + p ESI
Full ms
[120.00-550.00]
Relative Abundance
130.02236
z=1
141.02580
z=1
209.07090
z=1
199.12292
z=?
229.05730
z=?
155.04146
z=1
174.07245
z=?
249.06338
z=1
282.15869
z=1
344.12550
z=1
386.17250
z=1
394.19861
z=1
404.23064
z=1
326.11530
z=?
362.18407
z=?
472.40011
z=?
509.76905
z=?
NL:
8.62E5
C 13 H 8 F 4 N 2 H:
C 13 H 9 F 4 N 2
pa Chrg 1
269.06964
274.08641
m/z

Zoomed in

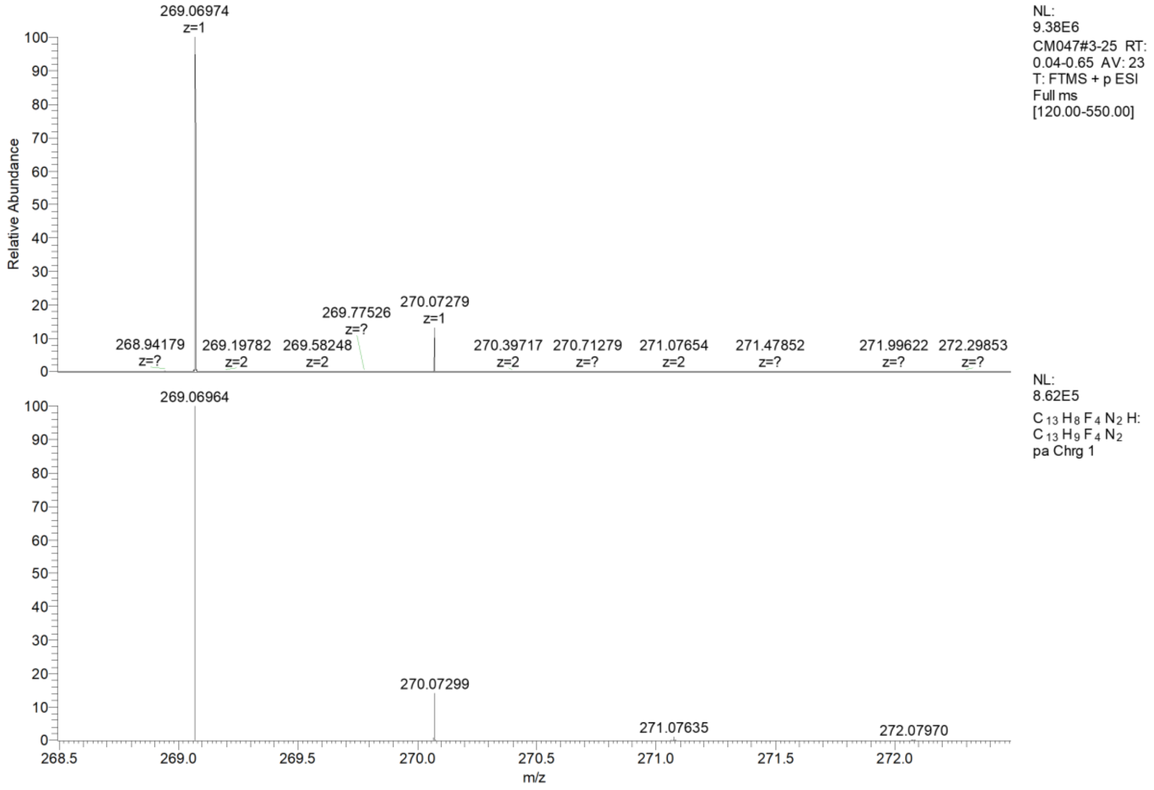
269.06974
z=1
NL:
9.38E6
CM047#3-25 RT:
0.04-0.65 AV: 23
T: FTMS + p ESI
Full ms
[120.00-550.00]
Relative Abundance
269.77526
z=?
270.07279
z=1
268.94179
z=?
269.19782
z=2
269.58248
z=2
270.39717
z=2
270.71279
z=?
271.07654
z=2
271.47852
z=?
271.99622
z=?
272.29853
z=?
NL:
8.62E5
C 13 H 8 F 4 N 2 H:
C 13 H 9 F 4 N 2
pa Chrg 1
269.06964
270.07299
271.07635
272.07970
m/z

(*E*)-1-(3-(bromomethyl)-2,6-difluorophenyl)-2-(2,6-difluorophenyl)diazene (**23**)

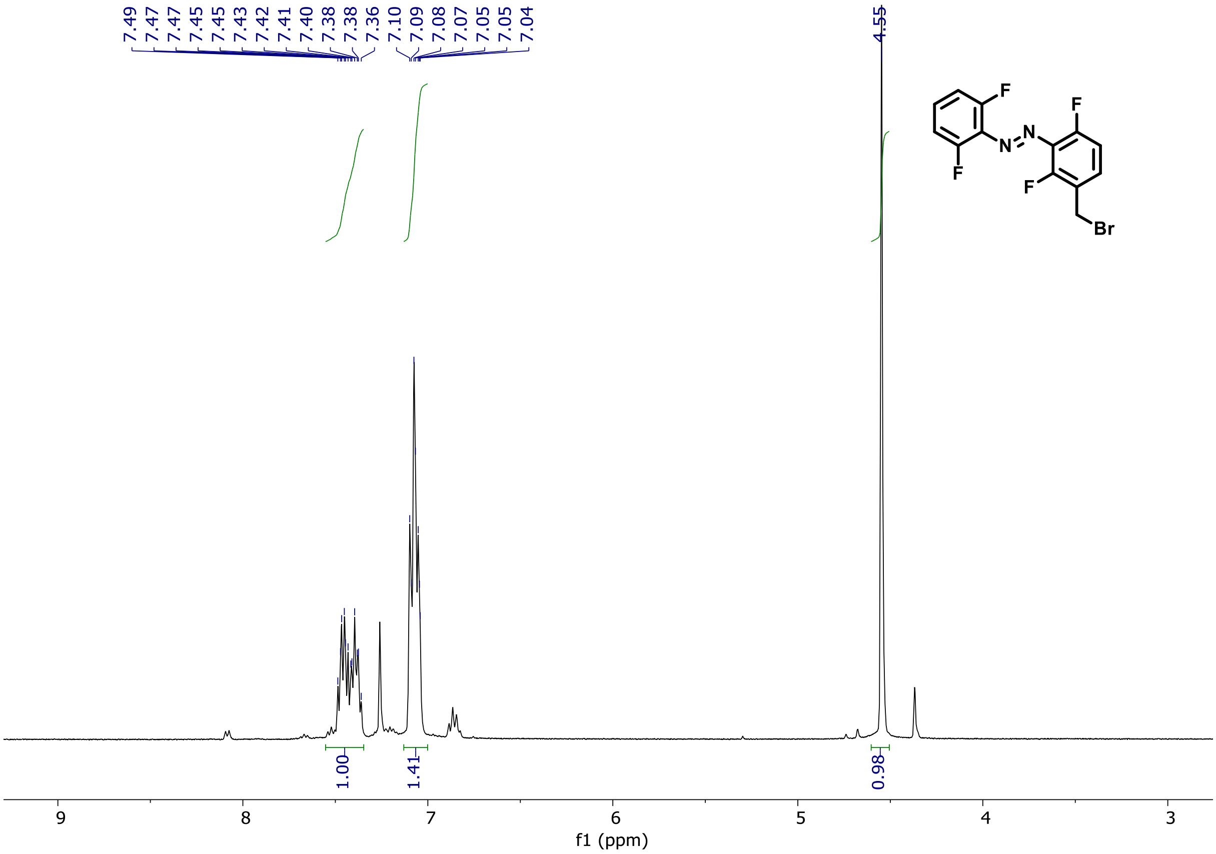

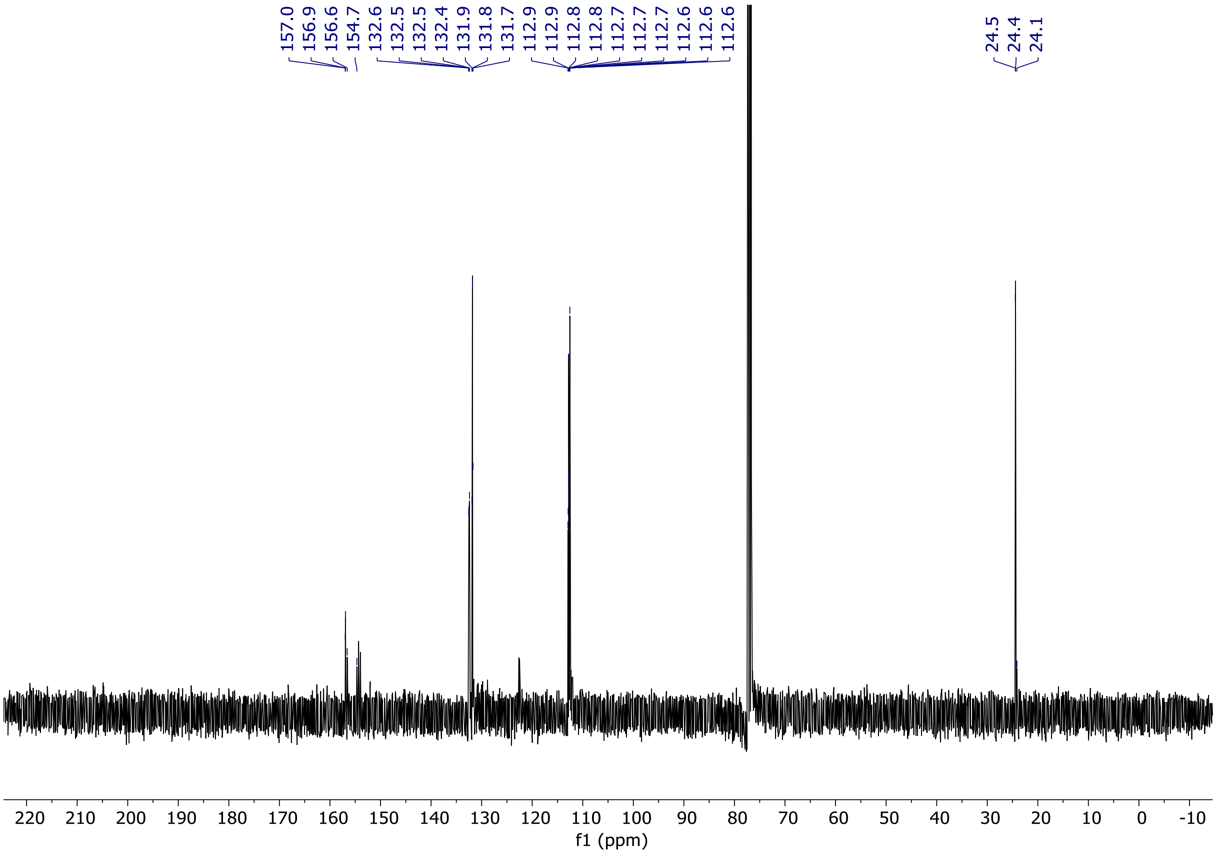

(*E*)-(3-((2,6-difluorophenyl)diazenyl)-2,4-difluorophenyl)methanamine (**24**)

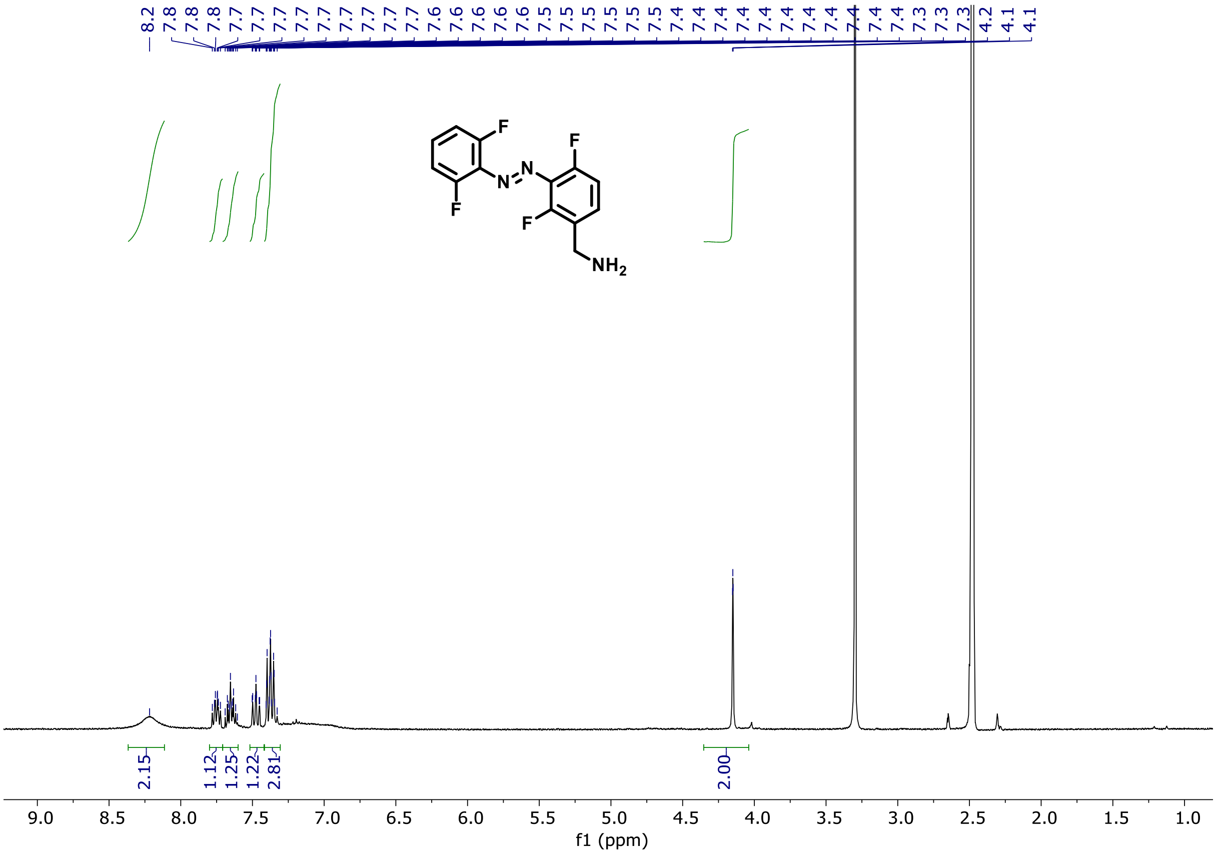

(*E*)-N-(3-((2,6-difluorophenyl)diazenyl)-2,4-difluorobenzyl)-9-isopropyl-9H-purin-6-amine (**9**)

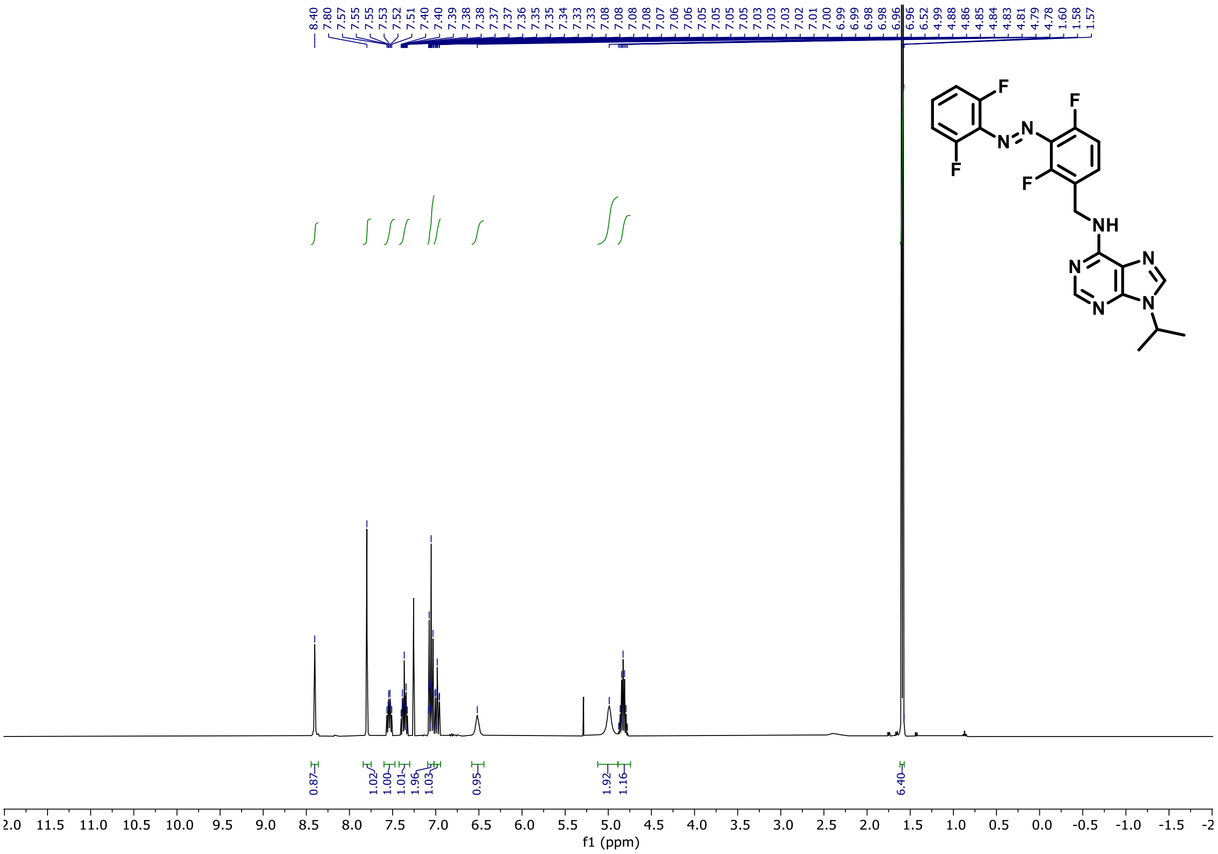

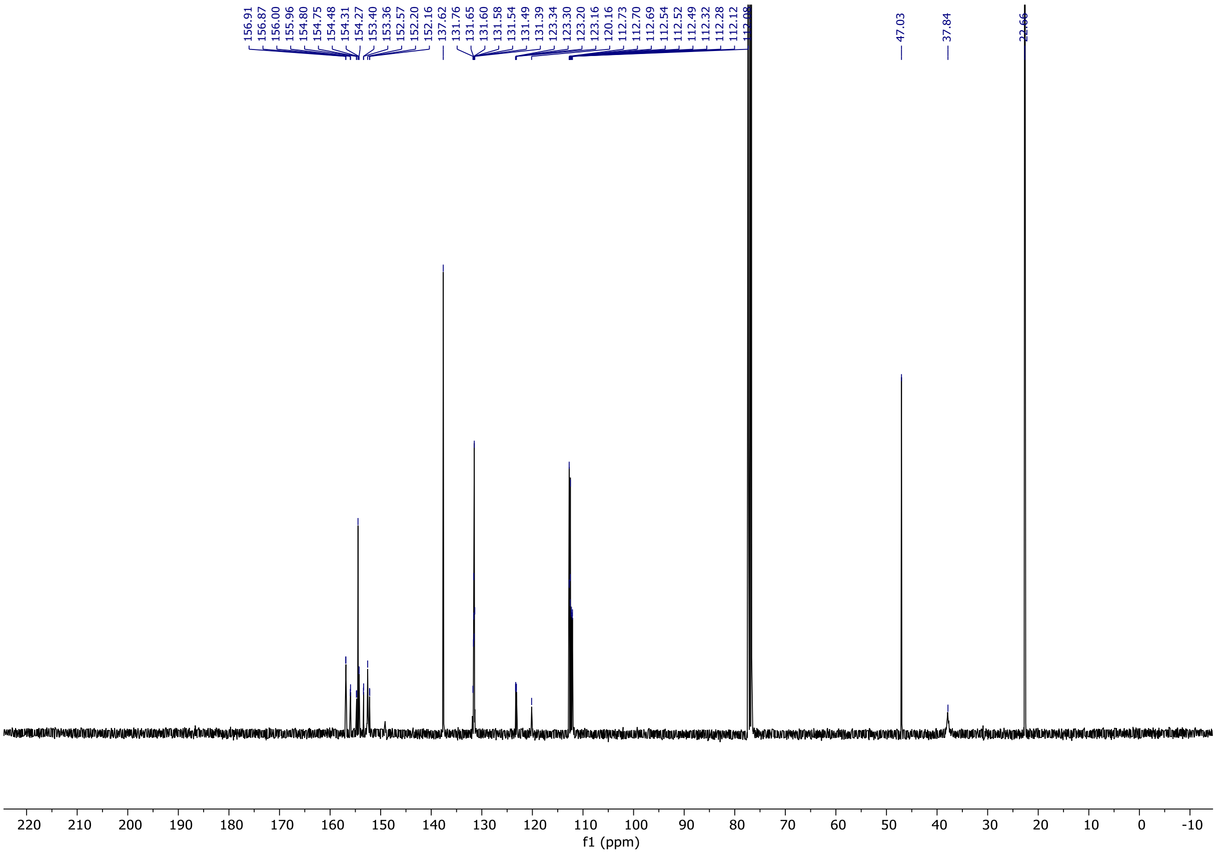

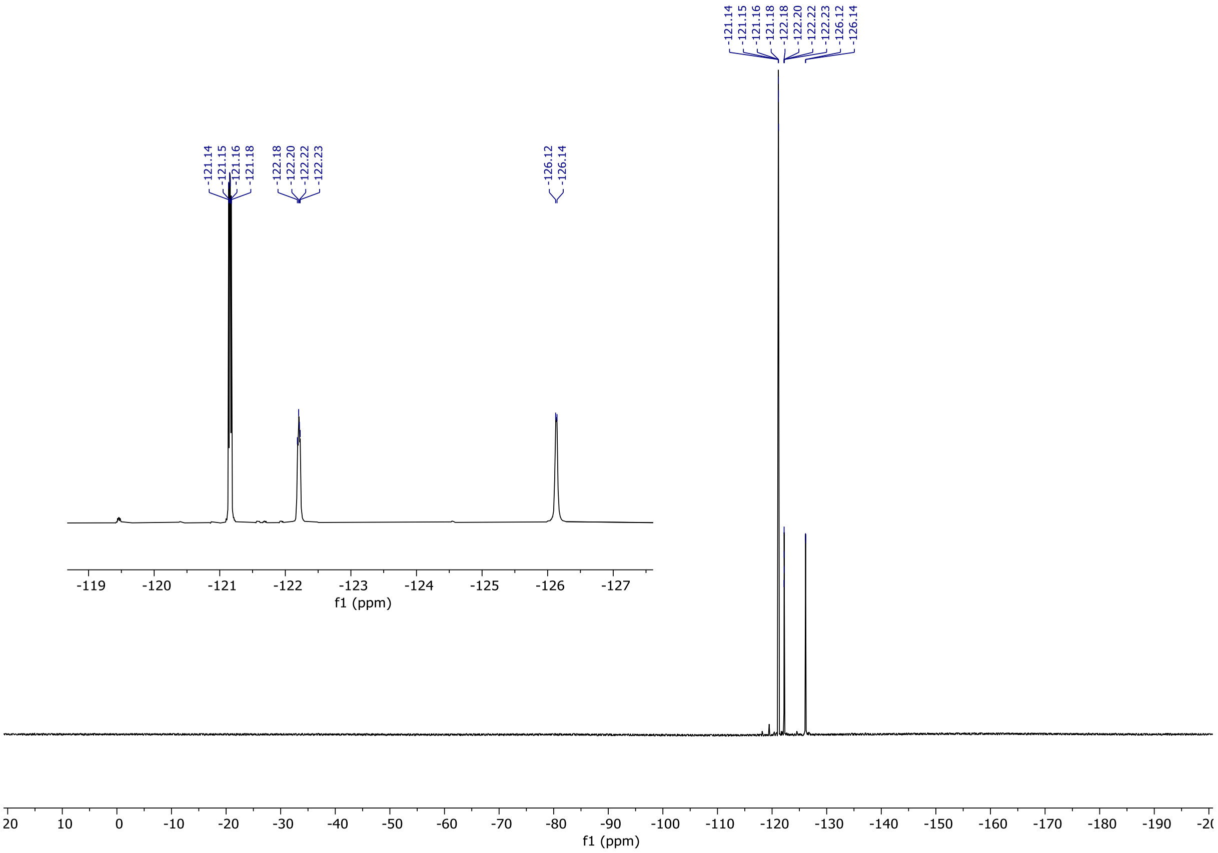
-121.14
-121.15
-121.16
-121.18
-122.18
-122.20
-122.22
-122.23
-126.12
-126.14
f1 (ppm)
f1 (ppm)

ESI pos

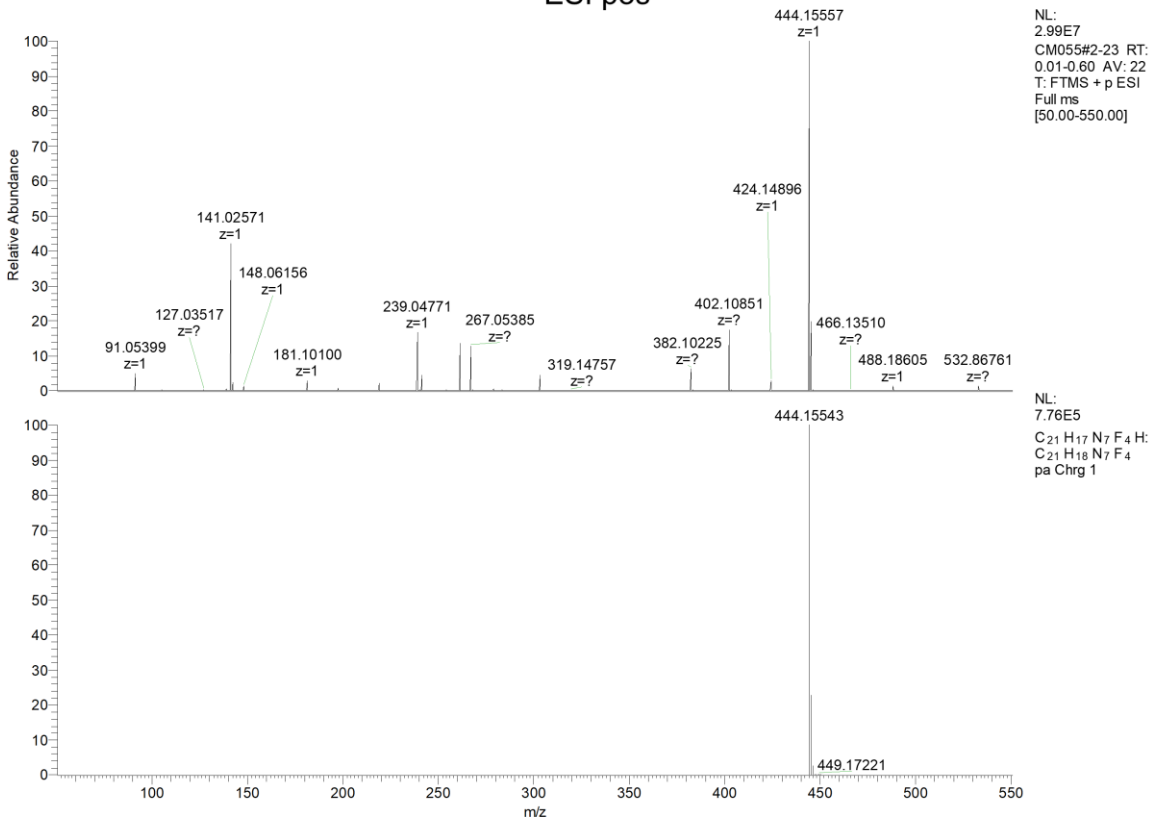

Zoomed in

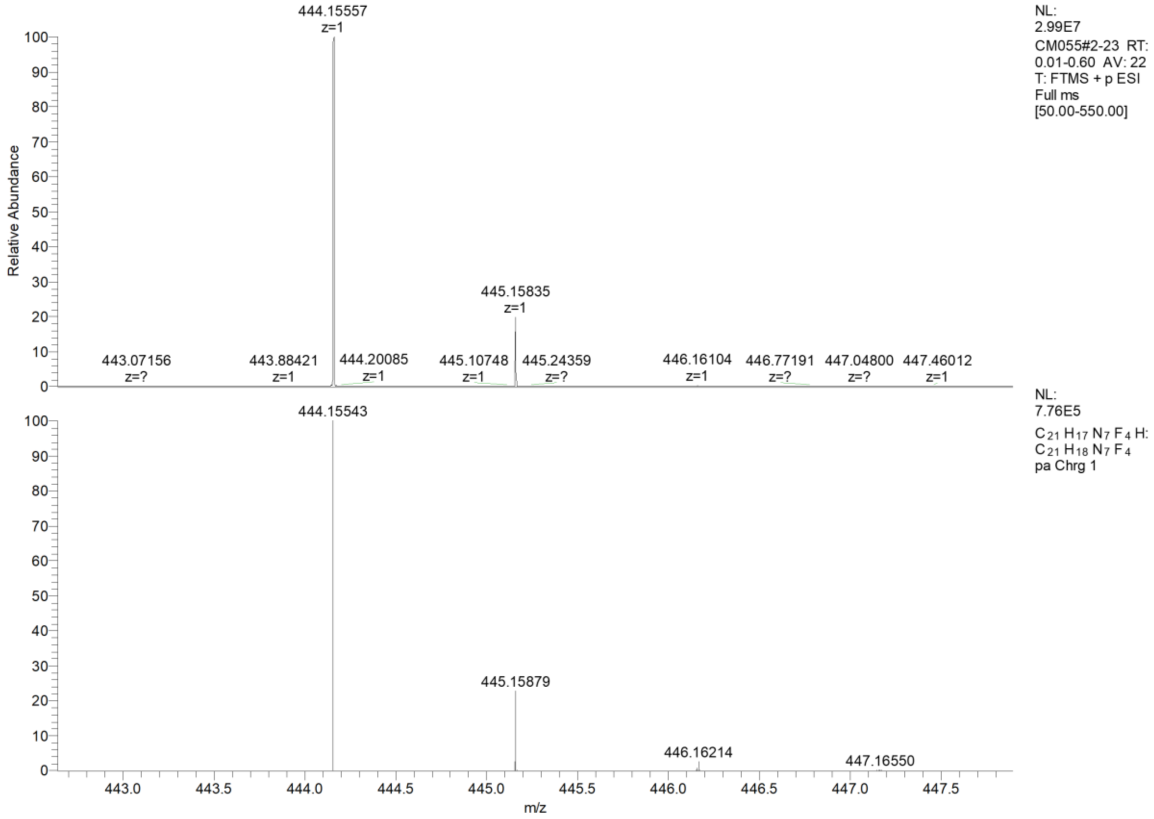

Supplementary References

1. Zhang, Q. *et al.* Discovery of 3-(((9H-purin-6-yl)amino)methyl)-4,6-dimethylpyridin-2(1H)-one derivatives as novel tubulin polymerization inhibitors for treatment of cancer. *Eur. J. Med. Chem.* **184**, 111728 (2019).

2. Jorda, R. *et al.* Anti-leishmanial activity of disubstituted purines and related pyrazolo[4,3-d]pyrimidines. *Bioorganic Med. Chem. Lett.* **21**, 4233–4237 (2011).

3. Hansen, M. J., Lerch, M. M., Szymanski, W. & Feringa, B. L. Direct and Versatile Synthesis of Red-Shifted Azobenzenes. *Angew. Chemie Int. Ed.* **55**, 13514–13518 (2016).

4. Hirota, T. *et al.* High-Throughput Chemical Screen Identifies a Novel Potent Modulator of Cellular Circadian Rhythms and Reveals CKIα as a Clock Regulatory Kinase. *PLoS Biol.* **8**, e1000559 (2010).
5. Oshima, T. *et al*. Cell-based screen identifies a new potent and highly selective CK2 inhibitor for modulation of circadian rhythms and cancer cell growth. *Sci. Adv*. **5**, eaau9060 (2019).

6. Hou, T., Wang, J., Li, Y. & Wang, W. Assessing the Performance of the MM/PBSA and MM/GBSA Methods. 1. The Accuracy of Binding Free Energy Calculations Based on Molecular Dynamics Simulations. *J. Chem. Inf. Model.* **51**, 69–82 (2011).

7. Genheden, S. & Ryde, U. The MM/PBSA and MM/GBSA methods to estimate ligand-binding affinities. *Expert Opinion on Drug Discovery* **10**, 449–461 (2015).

8. Alonso, H., Bliznyuk, A. A. & Gready, J. E. Combining Docking and Molecular Dynamic Simulations in Drug Design. *Med. Res. Rev.* **26**, 531–568 (2006).
