# Supplementary material for: Reversible modulation of circadian time with chronophotopharmacology
Source: Nat Commun. 2021 May 26;12:3164. doi: 10.1038/s41467-021-23301-x (PMC8155176; doi:10.1038/s41467-021-23301-x)
Supplement: Supplementary file 3 — Reporting Summary [file 41467_2021_23301_MOESM3_ESM.pdf]

nature research

Corresponding author(s): Ben L. Feringa, Tsuyoshi Hirota, Wiktor Szymanski

Last updated by author(s): Mar 18, 2021

Reporting Summary

Nature Research wishes to improve the reproducibility of the work that we publish. This form provides structure for consistency and transparency in reporting. For further information on Nature Research policies, see our Editorial Policies and the Editorial Policy Checklist.

Statistics

For all statistical analyses, confirm that the following items are present in the figure legend, table legend, main text, or Methods section.

n/a	Confirmed	
☐	☒	The exact sample size (*n*) for each experimental group/condition, given as a discrete number and unit of measurement
☐	☒	A statement on whether measurements were taken from distinct samples or whether the same sample was measured repeatedly
☐	☒	The statistical test(s) used AND whether they are one- or two-sided<br>*Only common tests should be described solely by name; describe more complex techniques in the Methods section.*
☒	☐	A description of all covariates tested
☒	☐	A description of any assumptions or corrections, such as tests of normality and adjustment for multiple comparisons
☐	☒	A full description of the statistical parameters including central tendency (e.g. means) or other basic estimates (e.g. regression coefficient) AND variation (e.g. standard deviation) or associated estimates of uncertainty (e.g. confidence intervals)
☐	☒	For null hypothesis testing, the test statistic (e.g. *F*, *t*, *r*) with confidence intervals, effect sizes, degrees of freedom and *P* value noted<br>*Give P values as exact values whenever suitable.*
☒	☐	For Bayesian analysis, information on the choice of priors and Markov chain Monte Carlo settings
☒	☐	For hierarchical and complex designs, identification of the appropriate level for tests and full reporting of outcomes
☒	☐	Estimates of effect sizes (e.g. Cohen's *d*, Pearson's *r*), indicating how they were calculated

Our web collection on statistics for biologists contains articles on many of the points above.

Software and code

Policy information about availability of computer code

Data collection	no software was used
Data analysis	MultiCycle (ver 1.503, Actimetrics) for circadian data analysis; Prism (ver 7.04, GraphPad Software) for curve fitting, Schrodinger Release 2018-4 for docking, Desmond Molecular Dynamics System, D. E. Shaw Research, New York, NY, 2018 for molecular dynamics simulations,

For manuscripts utilizing custom algorithms or software that are central to the research but not yet described in published literature, software must be made available to editors and reviewers. We strongly encourage code deposition in a community repository (e.g. GitHub). See the Nature Research guidelines for submitting code & software for further information.

Data

Policy information about availability of data

All manuscripts must include a data availability statement. This statement should provide the following information, where applicable:

- Accession codes, unique identifiers, or web links for publicly available datasets
- A list of figures that have associated raw data
- A description of any restrictions on data availability

The authors declare that the data supporting the findings of this study are available within the paper and its supplementary information files. Additional data on methods used are available from the corresponding author upon reasonable request.

Field-specific reporting

Please select the one below that is the best fit for your research. If you are not sure, read the appropriate sections before making your selection.

☒ Life sciences ☐ Behavioural & social sciences ☐ Ecological, evolutionary & environmental sciences

For a reference copy of the document with all sections, see nature.com/documents/nr-reporting-summary-flat.pdf

Life sciences study design

All studies must disclose on these points even when the disclosure is negative.

Sample size	To detect significant changes compared with control samples, we conducted dose-response experiments that consist of multiple data points with two or more samples for each point. Sample size was determined based on the similar analyses within the field.
Data exclusions	When the period length cannot be calculated because of low amplitude rhythms, we excluded the data from period plots. The exclusion criteria were pre-established in our previous studies.
Replication	We used two or more biological replicates. Key findings were confirmed with repeated experiments with independent samples and/or different approaches.
Randomization	Because no positional effect was observed on multi-well plates, cells and recombinant proteins dispensed evenly to each well were systematically allocated into experimental groups to analyze dose-dependency of the compounds (i.e., dilution series). Tissue pieces were randomly allocated into experimental groups.
Blinding	Chemical structure was blinded, and compounds were arranged in numerical order during data collection and analysis.

Reporting for specific materials, systems and methods

We require information from authors about some types of materials, experimental systems and methods used in many studies. Here, indicate whether each material, system or method listed is relevant to your study. If you are not sure if a list item applies to your research, read the appropriate section before selecting a response.

Materials & experimental systems

n/a	Involved in the study	
☒	☐	Antibodies
☐	☒	Eukaryotic cell lines
☒	☐	Palaeontology and archaeology
☐	☒	Animals and other organisms
☒	☐	Human research participants
☒	☐	Clinical data
☒	☐	Dual use research of concern

Methods

n/a	Involved in the study	
☒	☐	ChIP-seq
☒	☐	Flow cytometry
☒	☐	MRI-based neuroimaging

Eukaryotic cell lines

Policy information about cell lines

Cell line source(s)	Bmal1-dLuc U2OS reporter cell line was established in Hirota et al. 2008.
Authentication	Reporter of each cell line was confirmed by genomic PCR.
Mycoplasma contamination	Bmal1-dLuc U2OS cells were negative for mycoplasma contamination.
Commonly misidentified lines (See ICLAC register)	No commonly misidentified cell lines were used in the study.

Animals and other organisms

Policy information about studies involving animals; ARRIVE guidelines recommended for reporting animal research

Laboratory animals	Mus musculus, C57BL/6J Per2::Luc, male, newborn or 16-week-old.
Wild animals	The study did not involve wild animals.
Field-collected samples	The study did not involve samples collected from the field.

Ethics oversight	Animal Experiments Committees of Nagoya University.

Note that full information on the approval of the study protocol must also be provided in the manuscript.
